# Structures and receptor binding activities of merbecovirus spike proteins reveal key signatures for human DPP4 adaptation

**DOI:** 10.1126/sciadv.adv7296

**Published:** 2025-07-11

**Authors:** Hang Yuan, Jingjing Wang, Yong Ma, Zimu Li, Xijie Gao, Gul Habib, Banghui Liu, Jing Chen, Jun He, Peng Zhou, Zheng-Li Shi, Xinwen Chen, Xiaoli Xiong

**Affiliations:** ^1^ State Key Laboratory of Respiratory Disease, Guangdong Provincial Key Laboratory of Stem Cell and Regenerative Medicine, Guangdong-Hong Kong Joint Laboratory for Stem Cell and Regenerative Medicine, Guangzhou Institutes of Biomedicine and Health, Chinese Academy of Sciences, Guangzhou 510530, China.; ^2^ University of Chinese Academy of Sciences, Beijing 100864, China.; ^3^ Graduate School of Guangzhou Medical University, Guangzhou Medical University-Guangzhou Institutes of Biomedicine and Health Joint School of Life Sciences, Guangzhou Medical University, Guangzhou 511436, China.; ^4^ Guangzhou National Laboratory, Guangzhou 510005, China.; ^5^ Key Laboratory of Biological Targeting Diagnosis, Therapy and Rehabilitation of Guangdong Higher Education Institutes, The Fifth Affiliated Hospital of Guangzhou Medical University, Guangzhou 510799, China.

## Abstract

Merbecoviruses from bats, pangolins, and hedgehogs pose significant zoonotic threats, with a limited understanding of receptor binding by their spike (S) proteins. Here, we report cryo-EM structures of GD-BatCoV (BtCoV-422) and SE-PangolinCoV (MjHKU4r-CoV-1) RBDs in complex with human DPP4 (hDPP4). These structures exhibit a substantial offset in their hDPP4 interaction interfaces, revealing a conserved hydrophobic cluster as a convergent signature of DPP4 binding within the MERS-HKU4 clade of merbecoviruses. Structure-guided mutagenesis demonstrates that favorable interactions are distributed across multiple receptor binding motif (RBM) regions, working synergistically to confer high-affinity hDPP4 binding. Swapping of the merbecovirus RBM regions indicate limited plasticity and interchangeability among these regions. In addition, we report cryo-EM structures of six merbecovirus S-trimers. Structure-based phylogenetics suggests that hDPP4-binding merbecoviruses undergo convergent evolution, while ACE2-binding merbecoviruses exhibit diversification in their binding mechanisms. These findings offer critical insights into merbecovirus receptor utilization, providing a structural understanding for future surveillance.

## Teaser

Merbecovirus S-protein structures show convergent evolution for DPP4 binding but divergent evolution for ACE2 binding.

## INTRODUCTION

Pandemic potential of coronaviruses (CoVs) has been demonstrated by the global spread of COVID-19. While most human CoVs can trace their origins in bats (*1*–*6*), zoonosis has been recognized as an important factor in the spillover of CoVs into the human population. Civet was identified as a potential intermediate host for severe acute respiratory syndrome coronavirus (SARS-CoV) human infection (*7*). For SARS-CoV-2 virus, the intermediate host remains elusive, but its capability to infect various animal species likely contributes to its cross-species transmission (*8*). Within the *Betacoronavirus* genus, MERS-CoV (Middle East respiratory syndrome coronavirus), MERS-related CoVs (MERSr-CoVs), HKU4-CoVs, HKU5-CoVs, and Hedgehog-CoVs form the prominent *Merbecovirus* subgenus. MERS-CoV, first identified from patients in Saudi Arabia in 2012 (*9*), is also known to have a strong capability to infect humans, causing 2604 total infections to date with a fatality rate of 36% (*10*). Zoonosis of MERS-CoV was implicated by the isolation of virus strains almost identical to human MERS-CoV from dromedary camels (*11*–*13*). Further full-genome sequence analysis showed that MERS-CoV is closely related to *Tylonycteris* bat CoV HKU4, *Pipistrellus* bat CoV HKU5, and *Neoromicia* bat CoV PML-PHE1/RSA/2011, suggesting that MERS-CoV has a close relationship to bat CoVs (*14*–*19*).

Spike (S) protein is the major viral surface glycoprotein of MERS-CoV and forms homotrimers. Each MERS-CoV S protomer can be divided into two subunits: S1 and S2. S1 consists of N-terminal domain (NTD, 17 to 351), receptor binding domain (RBD, 381 to 587), Domain C (368 to 380, 595 to 653), and Domain D (356 to 367, 654 to 772) (*20*). For the MERS-CoV S-trimer, NTD is known to mediate cell adsorption by binding sialic acid (*21*, *22*), while RBD is known to bind the human dipeptidyl peptidase 4 (hDPP4) as the entry receptor (*23*–*25*). The S2 subunit mediates membrane fusion and exhibits a higher level of sequence conservation among CoVs. A multibasic host protease cleavage site located between the MERS-CoV S1 and S2 is known to modulate cell entry mediated by the S-protein (*26*, *27*). In addition to MERS-CoV, bat CoVs— namely, HKU4 (*17*, *18*), BtCoV/Ii/GD/2014–422 (GD-BatCoV) (*28*), HKU25 (*29*), and a pangolin CoV MjHKU4r-CoV-1 (SE-PangolinCoV) (*30*)—have been shown to bind hDPP4. Although receptors of many merbecoviruses remain unidentified, a few new studies have identified that several MERSr-CoVs and HKU5-CoVs use different bat angiotensin-converting enzyme 2 (bACE2) or human ACE2 (hACE2) molecules as their receptors with distinct receptor binding modes (*31*–*37*). Overall, these findings start to reveal the diversity and complexity of receptor requirements for merbecoviruses.

Here, we report the cryo–electron microscopy (cryo-EM) structures of the GD-BatCoV and SE-PangolinCoV RBDs bound to hDPP4. The GD-BatCoV and SE-PangolinCoV RBDs exhibit weak and strong affinities for hDPP4, respectively. Structure-guided mutagenesis reveals the requirement for high-affinity hDPP4 binding. In addition, S-trimer structures of six merbecoviruses from different animal species were determined. The comparison of the determined merbecovirus RBD structures allows us to identify key determinants of hDPP4 binding, suggesting high receptor diversity within the *Merbecovirus* subgenus.

## RESULTS

### Structures of bat and pangolin merbecovirus RBDs in complex with hDPP4

**Fig. 1.**
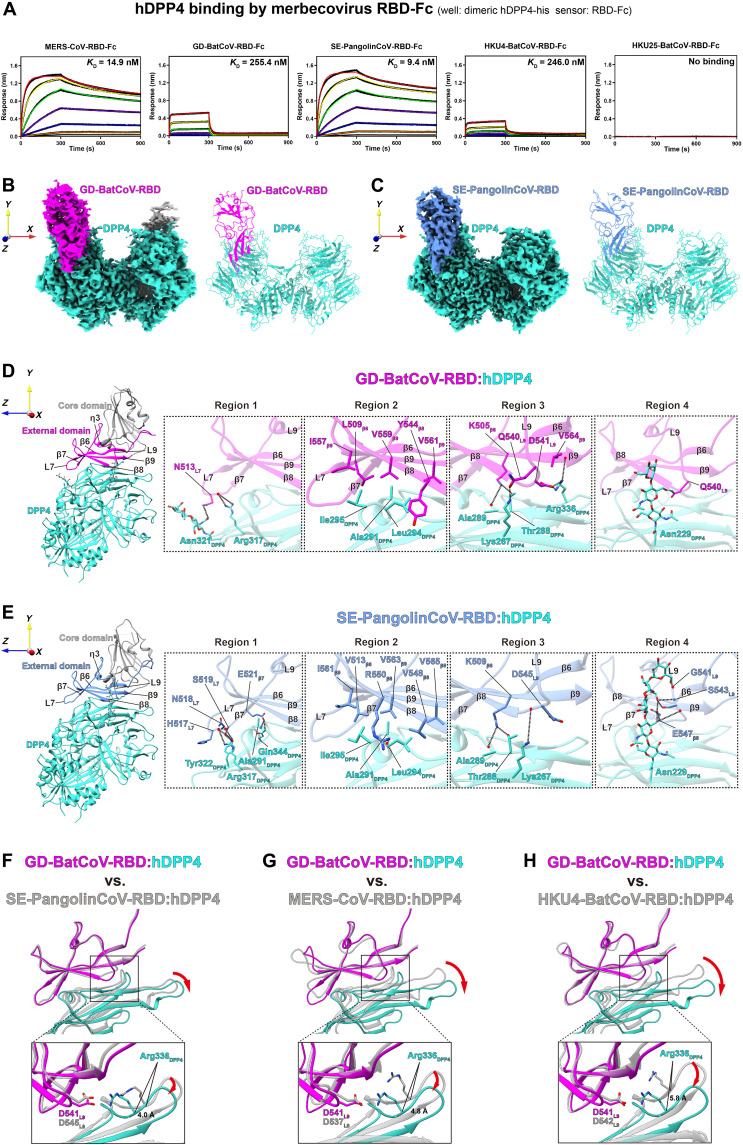
Cryo-EM structures of GD-BatCoV-RBD:hDPP4 and SE-PangolinCoV-RBD:hDPP4 complexes reveal offset hDPP4 binding interfaces. (**A**) Binding assays of RBD-Fc proteins from MERS-CoV, GD-BatCoV, SE-PangolinCoV, HKU4-BatCoV, and HKU25-BatCoV interacting with hDPP4. The RBD-Fc proteins were immobilized on Protein A biosensors and exposed to wells containing hDPP4-his protein with concentrations ranging from 800 to 1.1 nM. The fitted data are shown as colored lines. Equilibrium dissociation constants (*K*_D_) derived from kinetic analyses (table S1) are shown. (**B** and **C**) Cryo-EM density maps and overall structures of GD-BatCoV-RBD:hDPP4 (B) and SE-PangolinCoV-RBD:hDPP4 (C) complexes. The GD-BatCoV-RBD, SE-PangolinCoV-RBD, and hDPP4 are highlighted in magenta, blue, and cyan, respectively. (**D** and **E**) Zoom-in view of the binding interfaces for GD-BatCoV-RBD:hDPP4 (D) and SE-PangolinCoV-RBD:hDPP4 (E) complexes. Secondary structure elements are labeled in the RBM of the two RBDs. The binding interfaces are divided into four regions, and the interaction details are shown, including hydrogen bonds (dashed lines) and hydrophobic interactions (displayed hydrophobic sidechains). (**F** to **H**) Comparison of the GD-BatCoV-RBD:hDPP4 complex with the other available merbecovirus RBD structures in complex with hDPP4. The external domain of GD-BatCoV-RBD serves as the alignment reference. To highlight the offset in hDPP4 binding interface, Arg336_DPP4_ of hDPP4 is shown in stick representation. In addition, D541_GD_ of GD-BatCoV-RBD and corresponding residues (D545_PG_, D537_MERS_, and D542_HKU4_) in SE-PangolinCoV, MERS-CoV, and HKU4-BatCoV RBDs are also shown as sticks. Arg336_DPP4_ in GD-BatCoV-RBD:hDPP4 complex is offset by 4.0 to 5.8 Å relative to its position in the other RBD:hDPP4 complexes.

We selected 11 strains of merbecovirus to validate their binding ability to the receptor hDPP4. These 11 strains are derived from bats, pangolins, and hedgehogs, namely, BtCoV/Ii/GD/2014-422/*Ia io*/Guangdong/2014 (GD-BatCoV, also known as BtCoV-422), HKU4/*Tylonycteris pachypus*/Guangdong/2007 (HKU4-BatCoV), HKU5/*Japanese pipistrelle*/Guangdong/2007 (HKU5-BatCoV), HKU25/*Hypsugo pulveratus*/Guangdong/2014 (HKU25-BatCoV), BtVs-BetaCoV/SC2013/*Vespertilio superans*/Sichuan/2014 (SC-BatCoV), BtCoV/KW2E-F93/Nyc_spec/GHA/2010/*Nycteris* sp./Ghana/2011 (GHA-BatCoV), Neoromicia/PML-PHE1/RSA/2011/*Neoromicia capensis*/South Africa/2011 (SA-BatCoV), Vs-CoV-1/*Vespertilio sinensis*/Japan/2019 (Japan-BatCoV), MjHKU4r-CoV-1/*Manis javanica*/Southeast Asia/2023 (SE-PangolinCoV), HKU31/*Erinaceus amurensis*/China/2014 (CN-HedgehogCoV), and Erinaceus/VMC/DEU/2012/*Erinaceus europaeus*/Germany/2012 (EU-HedgehogCoV) (*14*, *28*–*30*, *38*–*43*). HKU5-BatCoV-S contains a multibasic “RVRR” S1/S2 cleavage site, similar to MERS-CoV-S, whereas all other merbecovirus S-proteins studied have single Arg S1/S2 sites. Amino acid sequence alignment shows that these S-proteins share 52.8 to 70.6% identity with the MERS-CoV S-protein. However, more substantial variations are observed in the RBDs, with identities ranging from 33.2 to 67.5% compared to the MERS-CoV-RBD (fig. S1). Next, we obtained S-trimer or RBD proteins of these strains and assessed their binding to hDPP4 through biolayer interferometry (BLI) using the MERS-CoV S-trimer and RBD proteins as references (Fig. 1A and fig. S2). The BLI results show that MERS-CoV, GD-BatCoV, SE-PangolinCoV, and HKU4-BatCoV are able to bind hDPP4, as previously reported (*17*, *18*, *28*, *30*), albeit with different binding kinetics. For DPP4-binding viruses, stepwise avidity-enhanced receptor binding has been observed with monomeric, dimeric, and trimeric analyte proteins (Fig. 1A and fig. S2), in line with our previous study on sarbecovirus S-proteins (*44*). Because some monomeric RBDs only weakly bind hDPP4, hDPP4 binding is primary compared among dimeric hDPP4-his protein binding to different, immobilized RBD-Fc proteins. This avidity-enhanced approach, as used previously, provides more accurate affinity estimates (*44*). The other tested S-trimer or RBD proteins did not bind hDPP4 (Fig. 1A and fig. S2). Notably, the SE-PangolinCoV-RBD-Fc bound to hDPP4 with an affinity comparable to MERS-CoV, whereas GD-BatCoV and HKU4-BatCoV RBDs, RBD-Fc proteins, and S-trimers exhibited substantially weaker receptor binding affinities (Fig. 1A). Although evidence suggests that HKU25-BatCoV uses hDPP4 as the receptor (*29*), its RBD-Fc and S-trimer failed to show binding to hDPP4 in our BLI assays (Fig. 1A and fig. S2).

To understand hDPP4 binding by GD-BatCoV and SE-PangolinCoV S-proteins, we determined cryo-EM structures of their S-RBDs in complex with hDPP4 at overall resolutions of 3.4 and 2.7 Å, respectively (Fig. 1, B and C, and figs. S3 and S4). Notably, during the preparation of this manuscript, two crystal structures of the SE-PangolinCoV-RBD:hDPP4 complex became available (*45*, *46*). While cryo-EM and x-ray structures are largely consistent, certain perturbations in hDPP4 interactions were observed (see below). In both our RBD:hDPP4 complex structures, only one S-RBD can be observed to bind one monomer of the hDPP4 dimer (Fig. 1, B and C). The RBDs of GD-BatCoV and SE-PangolinCoV consist of two subdomains, in line with the general architecture observed for CoV RBDs. A core subdomain is positioned distally from the interacting hDPP4 receptor, while an external subdomain, also designated as the receptor binding motif (RBM), is dedicated to hDPP4 binding (Fig. 1, D and E, left panels). The external subdomain features a strand-enriched structure with four antiparallel β sheets (β6 to β9) and exposes a flat sheet face for receptor engagement (Fig. 1, D and E, left panels).

### Comparison of hDPP4 binding interfaces among MERS-CoV and bat/pangolin merbecovirus RBD complexes

In the complex structure of GD-BatCoV-RBD:hDPP4, the total buried surface area (BSA) is 1586.9 Å^2^ with 764.4 and 822.5 Å^2^ on RBD and hDPP4, respectively. The SE-PangolinCoV-RBD:hDPP4 complex has a total BSA of 1866.9 Å^2^, with 861.3 Å^2^ on the RBD and 1005.6 Å^2^ on the hDPP4. The BSAs in the GD-BatCoV-RBD:hDPP4 complex are smaller than those in the MERS-CoV-RBD:hDPP4 (*24*, *46*) (total: 1962.5 Å^2^, MERS-RBD: 925.9 Å^2^, hDPP4: 1036.6 Å^2^) and HKU4-BatCoV-RBD:hDPP4 (total: 1692.2 Å^2^, HKU4-RBD: 798.5 Å^2^, hDPP4: 893.7 Å^2^) complexes. The BSAs in the SE-PangolinCoV-RBD:hDPP4 complex are comparable to those in the MERS-CoV-RBD:hDPP4 complex but larger than those in the HKU4-BatCoV-RBD:hDPP4 and GD-BatCoV-RBD:hDPP4 complexes. These results show that tighter hDPP4 binding is generally associated with larger BSAs (Fig. 1A).

To investigate the key residues involved in hDPP4 binding, we examined the RBD:hDPP4 interfaces, which can be divided into four regions (Fig. 1, D and E, right panels and figs. S5 and S6). In region 1, N513_GD_ within loop7 of GD-BatCoV-RBD interacts with Arg317_DPP4_ and the glycan attached to Asn321_DPP4_ in hDPP4, forming three potential hydrogen bonds (Fig. 1D, region 1 and fig. S5A, region 1). Similarly, in the SE-PangolinCoV-RBD:hDPP4 complex, loop7 residues H517_PG_, N518_PG_, and S519_PG_ interact with the side chains of Arg317_DPP4_ and Tyr322_DPP4_ from hDPP4, forming four potential hydrogen bonds (Fig. 1E, region 1 and fig. S5C, region 1). Despite substantial amino acid variation in loop7 between the GD-BatCoV and SE-PangolinCoV RBDs, loop7 residues maintain hydrogen bond interactions to hDPP4 in different ways. In addition, in region 1, E521_PG_ of β7 in SE-PangolinCoV-RBD engages in two hydrogen bonds with Ala291_DPP4_ and Gln344_DPP4_. These hydrogen bonds are maintained in the MERS-CoV-RBD:hDPP4 and HKU4-BatCoV-RBD:hDPP4 complexes but are absent in the GD-BatCoV-RBD:hDPP4 complex due to increased distance (Fig. 1, D and E, region 1 and fig. S5, A to D, region 1). Therefore, SE-PangolinCoV-RBD forms three more hydrogen bonds in region 1 compared with GD-BatCoV-RBD.

In region 2 of the GD-BatCoV-RBD:hDPP4 complex, RBD hydrophobic residues L509_GD_ (β6), Y544_GD_ (β8), I557_GD_ (β9), V559_GD_ (β9), and V561_GD_ (β9) form a hydrophobic cluster with hDPP4 residues Ala291_DPP4_, Leu294_DPP4_, and Ile295_DPP4_ (Fig. 1D, region 2). Similarly, V513_PG_ (β6), V548_PG_ (β8), I561_PG_ (β9), V563_PG_ (β9), and V565_PG_ (β9) of SE-PangolinCoV-RBD form a hydrophobic cluster with the same hydrophobic hDPP4 residues (Fig. 1, D and E, region 2). Notably, despite sequence variations, hydrophobic residues at corresponding positions in the MERS-CoV and HKU4-BatCoV RBDs also interact with the same hydrophobic hDPP4 residues—Ala291_DPP4_, Leu294_DPP4,_ and Ile295_DPP4_ (fig. S5, A to D, region 2). Furthermore, residue R550_PG_ in the SE-PangolinCoV-RBD forms a hydrogen bond with the main chain oxygen of hDPP4 residue Leu294_DPP4_, and the corresponding residue R542_MERS_ in the MERS-CoV-RBD also forms a hydrogen bond with Leu294_DPP4_. In HKU4-CoV-RBD, K547_HKU4_ (corresponding to R542_MERS_) forms a hydrogen bond with Ile295_DPP4_ instead (fig. S5D, region 2). In the GD-BatCoV-RBD, the corresponding residue is changed to threonine (T546_GD_), and as a result, the interaction is not maintained due to the shorter side chain of T546_GD_ (fig. S5A, region 2).

In region 3, K505_GD_ (β6) in GD-BatCoV-RBD and the corresponding K509_PG_ (β6) in SE-PangolinCoV-RBD both interact with Thr288_DPP4_ and Ala289_DPP4_ in hDPP4 (Fig. 1, D and E, region 3). The equivalent interactions are maintained in the MERS-CoV-RBD and HKU4-BatCoV-RBD [fig. S5, B (region 3) and D (region 3)]. hDPP4 residue Lys267_DPP4_ forms distinct interactions with residues in GD-BatCoV-RBD, SE-PangolinCoV-RBD, MERS-CoV-RBD, and HKU4-BatCoV-RBD. Particularly, Lys267_DPP4_ interacts with noncorresponding residues Q540_GD_ (loop9) in GD-BatCoV-RBD and D545_PG_ (loop9) in SE-PangolinCoV-RBD. In the MERS-CoV-RBD:hDPP4 complex, Lys267_DPP4_ interacts with D539_MERS_, which corresponds to D543_GD_ and E547_PG_ (loop9). In the HKU4-BatCoV-RBD:hDPP4 complex, Lys267_DPP4_ interacts with residues E541_HKU4_ and D542_HKU4_ (both in loop9). These residues correspond to Q540_GD_ in GD-BatCoV-RBD and D545_PG_ in SE-PangolinCoV-RBD, which also interact with Lys267_DPP4_ in their respective RBD:hDPP4 complexes (see above). Notably, because of changes at the interface (see below), hDPP4 residue Arg336_DPP4_ interacts with residues from GD-BatCoV-RBD and MERS-CoV-RBD but not in our SE-PangolinCoV-RBD:hDPP4 complex [Arg336_DPP4_ was modeled to interact with SE-PangolinCoV-RBD in recent reports (*45*, *46*) (fig. S7)]. Arg336_DPP4_ interacts with D541_GD_ (loop9) and V564_GD_ (β9) in GD-BatCoV-RBD, while in MERS-CoV-RBD, it interacts with Y499_MERS_ (β6). Arg336_DPP4_ does not interact with the RBM residues of SE-PangolinCoV-RBD or HKU4-BatCoV-RBD. Structural comparison further identifies a unique hydrogen bond in this region between hDPP4 residue Gln286_DPP4_ and N501_MERS_ of MERS-CoV-RBD. Because of amino acid changes, corresponding residues in GD-BatCoV, SE-PangolinCoV, and HKU4-BatCoV RBDs are unable to form hydrogen bonds with Gln286_DPP4_ (fig. S5, A to D, region 3).

In region 4, Q540_GD_ of GD-BatCoV-RBD forms a single hydrogen bond with the glycan linked to hDPP4 residue Asn229_DPP4_ (Fig. 1D, region 4). However, G541_PG_, S543_PG_, and E547_PG_ of SE-PangolinCoV-RBD establish multiple hydrogen bonds with multiple sugars within the Asn229_DPP4_ glycan (Fig. 1E, region 4). In MERS-CoV, hydrophobic sidechain of W535_MERS_ contacts the Asn229_DPP4_ glycan (fig. S5B, region 4), whereas in GD-BatCoV, SE-PangolinCoV, and HKU4-BatCoV, the residues corresponding to W535_MERS_ are serine (fig. S5, A to D, region 4). Therefore, RBDs of the four merbecoviruses interact with the Asn229_DPP4_ glycan differently, reflecting distinct binding interactions among these viruses (fig. S5, A to D, region 4).

### Twisting hDPP4 binding interfaces among RBD:hDPP4 complexes

To further understand the differences in hDPP4 binding, we superposed the RBM from GD-BatCoV-RBD:hDPP4 structure onto the structures of the SE-PangolinCoV-RBD:hDPP4, MERS-CoV-RBD:hDPP4, and HKU4-BatCoV-RBD:hDPP4. The RBMs are well aligned with Cα root mean square deviation (RMSD) values of 0.72, 0.70, and 0.69 Å, respectively, suggesting that RBM structures are similar, as expected. However, after alignment, the RMSDs between hDPP4s are 5.78, 6.50, and 9.13 Å, respectively (Fig. 1, F to H, and fig. S8). We further identified that there are also substantial differences in hDPP4 binding between SE-PangolinCoV-RBD and MERS-CoV-RBD (hDPP4 RMSD = 5.10 Å) and between SE-PangolinCoV-RBD and HKU4-RBD (hDPP4 RMSD = 5.57 Å) (fig. S8, A and B and D). hDPP4 binds more similarly between MERS-CoV-RBD and HKU4-RBD (hDPP4 RMSD = 3.72 Å) (fig. S8, C and D). These analyses demonstrate substantial differences in hDPP4 binding among different merbecovirus RBDs, likely resulting from variations in amino acids at the binding interfaces.

**Fig. 2.**
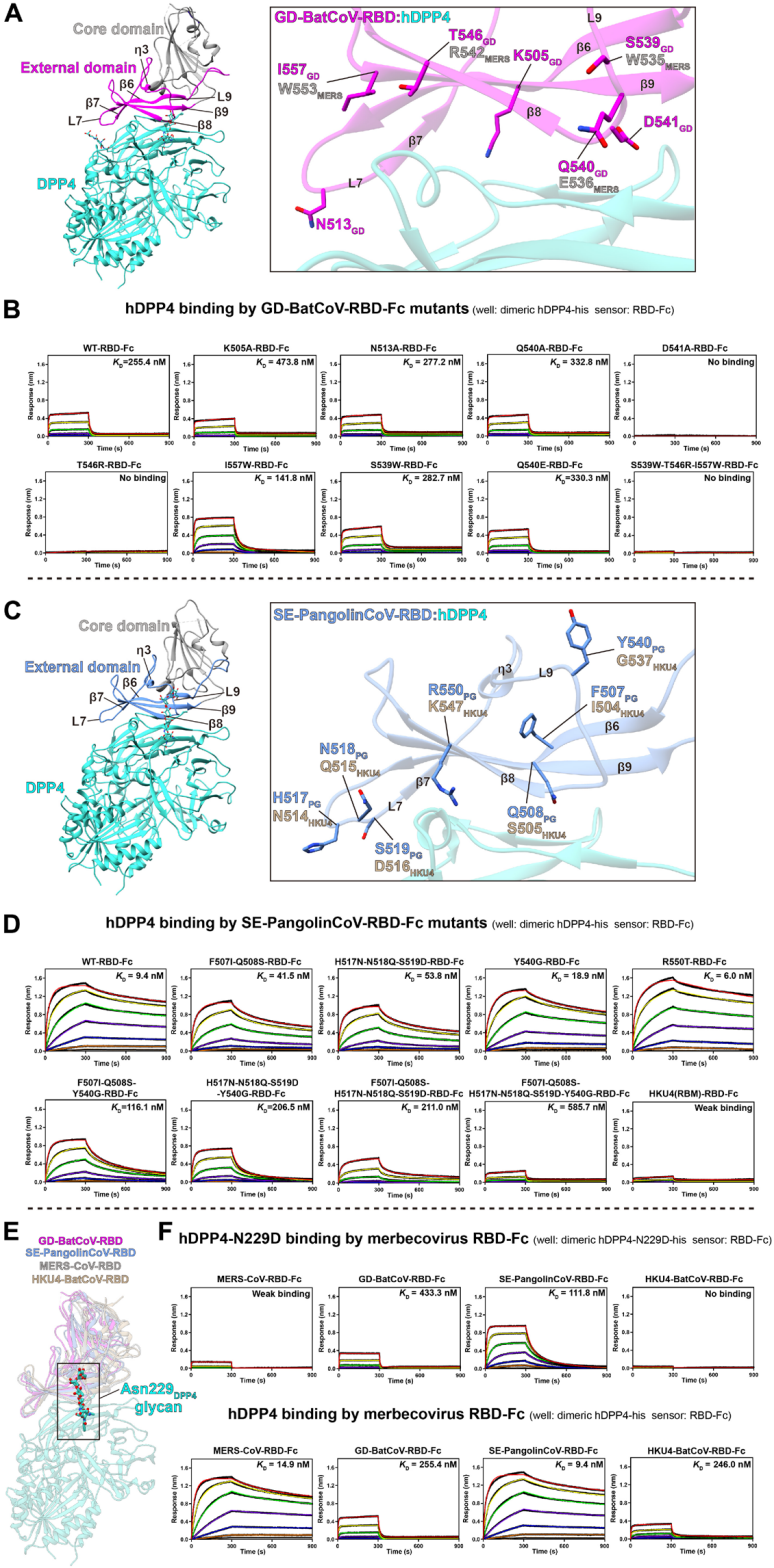
Binding of hDPP4 by GD-BatCoV and SE-PangolinCoV RBD mutants. (**A** and **B**) Mutagenesis of GD-BatCoV-RBD and its effect on hDPP4 binding. (A) Cartoon of GD-BatCoV-RBD showing the locations where mutations were introduced. Gray labels indicate the corresponding amino acids in MERS-CoV-RBD. (B) Sensorgrams of hDPP4 binding by different GD-BatCoV-RBD mutants. (**C** and **D**) Mutagenesis of SE-PangolinCoV-RBD and its effect on hDPP4 binding. (C) Cartoon of SE-PangolinCoV-RBD showing the locations where mutations were introduced. Beige labels indicate the corresponding amino acids in HKU4-BatCoV-RBD. (D) Sensorgrams of hDPP4 binding by different SE-PangolinCoV-RBD mutants. (**E** and **F**) Effect of the hDPP4-N229D mutant on the binding of MERS-CoV-RBD-Fc, GD-BatCoV-RBD-Fc, SE-PangolinCoV-RBD-Fc, and HKU4-BatCoV-RBD-Fc. (E) Detailed view of the Asn229_DPP4_ glycan binding interface across hDPP4 complex with MERS-CoV, GD-BatCoV, SE-PangolinCoV, and HKU4-BatCoV RBDs. (F) Sensorgrams showing binding of the mutant hDPP4-N229D-his protein by MERS-CoV, GD-BatCoV, SE-PangolinCoV, and HKU4-BatCoV RBDs. For comparison, binding assays using WT hDPP4-his protein are also shown. In BLI assays, hDPP4-his or hDPP4-N229D-his protein was formulated in wells as a threefold dilution series with concentrations ranging from 800 to 1.1 nM. *K*_D_ values are shown alongside the binding curves. Binding kinetic parameters are summarized in tables S3 to S5.

Therefore, the interface in the GD-BatCoV-RBD:hDPP4 complex is substantially twisted by comparison with other RBD:hDPP4 complexes (Fig. 1, F to H). We further found that the interaction between D541_GD_ and Arg336_DPP4_ in region 3 could be a major determinant for the twisted hDPP4 binding by the GD-BatCoV-RBD. Arg336_DPP4_ from the GD-BatCoV-RBD:hDPP4 complex is offset by 4.0 to 5.8 Å compared to other RBD:hDPP4 complexes (Fig. 1, F to H). In addition, the side chain of Arg336_DPP4_ adopts different orientations across the four RBD:hDPP4 complexes. Although D541_GD_ is conserved in SE-PangolinCoV, MERS-CoV, and HKU4-BatCoV, only D541 from GD-BatCoV-RBD forms a salt bridge with Arg336_DPP4_, bringing the Arg336_DPP4_ loop closer to GD-BatCoV-RBD, thus leading to the observed deviation in hDPP4 binding by the GD-BatCoV-RBD. Experimental validation further confirms the significance of this interaction for hDPP4 binding (see below, Fig. 2B, top). Comparison of the four RBD:hDPP4 complex structures identifies that the interactions in region 1 (loop7) and region 2 (hydrophobic core) with hDPP4 are highly conserved despite residue variations, while the interactions of region 3 with hDPP4 are the most divergent. Collectively, these interface differences likely result in substantial variations in hDPP4 binding and modulation of hDPP4 affinity.

### Point mutations in GD-BatCoV-RBD have limited effect on hDPP4 binding

Given that GD-BatCoV-RBD interacts distinctly with hDPP4 compared to MERS-CoV, SE-PangolinCoV, and HKU4-BatCoV, we initially mutated the amino acids involved in polar interactions in GD-BatCoV-RBD to alanine (K505A_GD_, N513A_GD_, Q540A_GD_, and D541A_GD_; see Figs. 1D and 2A for locations) to assess their impact on hDPP4 binding. Whereas hDPP4 binding by GD-BatCoV-RBD K505A_GD_, N513A_GD_, and Q540A_GD_ mutants remained largely unchanged compared to the wild type (WT) (Fig. 2B, top), the D541A_GD_ mutant, which disrupts an interaction with Arg336_DPP4_, completely abolished hDPP4 binding (Fig. 2B, top), highlighting its critical role in GD-BatCoV-RBD for binding hDPP4.

hDPP4-interacting amino acids or their combinations from MERS-CoV-RBD were introduced into GD-BatCoV-RBD to test whether they enhance hDPP4 binding (see Fig. 2A and fig. S9 for locations of the introduced mutations). In region 2, residue T546_GD_ was substituted with R542_MERS_, which interacts with the main chain of Leu294_DPP4_ in MERS-CoV-RBD. Unexpectedly, although an arginine is found in the other hDPP4-binding RBDs, the T546R_GD_ mutation in GD-BatCoV-RBD is incompatible with hDPP4 binding, completely abolishing it. Residue I557_GD_, corresponding to W553_MERS_ in β9, contacts hDPP4 through hydrophobic interactions. When mutated to I557W_GD_ in GD-BatCoV-RBD, hDPP4 binding is modestly increased (Fig. 2B, bottom). These results indicate that the hydrophobic cluster in region 2 of bat/pangolin merbecovirus RBD:hDPP4 interface can affect hDPP4 binding.

In region 4, residue S539_GD_ corresponds to W535_MERS,_ which interacts with Asn229_DPP4_-linked glycan (see Fig. 2A and fig. S9 for locations of the introduced mutations). The S539W_GD_ mutation in GD-BatCoV-RBD showed minimal effect on hDPP4 binding. Structural analysis suggests that Q540_GD_ corresponds to E536_MERS_, and Q540E_GD_ should potentially introduce a salt bridge to interact with Lys267_DPP4_. However, the Q540E_GD_ mutation did not result in a substantial enhancement in binding between GD-BatCoV-RBD and hDPP4, possibly due to the repulsion from the nearby negative-charged D543_GD_, preventing the introduced Q540E_GD_ from interacting effectively [Fig. 2B and figs. S5A (region 3) and S9]. Further testing found that S539W_GD_ + T546R_GD_ + I557W_GD_ (combination of the three above tested mutations based on MERS-CoV sequence) negatively affected hDPP4 binding when introduced into GD-BatCoV-RBD despite a previous report showing that an equivalent mutation combination in HKU4-BatCoV substantially enhances hDPP4 binding (~85-fold increase) (Fig. 2B) (*17*).

### Determinants in SE-PangolinCoV-RBD for high hDPP4 affinity

SE-PangolinCoV is closely related to HKU4-BatCoV based on S-protein and full-genome phylogenetics (*30*). The SE-PangolinCoV S-protein exhibits much higher affinity for hDPP4 than the HKU4-BatCoV S-protein. To identify residues that confer hDPP4 affinity, SE-PangolinCoV-RBD-Fc mutants were constructed, mutating residues to those in HKU4-BatCoV-RBD. The sequence alignment showed that SE-PangolinCoV-RBD differs from HKU4-BatCoV-RBD in four sequence stretches located within or proximal to the hDPP4-binding interface (Fig. 2C and fig. S9). In β6, F507_PG_ and Q508_PG_ are found in SE-PangolinCoV-RBD, corresponding to I504_HKU4_ and S505_HKU4_ in HKU4-BatCoV-RBD (Fig. 2C). The F507I_PG_ + Q508S_PG_ double mutant in SE-PangolinCoV shows a ~4.4-fold reduction in hDPP4 binding (Fig. 2D). In loop7, SE-PangolinCoV-RBD contains H517_PG_, N518_PG_, and S519_PG_, corresponding to N514_HKU4_, Q515_HKU4_, and D516_HKU4_ (Fig. 2C and fig. S9). H517N_PG_ + N518Q_PG_ + S519D_PG_ triple mutant results in a ~5.7-fold decrease in SE-PangolinCoV hDPP4 binding (Fig. 2D). Additional differences between SE-PangolinCoV-RBD and HKU4-BatCoV-RBD occur in loop9 and β8. In loop9, Y540_PG_ is found in SE-PangolinCoV-RBD, corresponding to G537_HKU4_ in HKU4-BatCoV-RBD. The Y540G_PG_ single mutation reduces hDPP4 binding by ~2-fold (Fig. 2D). In β8, R550_PG_ in SE-PangolinCoV-RBD corresponds to K547_HKU4_, but the R550K_PG_ mutant failed to express. Nevertheless, we tested an R550T_PG_ mutant, introducing a threonine found in GD-BatCoV-RBD at the equivalent position (T546_GD_). R550T_PG_ mutation in β8 has minimal effect on hDPP4 binding (Fig. 2D).

Notably, when the β6 F507I_PG_ + Q508S_PG_ double mutation is combined with the loop9 Y540G_PG_ mutation, the F507I_PG_ + Q508S_PG_ + Y540G_PG_ mutant shows a 12.4-fold decrease in hDPP4 binding. Combining loop7 H517N_PG_ + N518Q_PG_ + S519D_PG_ triple mutation with the loop9 Y540G_PG_ mutation results in a 22.0-fold decrease in hDPP4 binding (Fig. 2D). When β6 F507I_PG_ + Q508S_PG_ and loop7 H517N_PG_ + N518Q_PG_ + S519D_PG_ mutations are combined, the F507I_PG_ + Q508S_PG_ + H517N_PG_ + N518Q_PG_ + S519D_PG_ mutant exhibits a 22.4-fold decrease in hDPP4 binding (Fig. 2D). Further, when the F507I_PG_ + Q508S_PG_ + H517N_PG_ + N518Q_PG_ + S519D_PG_ mutant is combined with Y540G_PG_, the F507I_PG_ + Q508S_PG_ + H517N_PG_ + N518Q_PG_ + S519D_PG_ + Y540G_PG_ sextuple mutant shows a 62-fold reduction in hDPP4 binding, decreasing to 585.7 nM compared to 9.4 nM for the WT SE-PangolinCoV-RBD (Fig. 2D). These results indicate that multiple hDPP4 contact sites contribute to high-affinity binding of SE-PangolinCoV-RBD to hDPP4.

Given the extensive interactions between SE-PangolinCoV-RBD residues and the hDPP4 Asn229-glycan, we tested the effect of removing the Asn229-glycan with the N229D_DPP4_ mutation. MERS-CoV-RBD and HKU4-BatCoV-RBD almost completely and completely lost binding to the N229D_DPP4_ mutant, respectively (Fig. 2, E and F). In contrast, N229D_DPP4_ has a weaker effect for GD-BatCoV-RBD (433.3 nM versus 255.4 nM) and SE-PanglinCoV-RBD (111.8 nM versus 9.4 nM). Possibly due to variations in binding mode, merbecovirus RBDs rely differently on Asn229_DPP4_-glycan for hDPP4 binding.

### Low plasticity of GD-BatCoV-RBD requires replacement of external RBM domain for high-affinity hDPP4 binding

Point mutations have limited effects on hDPP4 binding in GD-BatCoV-RBD. To investigate the contributions of specific RBM regions on hDPP4 binding, fragment replacements were performed within the RBM regions between GD-BatCoV and MERS-CoV. For GD-BatCoV-RBD, replacing most regions within the RBM resulted in a reduction or complete loss of hDPP4 binding (fig. S10A). The fragment replacement of MERS-CoV-RBD by GD-BatCoV-RBD sequences substantially reduced hDPP4 binding or resulted in complete loss of hDPP4 binding (fig. S10B). These results suggest low plasticity of merbecovirus RBM regions.

**Fig. 3.**
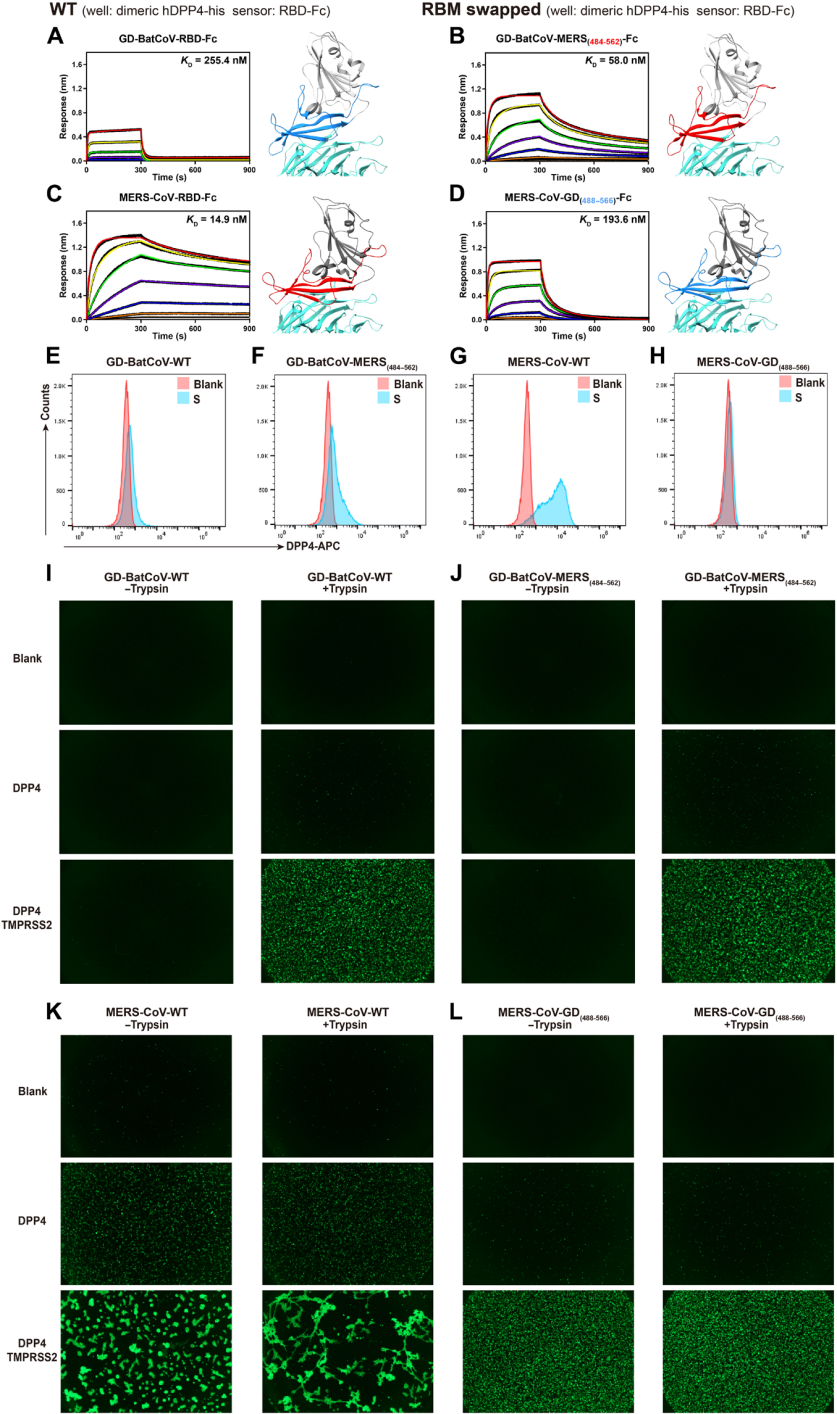
External domain swaps between GD-BatCoV-RBD and MERS-CoV-RBD and their effects on hDPP4 binding and cell-cell fusion activities. (**A** to **D**) hDPP4 binding by WT and external domain-swapped mutants of GD-BatCoV-RBD-Fc and MERS-CoV-RBD-Fc. In BLI assays, hDPP4-his was formulated in wells as a threefold dilution series from 800 to 1.1 nM. *K*_D_ values are shown alongside the binding curves. Binding kinetic parameters are summarized in table S6. (**E** to **H**) Flow cytometric (FCM) analysis of hDPP4 binding by WT GD-BatCoV-S and MERS-CoV-S and their RBM-swapped mutants. 293T cells transiently expressing S-proteins were mixed with hDPP4-Fc protein. FCM was performed using an Allophycocyanin (APC)-conjugated goat anti-human IgG Fc probe. (**I** to **L**) Receptor binding mediated cell-cell fusion activities of WT GD-BatCoV-S and MERS-CoV-S and their RBM-swapped mutants. 293T cells expressing S-proteins served as the effector cells, while 293T cells expressing hDPP4 or hDPP4 + TMPRSS2 were used as the receptor cells. To test the effect of trypsin, the effector cells were incubated with trypsin (5 μg/ml) at 37°C for 10 min before mixing with receptor cells.

Expected changes in hDPP4 binding were observed only when the entire RBMs of GD-BatCoV-RBD and MERS-CoV-RBD were replaced. Replacing GD-BatCoV-RBM with MERS-CoV-RBM resulted in a ~4.4-fold enhancement in hDPP4 binding, reaching an equilibrium dissociation constant (*K*_D_) of 58 nM (Fig. 3, A and B). When MERS-CoV-RBM was replaced with GD-BatCoV-RBM sequence (488-566_GD_), the MERS-GD_(488–566)_-RBD exhibited a ~13-fold reduction in hDPP4 binding, with a *K*_D_ of 194 nM. MERS-GD_(488–566)_-RBD has a tighter hDPP4 *K*_D_ than the native GD-BatCoV-RBD (255 nM), suggesting that regions outside of RBM contribute to hDPP4 affinity (Fig. 3, A to D). Affinity changes were further confirmed by flow cytometry (Fig. 3, E to H).

Subsequently, fusion activities for these S-proteins were tested in cell-cell fusion assays. Neither the WT GD-BatCoV-S-protein nor the GD-MERS_(484–562)_-S-protein expressing effector cells demonstrated fusion activity when mixed with hDPP4-expressing receptor cells. A slight increase in fusion was observed when trypsin was added (Fig. 3, I and J, middle panels). Substantial fusion activity was observed only when receptor cells expressed both hDPP4 and TMPRSS2 and with added trypsin (Fig. 3, I and J, bottom panels). Effector cells expressing WT MERS-CoV-S-protein exhibited substantially stronger fusion activity than the cells expressing WT GD-BatCoV-S-protein (Fig. 3, I and K). Substantial fusion was observed between effector cells expressing WT MERS-CoV-S-protein and receptor cells expressing hDPP4 alone, with TMPRSS2 expression further enhancing WT MERS-CoV-S-protein fusion (Fig. 3, K and L). In contrast, substantial fusion was only observed for effector cells expressing chimeric GD-MERS_(484–562)_-S-protein when receptor cells expressed both hDPP4 and TMPRSS2, and in the presence of trypsin (Fig. 3J, bottom). These findings demonstrate that RBM changes altering receptor binding can also modulate S-protein protease sensitivity, substantially affecting fusion activity mediated by S-protein.

### Merbecovirus S-trimers adopt two locked conformations showing ability to bind lipids

**Fig. 4.**
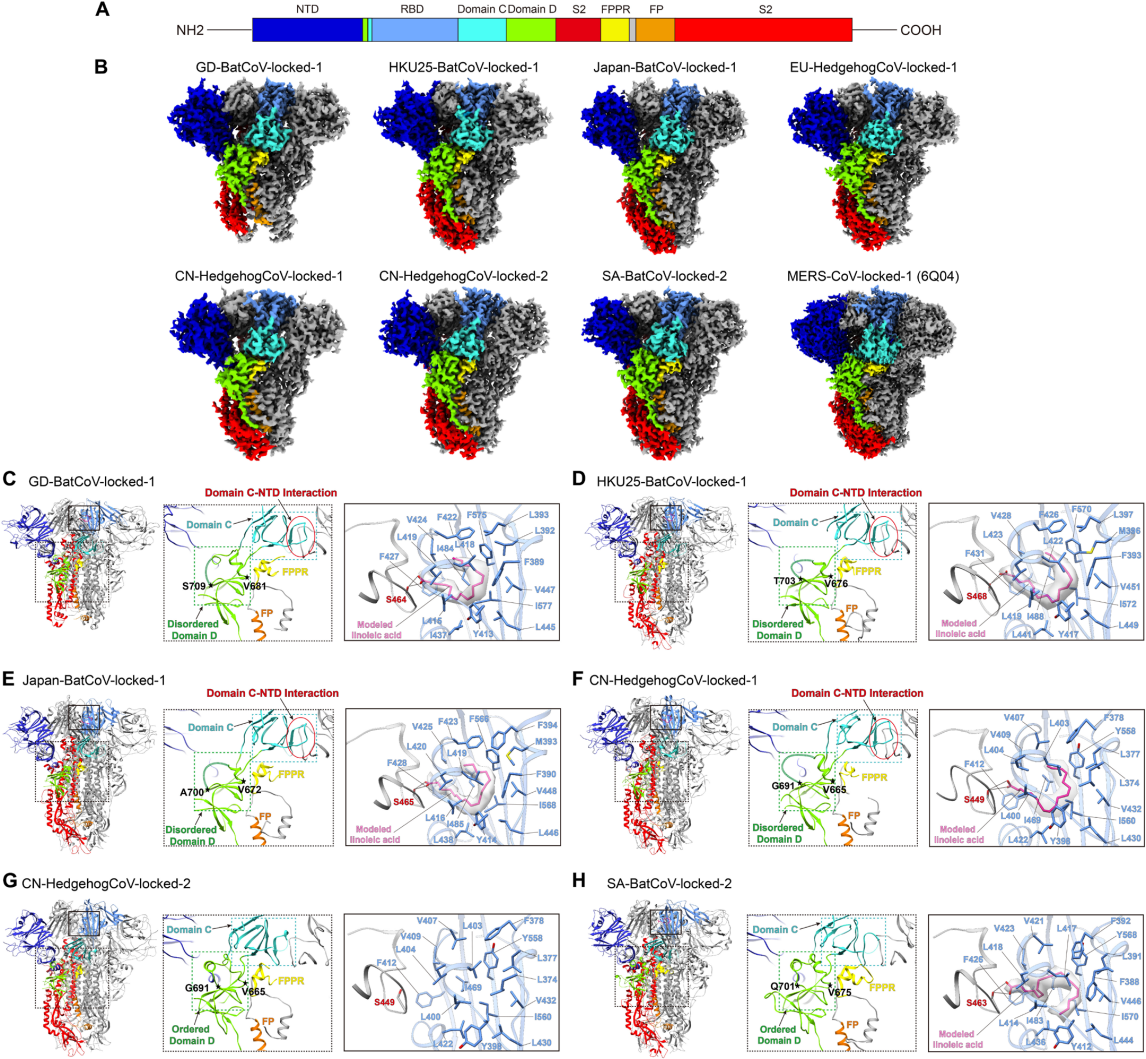
Cryo-EM structures of merbecovirus S-trimers and their features. (**A**) Schematic illustrating the domain organization of the merbecovirus S-protein. Domain boundaries are summarized in table S7. S1 domains, namely, NTD (17-351_MERS_), RBD (381-587_MERS_), Domain C (368-380_MERS_, 595-653_MERS_), and Domain D (356-367_MERS_, 654-772_MERS_) are colored medium blue, light-blue, cyan, and green, respectively. S2 is highlighted in red, with its structural elements, fusion peptide proximal region (FPPR, 905-929_MERS_) and fusion peptide (FP, 941-983_MERS_) colored yellow and orange, respectively. (**B**) Cryo-EM densities showing the overall architectures of the S-trimers of GD-BatCoV, HKU25-BatCoV, Japan-BatCoV, CN-HedgehogCoV, EU-HedgehogCoV, and SA-BatCoV. A featured protomer from each S-trimer is shown and colored according to the scheme in (A). (**C** to **H**) Left panels show molecular models of merbecovirus S-trimers. Middle panels show zoom-in views of Domains C and D with dashed boxes highlighting their positions; red circles highlight interactions between Domain C and the adjacent NTD. Right panels show fatty acid–binding pockets within the merbecovirus RBDs. Hydrophobic amino acid side chains lining the fatty acid–binding pockets are shown as sticks. Structural elements from a neighboring RBD that form the fatty acid–binding pocket are shown in gray, with fatty acid–interacting residues labeled in red.

To further characterize structural features of merbecovirus S-proteins, we determined cryo-EM structures of GD-BatCoV, HKU25-BatCoV, Japan-BatCoV, SA-BatCoV, CN-HedgehogCoV, and EU-HedgehogCoV S-trimers, with resolutions ranging from 2.8 to 3.8 Å (figs. S3A and S4C and S11 to S13). These S-trimer structures were classified into two distinct conformations, designated as locked-1 and locked-2 (see below for structural details), each with all three RBDs in “down” positions (Fig. 4B and fig. S14). The S-trimers of GD-BatCoV, HKU25-BatCoV, Japan-BatCoV, and EU-HedgehogCoV only adopt locked-1 conformations (Fig. 4, C to E, and figs. S14, B to E and S15A), while the SA-BatCoV S-trimer adopts only the locked-2 conformation (Fig. 4H and fig. S14G). GD-BatCoV S-trimer in locked-1 conformation bound to JC57-1, a cross-reactive MERS-CoV antibody, has also been reported (*47*). Notably, the S-trimer of CN-HedgehogCoV was identified to adopt both locked-1 and locked-2 conformations (Fig. 4, F and G). The protomers in each of the S-trimer structures are divided into five typical domains of a coronavirus S-protein: NTD, RBD, Domain C, Domain D, and S2 (Fig. 4, A and B).

Different from previous MERS-CoV and MERSr-CoV PDF-2180 (*31*) S-trimer structures, densities that can be modeled as linoleic acids (LA) were identified in RBDs of most of the currently reported S-trimer structures, with CN-HedgehogCoV-locked-2 S-trimer being the only exception. Binding of LA between RBDs in SARS-CoV and SARS-CoV-2 S-trimers has been proposed to stabilize more tightly packed S-trimer structures previously designated as locked conformations (*20*, *48*). Consequently, the identified fatty acid–bound 3-RBD “down” conformations for the merbecovirus S-trimers are also designated as locked conformations. In the characterized merbecovirus S-trimer structures, the putative LA carboxylate group interacts with a serine from a neighboring RBD via hydrogen bonds. This serine is highly conserved across all the merbecovirus S-proteins [Fig. 4, C to H (right panels), and fig. S15, A and B (right panels)]. For comparison, the LA carboxylate group forms salt bridges with a conserved arginine (R408_SARS2_ in SARS-CoV-2) from a neighboring RBD in sarbecovirus S-proteins (*20*, *48*–*50*). All locked-1 merbecovirus S-trimers we determined are found to bind fatty acids in their RBDs, while fatty acids were also identified within RBDs of locked-2 SA-BatCoV S-trimer (Fig. 4H, right). Although the locked-2 S-trimers of CN-HedgehogCoV and SA-BatCoV are similar in overall S conformation, no fatty acid was identified bound in the RBDs of the locked-2 CN-HedgehogCoV S-trimer (Fig. 4G, right). A comparison revealed that the absence of the fatty acid causes the hydrophobic helix (residues 400 to 406) to shift inward by ~2.4 Å, leading to the contraction of the fatty acid–binding pocket (fig. S15C). Without RBD-bound fatty acids, the contraction of the fatty acid–binding pockets was also observed in MERS-CoV and PDF-2180 (*31*) S-trimers (fig. S15, D to F). In the locked-2 SA-BatCoV S-trimer, the modeled LAs were bound in a different conformation (Fig. 4H).

### Structural transition between the two locked merbecovirus S-trimer conformations

**Fig. 5.**
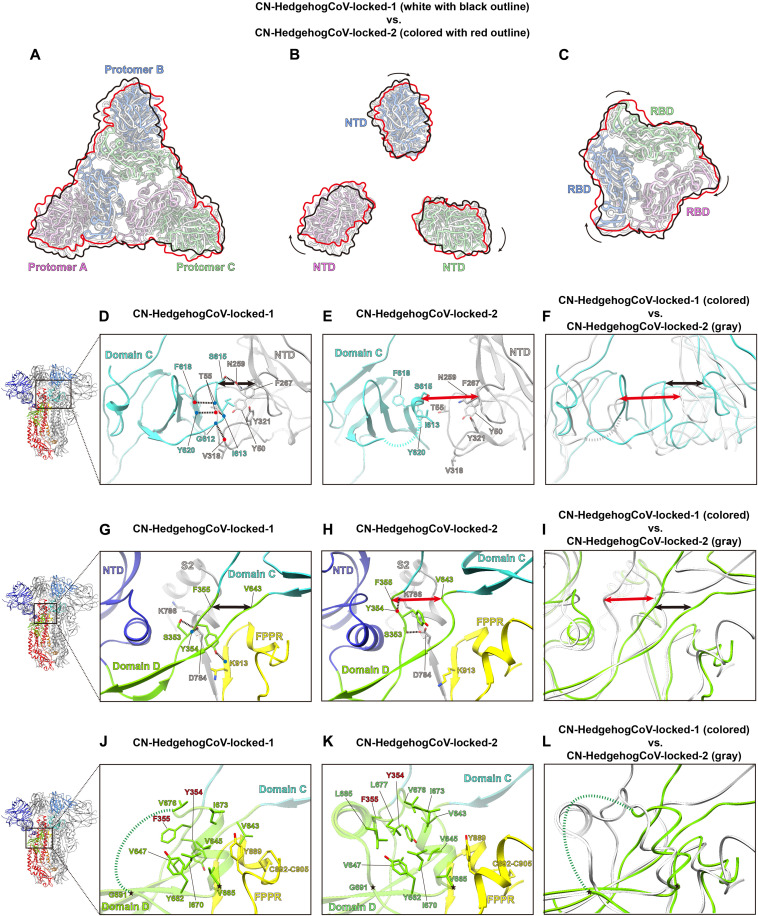
Structural rearrangement of the S-trimer between locked-1 and locked-2 conformations in the CN-HedgehogCoV S-trimer. (**A** to **C**) Top views of the structural overlay of the locked-1 (white with black outline) and locked-2 (colored with red outline) CN-HedgehogCoV S-timers. The overlay shows a clockwise rotation in S1 (A), NTD (B), and RBD (C) between the two locked conformations. (**D** to **F**) Locked-1 and locked-2 CN-HedgehogCoV S-trimers differ at interactions between Domain C and NTD of a neighboring S-protomer. (D) Interactions between Domain C and NTD in the locked-1 CN-HedgehogCoV S-trimer. (E) Interactions between Domain C and NTD in the locked-2 CN-HedgehogCoV S-trimer. Hydrogen bonds are shown as dashed black lines. Disordered regions are represented by dashed lines. (F) Superposition of locked-1 (colored) and locked-2 (gray) CN-HedgehogCoV S-trimers showing structural rearrangement in the Domain C-NTD interaction region. Black and red lines indicate the distances between S615 and N259 in locked-1 and locked-2 structures, respectively. (**G** to **I**) Structural rearrangement of the Domain C-D linker region between the locked-1 and locked-2 CN-HedgehogCoV S-trimers leads to varied interactions between this region and S2. Black and red lines indicate the distances between F355 and V643 in locked-1 and locked-2 structures, respectively. (**J** to **L**) Structural rearrangement of the Domain D loop between the locked-1 and locked-2 CN-HedgehogCoV S-trimers leads to varied Domain D hydrophobic core structures.

A clockwise rotation of the S1 domains was observed during the transition from the locked-1 to locked-2 conformation (Fig. 5, A to C). This change in S-trimer quaternary structure appears to primarily originate from the structural changes in Domain C and Domain D, which affect interactions between protomers (Fig. 5, D to L). In the CN-HedgehogCoV-locked-1 S-trimer, the Domain C residue I613_CN_ forms hydrophobic interactions with NTD residues Y50_CN_, F267_CN_, and Y321_CN_ of an adjacent protomer. These hydrophobic interactions are stabilized by a hydrogen bond network involving Domain C residues G612_CN_, I613_CN_, S615_CN_, F618_CN_, and Y620_CN_, and T55_CN_, N259_CN_, and V318_CN_ of the NTD from the adjacent protomer. However, such interactions between Domain C and the NTD are absent in the CN-HedgehogCoV locked-2 S trimer (Fig. 5, D to F). In addition, the interactions between Domain D and S2 of the adjacent protomer are altered: In the locked-1 S-trimer, Domain D residue Y354_CN_ interacts with S2 residues D784_CN_ and K913_CN_ (Fig. 5G), while in the locked-2 S-trimer, Domain D residue Y354_CN_ interacts with S2 residue K786_CN_ (Fig. 5H). Despite structural changes, the interaction between Domain D residue S353_CN_ and S2 residue D784_CN_ is maintained in both the locked-1 and locked-2 structures (Fig. 5, G to I).

Notable changes are also observed in the structure of Domain D during the transition from the locked-1 to locked-2 conformation in the CN-HedgehogCoV S-trimer. In the locked-1 CN-HedgehogCoV S-trimer, no density was observed for residues 677 to 690, suggesting that these residues form a flexible loop (Fig. 5J). The same region refolds and rigidifies in the locked-2 conformation (Fig. 5K). Further analysis reveals that, transition from the locked-1 to locked-2 conformation, residues Y354_CN_ and F355_CN_ at the junction of Domains C and D reorient (Fig. 5, J and K). In the locked-1 structure, the hydrophobic core is centered around F355_CN_, whereas in the locked-2 structure, it is centered around Y354_CN_, resulting in structural changes in Domain C. A similar reorientation of Domain C/D junction hydrophobic residues was observed for SARS-CoV and SARS-CoV-2 S-trimers transitioning from locked-1 to locked-2 conformation (*48*).

The Domain C and D structures in the GD-BatCoV-locked-1, Japan-BatCoV-locked-1, and EU-HedgehogCoV-locked-1 S-trimers are similar to those in CN-HedgehogCoV-locked-1 S-trimer [Fig. 4, C and E (middle panels) and figs. S15A (middle panel) and S16 to S18]. In addition, available MERS-CoV S-trimer structures also adopt a locked-1 conformation (figs. S15B, S16F, and S17F). In these structures, residues F370_GD_, F371_JPN_, F354_EU_, and F366_MERS_ correspond to F355_CN_ in CN-HedgehogCoV (fig. S17, B and D to F), forming the center of the Domain D hydrophobic core. The density at the junction of Domains C and D in the HKU25-BatCoV-locked-1 structure is unresolved, but based on the Domain C structure and interactions with the adjacent NTD, it is identified as a locked-1 conformation (figs. S16C and S17C). The structures of Domains C and D in SA-BatCoV-locked-2 conformation are similar to those of CN-HedgehogCoV-locked-2, with the Domain D hydrophobic pocket mediated by residue Y368_SA_, which corresponds to Y354_CN_ in CN-HedgehogCoV (fig. S17A). These results indicate that, in merbecoviruses, different interactions between Domain C and Domain D can stabilize different S-trimers conformations.

### Binding of small molecules in the NTD of merbecoviruses

The sequence alignment of merbecovirus NTDs identifies six highly variable loop regions, namely, Loop-N1 (AA 36-40_MERS_), Loop-N2 (AA 89-100_MERS_), Loop-N3 (AA 131-139_MERS_), Loop-N4 (AA 156-160_MERS_), Loop-N5 (AA 209-222_MERS_), and Loop-N6 (AA 301-311_MERS_), showing amino acid insertions and deletions (fig. S19). Sialic acid has been identified as an attachment factor for MERS-CoV infection. In MERS-CoV S-NTD, residues in and around Loop-N1, Loop-N2, Loop-N3, and Loop-N6 form a pocket to bind sialic acid, involving a total of 10 sialic acid contacting residues (Q36_MERS_, F39_MERS_, H91_MERS_, A92_MERS_, F101_MERS_, I131_MERS_, I132_MERS_, S133_MERS_, Q304_MERS_, and R307_MERS_) (figs. S19B and S20) (*21*, *22*). Sequence variations in these loops lead to conservation of 8 of 10 sialic acid contacting residues in HKU25-BatCoV (Q36_HKU25_, H91_HKU25_, A92_HKU25_, F108_HKU25_, I138_HKU25_, I139_HKU25_, S140_HKU25_, and R315_HKU25_), 7 of 10 in GD-BatCoV (F42_GD_, H94_GD_, F106_GD_, I136_GD_, I137_GD_, S138_GD_, and R311_GD_) and Japan-BatCoV (Q36_JPN_, H91_JPN_, A92_JPN_, F107_JPN_, I138_JPN_, and S139_JPN,_ R312_JPN_); 6 of 10 in EU-HedgehogCoV (Q30_EU_, H85_EU_, F90_EU_, I120_EU_, I121_EU_, and S122_EU_); 5 of 10 in SA-BatCoV (Q36_SA_, F40_SA_, F104_SA_, I134_SA_, and R310_SA_), and only 3 of 10 in CN-HedgehogCoV (Q26_CN_, H83_CN_, and F94_CN_) (fig. S20). The mutagenesis of F39_MERS_, H91_MERS_, S133_MERS_, and R307_MERS_—located within Loop-N1, Loop-N2, Loop-N3, and Loop-N6, respectively—impairs MERS-CoV pseudovirus entry by disrupting sialic acid binding (*21*). All of these residues are conserved in GD-BatCoV, three in HKU25-BatCoV and Japan-BatCoV, two in EU-HedgehogCoV and SA-BatCoV, and one in CN-HedgehogCoV. Overall, based on structural and sequence analyses, the sialic acid–binding pockets in GD-BatCoV, HKU25-BatCoV, and Japan-BatCoV show considerable conservation compared to MERS-CoV. However, Loop-N2 in these S-proteins is incompletely resolved, suggesting that further investigation is needed to confirm their sialic acid–binding capacity. In contrast, S-proteins of CN-HedgehogCoV, EU-HedgehogCoV, and SA-BatCoV are unlikely to bind sialic acid due to changes in key sialic acid–binding residues.

Folic acid has also been found to bind to the NTD of MERS-CoV, although its function remains unknown (*51*). In the S-trimer structures we determined, folic acid densities were identified in equivalent sites of CN-HedgehogCoV and EU-HedgehogCoV S-proteins (fig. S21A). Studies have shown that biliverdin binds to the NTDs of SARS-CoV and SARS-CoV-2 and is associated with antibody escape (*20*, *48*, *52*, *53*). In most of the determined merbecovirus S-trimer structures, we found a clear and distinct density bound to an NTD pocket, resembling the biliverdin binding site in the SARS-CoV-2 S-protein (fig. S21, B and C). In the GD-BatCoV S-protomers, the binding pocket for this unidentified small-molecule consists of hydrophobic residues (I125_GD_, Y148_GD_, P149_GD_, F151_GD_, L175_GD_, L184_GD_, I249_GD_, F258_GD_, and F285_GD_) and charged residues (R273_GD_ and K274_GD_). Further investigation is required to elucidate the identity and function of this small molecule.

### Variations in merbecovirus RBD structures and their implications on hDPP4 binding

**Fig. 6.**
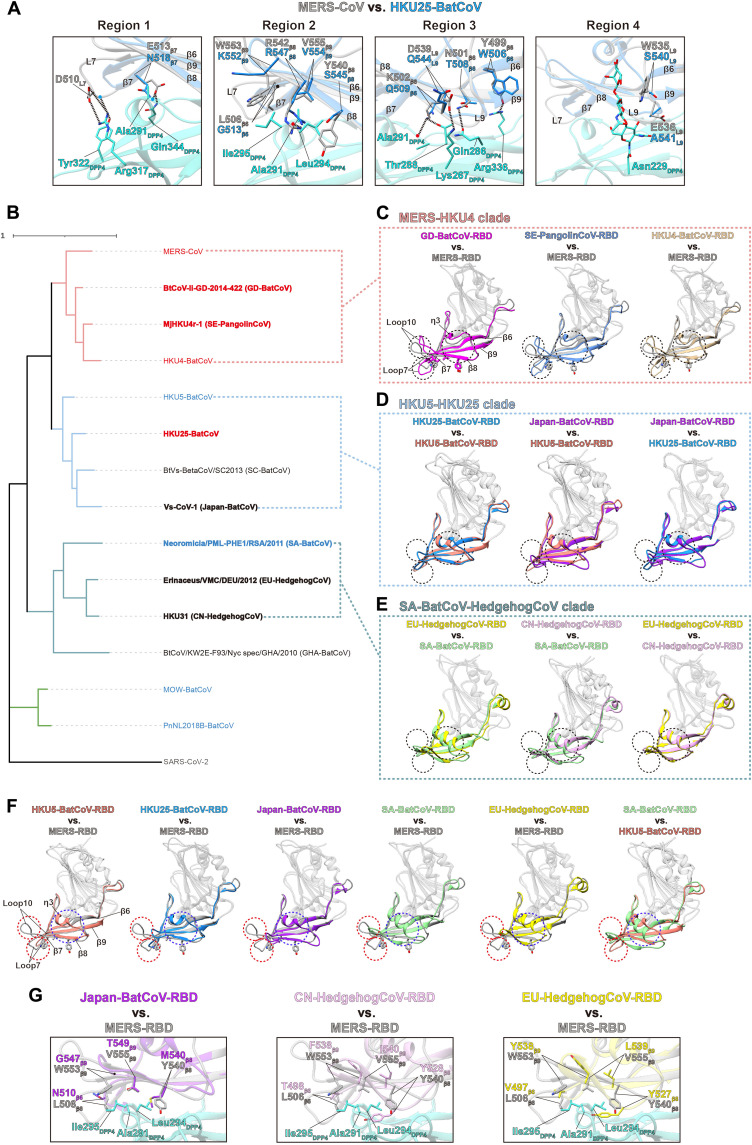
Comparison of different merbecovirus RBDs shows a convergent hDPP4 binding signature. (**A**) MERS-CoV-RBD residues (gray) interacting with hDPP4 residues (cyan) in the MERS-CoV-RBD:hDPP4 complex are compared to the corresponding residues of HKU25-BatCoV-RBD (blue). The comparison identifies substantial substitutions and positional deviations of the MERS-CoV-RBD residues involved in hDPP4 interactions. (**B**) Phylogenetic tree constructed based on representative merbecovirus RBD amino acid sequences, with SARS-CoV-2-RBD used as the outgroup; the MERS-HKU4, HKU5-HKU25, SA-BatCoV-HedgehogCoV, and MOW-BatCoV clades are colored red, blue, dark green, and green, respectively; bold virus names indicate S-trimer structures reported in this study. Names of hDPP4-binding and ACE2-binding viruses are shown in red and blue, respectively. (**C** to **E**) Pairwise comparisons of RBMs within each clade are shown. The core domains of RBDs are colored gray, while RBMs of different viruses are colored differently. Dashed circles mark the regions of Loop7, η3-β8, and Loop10. Amino acids in the hydrophobic cluster are shown as sticks in the RBD alignment of the MERS-HKU4 clade. (**F**) Pairwise comparisons of MERS-CoV-RBM with those of other non-hDPP4-binding viruses; structural variations in the RBM Loop7, η3-β8, and Loop10 regions are highlighted with dashed circles. Amino acids in the hydrophobic cluster are shown as sticks in the MERS-CoV-RBD. (**G**) MERS-CoV-RBD hydrophobic residues (gray) interacting with hDPP4 hydrophobic residues (cyan) in the MERS-CoV-RBD:hDPP4 complex are compared to the corresponding residues (colored) of Japan-BatCoV-RBD, CN-HedgehogCoV-RBD, and EU-HedgehogCoV-RBD. The comparison identifies substantial substitutions and positional deviations in residues critical for hDPP4 interaction.

We have shown that RBD-Fc of several merbecoviruses shows varied affinities toward hDPP4 (Fig. 1A), with strong binding observed for MERS-CoV and SE-PangolinCoV, and much weaker binding for HKU4-BatCoV and GD-BatCoV. High-affinity binding to hDPP4 is an important factor for MERS-CoV human infection (*30*). Although there has been a report suggesting that HKU25-BatCoV can use hDPP4 as the receptor (*29*), we were unable to detect hDPP4 binding by either its RBD-Fc or S-trimer. To explore potential interactions between HKU25-BatCoV-RBD and hDPP4, we docked the HKU25-BatCoV-RBD onto the MERS-CoV-RBD:hDPP4 complex (Fig. 6A). A comparison of MERS-CoV-RBD:hDPP4, GD-BatCoV-RBD:hDPP4, SE-PangolinCoV-RBD:hDPP4, and HKU4-BatCoV-RBD:hDPP4 complexes identifies that hydrophobic interactions mediated by the four functionally conserved hydrophobic RBD residues (L506_MERS_ from β6, Y540_MERS_ from β8, W553_MERS_ and V555_MERS_ from β9) are a shared feature at the interfaces between these RBDs and hDPP4 (Fig. 6C and fig. S5, A to D, region 2). In contrast, only V554_HKU25_ (corresponding to V555_MERS_) is maintained as hydrophobic in HKU25-BatCoV-RBD (Fig. 6A). Therefore, the lack of these hydrophobic interactions suggests that HKU25-BatCoV is unlikely to bind hDPP4 in the same mode as MERS-CoV, GD-BatCoV, SE-PangolinCoV, and HKU4-BatCoV.

Phylogenetic analysis indicates that the merbecovirus RBDs can be divided into at least four clades: MERS-HKU4 clade, HKU5-HKU25 clade, SA-BatCoV-HedgehogCoV clade, and MOW-BatCoV clade (Fig. 6B). Comparisons within respective clades reveal similarities in RBM structures and sequence lengths, whereas substantial structural and sequence differences exist between the RBMs of different clades (Fig. 6, C to E, and fig. S22). When comparing the MERS-CoV RBM with those of non-hDPP4–binding clades, the primary differences are observed in the Loop7, η3-β8, and Loop10 regions (Fig. 6F). Notably, Loop7 and Loop10 regions are longer in hDPP4-binding S-proteins but shorter in non-hDPP4–binding S-proteins. A recent study shows that ACE2 binding MOW-BatCoV RBM features extended Loop7 and Loop10 regions, which participate in ACE2 binding (*33*). In addition, η3-β8 shows a notable structural shift in non-hDPP4–binding S-proteins (Fig. 6F). RBD sequence alignment of the MERS-HKU4 clade identifies that the four functionally conserved hydrophobic residues (L506_MERS_, Y540_MERS_, W553_MERS_, and V555_MERS_) for hDPP4 binding are highly conserved in this clade (only 8 of the 60 sequences show nonhydrophobic amino acid substitutions at these positions) (fig. S23A). Among our determined MERS-CoV S-protein structures, receptors for Japan-BatCoV, CN-HedgehogCoV, and EU-HedgehogCoV remain unidentified. The comparison of their RBD structures with MERS-CoV-RBD shows that the four conserved hydrophobic residues are either substituted with nonhydrophobic amino acids or displaced in position (Fig. 6G).

Recent reports suggest that SA-BatCoV (*31*), MOW-BatCoV (*33*), and HKU5-BatCoV (*34*, *36*) bind to ACE2 using different binding modes. RBD alignment showed that HKU5-BatCoV (*54*) and SA-BatCoV also differ in Loop7, η3-β8, and Loop10 regions (Fig. 6F), while the sequence of MOW-BatCoV showed insertions in the Loop7 and Loop10. These results highlight the complexity of receptor binding in merbecoviruses, suggesting that the structural variations in different RBMs may reflect different receptor usage.

## DISCUSSION

In this study, we analyzed the structures of representative merbecovirus S-proteins from different clades and determined the cryo-EM structures of GD-BatCoV-RBD:hDPP4 and SE-PangolinCoV-RBD:hDPP4 complexes. Our findings reveal a substantial shift in the hDPP4-binding interface among different merbecovirus-RBD:hDPP4 complexes, with the GD-BatCoV-RBD:hDPP4 complex exhibiting the largest shift (4 to 6 Å) at the hDPP4 Arg336_DPP4_ loop (Fig. 1, F to H, and fig. S8). This structural divergence suggests a twisting motion of hDPP4 when bound to different RBDs. The motion originates from a conserved hydrophobic interaction cluster of RBM, where the motion is subtle near the cluster but becomes more pronounced distally, with obvious offsets near the hDPP4 279_DPP4_ and 333_DPP4_ loops (Fig. 1, F to H, and fig. S8). The twisting motion does not appear to correlate with hDPP4 affinity. The twisting motion is most noticeable when comparing hDPP4 complexes of GD-BatCoV and HKU4-BatCoV RBDs, both bind hDPP4 weakly. While hDPP4 binds similarly to both MERS-CoV and HKU4-BatCoV RBDs, with MERS-CoV demonstrates much stronger hDPP4 binding. There is also a twisting motion between hDPP4s bound to SE-PangolinCoV and MERS-CoV RBDs, both with strong binding. The twisting motion also appears to influence the role of the glycan at Asn229_DPP4_ in modulating RBD binding. The above analysis indicates a pivotal role for the hydrophobic interaction cluster in mediating the binding between hDPP4 and RBD. The sequence alignment of RBDs from the merbecovirus HKU4 clade identifies that putative hDPP4-interacting hydrophobic residues are highly conserved in positions corresponding to those in HKU4-BatCoV, GD-BatCoV, and MERS-CoV RBDs. In a few cases, substitutions are found (fig. S23A). For example, I557_GD_, which mediates hydrophobic interaction with hDPP4, has been found to change to a polar threonine (T) in a few GD-BatCoV–related sequences (*55*). We found I557T_GD_ substitution completely abolishes hDPP4 binding by GD-BatCoV-RBD (fig. S23B). These results highlight the critical importance of the hydrophobic interaction cluster for hDPP4 binding.

We identify a unique interaction between GD-BatCoV-RBD and hDPP4 involving Arg336_DPP4_. Previous studies have shown that mutation at Arg336_DPP4_ only weakly affects hDPP4 binding in SE-PangolinCoV-RBD (*45*). Instead, we found D541A_GD_ mutation in GD-BatCoV-RBD, abolishing its interaction with Arg336_DPP4_, abrogates hDPP4 binding. Arg336_DPP4_ varies substantially among different bat species (*56*), suggesting a potential role for Arg336_DPP4_ in modulating DPP4 binding among bats.

Our binding data show strong hDPP4 binding for SE-PangolinCoV-RBD and weak binding for GD-BatCoV-RBD. Phylogenetics shows that SE-PangolinCoV-RBD is closely related to HKU4-BatCoV-RBD within the same subclade, whereas MERS-CoV-RBD is more closely related to GD-BatCoV-RBD (Fig. 6B). Introducing hDPP4-interacting MERS-CoV-RBD residues into GD-BatCoV-RBD had minimal effect on hDPP4 binding. Introducing MERS-CoV-RBM fragments into GD-BatCoV-RBD even reduced or abolished hDPP4 binding. Only by swapping the complete MERS-CoV RBM into the GD-BatCoV RBD was hDPP4 binding substantially enhanced. To understand the enhanced hDPP4 binding of SE-PangolinCoV, hDPP4-interacting residues from the weak-binding HKU4-BatCoV-RBD were introduced into individual RBM regions of the SE-PangolinCoV RBD; single-region substitutions only subtly decreased hDPP4 binding, whereas simultaneous substitutions across multiple RBM regions led to a marked reduction. This observation indicates that interactions from different RBM regions work synergistically to mediate high-affinity hDPP4 binding, in line with our findings on sarbecoviruses (*44*). We also attempted to introduce SE-PangolinCoV-RBM residues into HKU4-BatCoV-RBM, but unfortunately, we were unable to obtain stable HKU4-BatCoV-RBD mutant proteins. These results demonstrate poor compatibility of RBM residues and structural motifs across different merbecoviruses, suggesting limited RBD plasticity and interchangeability even between closely related merbecoviruses.

Structural comparisons between hDPP4-binding and non-hDPP4–binding S-RBDs highlight key deletions and substitutions in Loop7, Loop10, and the η3-β8 region, which disrupt the hDPP4-binding surface, especially the conserved hydrophobic cluster interacting with hDPP4 (Fig. 6F). We and others have recently shown that restoring deletions in some sarbecovirus S-RBMs could substantially recover their affinity for ACE2 (*44*, *57*, *58*). We attempted to reintroduce Loop7 and Loop10 from MERS-CoV into CN-HedgehogCoV or to substitute the entire MERS-CoV RBD or RBM into CN-HedgehogCoV S-protein. However, the mutated proteins failed to express, further highlighting the limited plasticity of merbecovirus S-proteins and interchangeability of their RBDs. Some studies have shown that sarbecovirus RBMs or RBDs have greater plasticity and interchangeability (*44*, *57*–*60*). The limited plasticity of the merbecovirus S-proteins suggests that their receptor binding is highly specific and relies on specific overall RBM structural features that cannot be easily altered. The restricted interchangeability of merbecovirus RBM residues and fragments implies that receptor switching may require multiple coordinated RBM changes to gain compatibility with a new receptor. It remains unclear whether the substantial RBM changes that merbecoviruses require for receptor switching might limit their capacity for rapid host adaptation.

Within the merbecovirus subgenus, the currently identified ACE2-binding strains belong to three distinct clades, each exhibiting a unique binding mode. Comparisons of RBM structures between clades reveal substantial differences in their RBMs with different ACE2-binding modes (Fig. 6, D to F, and fig. S24). To date, Japan-BatCoV (HKU5-HKU25 clade), CN-HedgehogCoV, and EU-HedgehogCoV (SA-BatCoV-HedgehogCoV clade) have not been shown to bind ACE2. Structural comparison also shows subtle RBM variations within clades (Fig. 6, D to E), suggesting that merbecoviruses may alter their receptor usage through amino acid variations in the RBM that result in minor structural changes during evolution. Our comparison also reveals that RBM structures within MERS-HKU4 clade of hDPP4-binding viruses are similar, featuring a conserved cluster of hydrophobic residues that interact with hDPP4 (Fig. 6C). DPP4-binding merbecoviruses from different hosts engage DPP4 receptors of different species through structurally similar binding modes, indicating the convergent evolution of DPP4 utilization within the MERS-HKU4 clade (fig. S24). In summary, there appears to be strong convergent evolution in DPP4-binding mode within the MERS-HKU4 clade, while divergent evolution in ACE2 binding mode is observed across the HKU5-HKU25, SA-BatCoV-HedgehogCoV, and MOW-BatCoV clades, with possible further divergence in receptor adaptation within each clade (fig. S24). Together, these results emphasize the complexity of merbecovirus receptor binding and highlight the necessity for further research into merbecovirus receptor usage to better understand their cross-species transmission and pandemic potential. Of note, a recent preprint reports that HKU25-CoV binds ACE2 as the receptor (*61*).

## MATERIALS AND METHODS

### Expression constructs

The S-protein genes of BtCoV/Ii/GD/2014–422/*I. io*/Guangdong/2014 (GD-BatCoV, accession number: AVV62537.1), HKU4/*T. pachypus*/Guangdong/2007 (HKU4-BatCoV, accession number: YP_001039953.1), HKU5/Japanese pipistrelle/Guangdong/2007 (HKU5-BatCoV, accession number: YP_001039962.1), HKU25/*H. pulveratus*/Guangdong/2014 (HKU25-BatCoV, accession number: ASL68953.1), BtVs-BetaCoV/SC2013/*V. superans*/Sichuan/2014 (SC-BatCoV, accession number: AHY61337.1), BtCoV/KW2E-F93/Nyc_spec/GHA/2010/*Nycteris sp.*/Ghana/2011 (GHA-BatCoV, accession number: AGC51116.1), Neoromicia/PML-PHE1/RSA/2011/*N. capensis*/South Africa/2011 (SA-BatCoV, accession number: AGY29650.2), Vs-CoV-1/*V. sinensis*/Japan/2019 (Japan-BatCoV, accession number: BBJ36008.1), HKU31/*E. amurensis*/China/2014 (CN-HedgehogCoV, accession number: QGA70692.1), Erinaceus/VMC/DEU/2012/*E. europaeus*/Germany/2012 (EU-HedgehogCoV, accession number: AGX27810.1) were retrieved from the GenBank database. The S-protein gene of MjHKU4r-CoV-1/*M. javanica*/Southeast Asia/2023 (SE-PangolinCoV, accession number: UVJ46720.1) has the same sequence as reported previously (*30*).

The genes encoding these different S-proteins with their native N-terminal signal peptide, a C-terminal T4 trimerization foldon, an HRV 3C cleavage site, an Octo-His tag, and a Twin-Strep-tag were synthesized by Sangon Biotech and cloned into the expression vector pcDNA3.1+. To enable secretion of the ectodomains of these S-proteins, the transmembrane domains and C-terminal ends were removed. The Japan-BatCoV, CN-HedgehogCoV, EU-HedgehogCoV, and SA-BatCoV S-proteins had no modification in their amino acid sequences. For improved protein stability, the following modifications were made. The multibasic site “RVRR” in the HKU5-BatCoV S-protein was modified to “AVRS.” The six proline mutations (A885P_GD_, S962P_GD_, A969P_GD_, N1012P_GD_, A1056P_GD_, and V1057P_GD_) were introduced into the GD-BatCoV S-protein (GD-BatCoV-S-6P) (*51*, *62*). For structural study, the HKU25-BatCoV S-protein contained the double proline mutations (A1052P_HKU25_ and V1053P_HKU25_) (*51*) and an engineered disulfide bond (S437C_HKU25_ and D1052C_HKU25_) (HKU25-BatCoV-S-2P-x1) (*20*, *48*, *63*). For binding study, the HKU25-BatCoV S-protein only contained the double proline mutations (A1052P_HKU25_ and V1053P_HKU25_) (HKU25-BatCoV-S-2P).

The coding sequence for human DPP4 (GenBank accession number NP_001926, residues 39 to 766) with a CD5 signal peptide was synthesized and cloned into the vector pcDNA3.1+. The C terminus of hDPP4 was fused to the Fc domain of human immunoglobulin G (IgG), resulting in the hDPP4-Fc construct. In addition, residues 39 to 766 of hDPP4, which include its dimerization domain, were fused to a C-terminal Hexa-His tag to construct the dimeric hDPP4-his protein. For fusion assays, a full-length hDPP4 construct with an N-terminal Flag tag was prepared. All gene sequences were codon-optimized for human cells.

The monomeric RBDs of MERS-CoV (amino acids 377 to 588), GD-BatCoV (amino acids 371 to 610), SE-PangolinCoV (amino acids 375 to 614), HKU4-BatCoV (amino acids 372 to 611), and HKU25-BatCoV (amino acids 375 to 605) were fused with C-terminal Hexa-His tags for expression. In addition, the dimeric RBDs of MERS-CoV (amino acids 377 to 588), GD-BatCoV (amino acids 371 to 610), SE-PangolinCoV (amino acids 375 to 614), HKU4-BatCoV (amino acids 372 to 611), HKU25-BatCoV (amino acids 375 to 605), and RBM fragment-swap mutants of GD-BatCoV and MERS-CoV, each fused to an Fc domain of human IgG, were cloned into pcDNA3.1+.

### Protein expression and purification

His-tagged proteins were expressed by transiently transfecting corresponding plasmids into Expi293 cells using PEI (linear polyethylenimine) transfection reagent. One liter of cells was transfected with 1 mg of plasmid DNA. Five days post-transfection at 33°C, supernatants were harvested by centrifugation and supplemented with 25 mM phosphate (pH 8.0), 5 mM imidazole, 300 mM NaCl, and 0.5 mM phenylmethylsulfonyl fluoride (PMSF). Supernatants were recirculated onto a HiTrap TALON crude column (Cytiva) for three cycles. Subsequently, the column was washed by 100 ml of buffer A [25 mM phosphate, (pH 8.0), 5 mM imidazole, and 300 mM NaCl], and proteins were eluted by a 100 ml of linear gradient to 100% buffer B [25 mM phosphate (pH 8.0), 500 mM imidazole, and 300 mM NaCl]. The EU-HedgehogCoV S-protein was purified using a modified buffer A [25 mM phosphate (pH 6.0), 5 mM imidazole, and 300 mM NaCl]. Fractions containing the proteins were pooled, concentrated with 100 kDa (for S-proteins) or 10 kDa (for RBD proteins) MWCO Amicon Ultra filtration device (Merck Millipore, Burlington, MA, USA), and buffer-exchanged into phosphate-buffered saline (PBS) [10 mM Na_2_HPO_4_ and 1.8 mM KH_2_PO_4_ (pH 7.4), 137 mM NaCl, and 2.7 mM KCl]. Purified proteins were quality checked by SDS–polyacrylamide gel electrophoresis and frozen at −80°C until further use.

For the purification of hDPP4-Fc protein, transfection was performed as described above. Supernatant was collected and recirculated onto a Protein A column (Cytiva). The column was washed with PBS (Gibco, Thermo Fisher Scientific, Waltham, MA, USA) before protein was eluted with 0.1 M citric acid (pH 3.0). Fractions containing hDPP4-Fc were neutralized with 1 M tris-HCl (pH 8.0), concentrated, and buffer exchanged into PBS. The RBD-Fc proteins were purified using the same method. Purified proteins were frozen in liquid nitrogen and stored at −80°C.

### BLI binding assay

Binding kinetics and affinities of hDPP4 toward S-proteins, dimeric RBDs, or monomeric RBDs were assessed by BLI on an Octet RED96 instrument (Sartorius). All experiment procedures were conducted at 25°C, and reagents were prepared in PBS-TB buffer [PBS containing 0.02% (v/v) Tween 20 and 0.1% (w/v) bovine serum albumin (BSA)]. Biosensors were pre-equilibrated in the PBS-TB buffer for 10 min before use. hDPP4-Fc protein (10 μg/ml) was immobilized onto Protein A biosensors (Sartorius). The hDPP4-loaded biosensors were exposed to the wells containing S-trimers or monomeric RBDs for 300 s to record association (S-trimers were diluted in threefold serial dilutions from 800 to 1.1 nM, and monomeric RBDs were diluted in threefold serial dilutions from 1500 nM to 2.06 nM). Subsequently, the biosensors were dipped into PBS-TB for 600 s to observe dissociation. Uncoated biosensors were also submerged into the S-trimer/RBD solutions and PBS-TB buffer to record background. Dimeric RBDs with Fc-tag were loaded onto Protein A biosensors and dipped into wells containing hDPP4-his protein with concentrations ranging from 800 to 1.1 nM, following the above procedure. Data were background-subtracted and analyzed using Data Analysis HT version 12.0 software (Sartorius), fitting the data to 1:1 or 2:1 binding models to determine kinetic parameters. For hDPP4-Fc binding by monomeric RBD proteins, no binding is defined as response values below 0.05 nm. For dimeric RBD-Fc proteins binding by dimeric hDPP4-his, weak binding is defined as response values between 0.05 and 0.25 nm, while values below 0.05 nm defined as no binding. Results were plotted using GraphPad Prism 8.0. BLI assays were repeated at least twice.

### Cryo-EM sample preparation and data collection

For cryo-EM sample preparation, the S-proteins were concentrated to 0.9 to 2.6 mg/ml for CN-HedgehogCoV, EU-HedgehogCoV, SA-BatCoV, Japan-BatCoV, and HKU25-BatCoV. For the structures of GD-BatCoV S-trimer and GD-BatCoV-RBD:hDPP4 complex, GD-BatCoV-6P-S at a concentration of 1.2 mg/ml was mixed with hDPP4-Fc at 1:1 molar ratio for 2 min. For the SE-PangolinCoV-RBD:hDPP4 complex, SE-PangolinCoV-RBD-Fc at a concentration of 0.8 mg/ml was mixed with hDPP4-his at 1:1 molar ratio for 2 hours. The above protein solutions (3 μl) were applied to glow-discharged (15 mA, 30 s) holey carbon grids (Quantifoil, Cu R1.2/R1.3). The grids were blotted for 2.5 s with a force of 4 and then flash plunged in liquid ethane using a Vitrobot Mark IV (Thermo Fisher Scientific) at 4°C and 100% humidity. Data collection was performed on a 200-keV Talos Arctica electron microscope (Thermo Fisher Scientific) equipped with a K3 direct detection camera (Gatan) using Serial EM software. In the super resolution mode, images were recorded at a magnification of ×45,000 with a defocus range of −0.8 to −2.5 μm. For the SE-PangolinCoV-RBD:hDPP4 complex, images were recorded at a magnification of ×45,000 with a defocus range of −0.8 to −1.6 μm. Movies were collected with a dose rate of 25 e^−^/pixel/s and were fractionated into 27 frames and each frame exposed for 0.7 s, resulting in a total dose of 60 e^−^/Å^2^ with a calibrated pixel size of 0.88 Å.

### Cryo-EM data processing

Motion correction for cryo-EM images was performed using MotionCor2 in RELION-3.1 or RELION-4.0 (*64*–*66*). Contrast transfer function (CTF) estimation and template-free particle picking were carried out in Warp (*67*) for CN-HedgehogCoV-S, EU-HedgehogCoV-S, and SA-BatCoV-S datasets. For each dataset, a locked conformation SARS-CoV-2 S-trimer structure (EMD-11329) (*20*) was filtered to 60 Å resolution to serve as the reference map for the first round of three-dimensional (3D) classification. 3D classes exhibiting clear secondary structures were selected and subjected to one round of 2D classification to exclude poor-quality particles, followed by a second round of 3D classification. Auto-refinement, CTF refinement, and Bayesian polishing were performed for the classified datasets. The final map was sharpened using RELION after the final round of 3D auto-refinement.

After motion correction by MotionCor2, the datasets for Japan-BatCoV-S and GD-BatCoV-S/GD-BatCoV-RBD:hDPP4 underwent CTF estimation using CTFFIND-4.1 (*68*). Particles were automatically picked using RELION. The first round of 2D classification and 3D classification were performed to remove bad particles. Subsequently, high-quality particles were selected through a second round of 2D and 3D classification before being subjected to 3D auto-refinement. CTF refinement and Bayesian polishing were applied to the classified datasets with the final maps sharpened.

For the HKU25-BatCoV-S-2P-x1 and SE-PangolinCoV-RBD:hDPP4 datasets, the motion correction for cryo-EM images was performed using MotionCor2 in RELION. CTF estimation, blob picker, particle extraction, and the first round of reference-free 2D classification were carried out in CryoSPARC (*69*). Following a template picker and the second round of 2D classification, well-featured particles were selected for ab-initio reconstruction or homogeneous refinement. Refined particles were selected for a round of topaz training before particles were repicked and 2D classified for the third round. For HKU25-BatCoV-S-2P-x1 dataset, ab-initio reconstruction and non-uniform refinement were performed to generate the final map. For SE-PangolinCoV-RBD:hDPP4 dataset, homogeneous refinement, 3D classification, and non-uniform refinement were performed to obtain a final map.

Local resolution estimation was performed using RELION or CryoSPARC. Map resolutions were estimated at the 0.143 criterion of the phase-randomization–corrected Fourier shell correlation curve.

### Model building and refinement

The MERS-CoV S-protein structure [Protein Data Bank (PDB): 6Q04] (*21*) was used as the starting model for the studied merbecovirus S-trimer structures. The MERS-CoV-RBD:hDPP4 (*24*) (PDB: 4KR0) complex structure was as the starting model for GD-BatCoV-RBD:hDPP4 and SE-PangolinCoV-RBD:hDPP4 complex structures. The starting models for the S-proteins and complex structures were fitted into the final maps using UCSF Chimera (*70*), with manual adjustments in Coot (*71*). All models were refined in Namdinator (*72*) and PHENIX (*73*). All figures were generated in UCSF Chimera. Model refinement statistics are provided in table S9.

### Cell-cell fusion assay

Cell-cell fusion was detected using a Split-GFP system (*74*). The S-protein and pQCXIP-GFP1-10 expression plasmids were cotransfected into 293T cells as effector cells, whereas the hDPP4 expression plasmid or hDPP4 + TMPRSS2 expression plasmids, along with pQCXIP-BSR-GFP11 plasmid were cotransfected into 293T cells as receptor cells. Following 24 hours post-transfection, both types of cells were harvested, washed twice with PBS, and resuspended in Dulbecco’s Modified Eagle Medium. To test the effect of trypsin, the effector cells were incubated with L-1-tosylamido-2-phenylethyl chloromethyl ketone (TPCK)-treated trypsin (5 μg/ml) at 37°C for 10 min before cells were harvested and washed. The effector cells and receptor cells were mixed at a 2:1 ratio and plated in a 96-well cell plate, and fluorescence imaging was performed at 2, 8, 16, and 24 hours after mixing.

### Flow cytometry

Flow cytometry was conducted using a BD Accuri C6 Plus cytometer. Data were analyzed with FlowJo software. 293T cells transfected with S-proteins for 24 hours were washed in PBS with 1% BSA. The cells expressing S-proteins were incubated with hDPP4-Fc protein on ice for 1 hour and washed twice with PBS, before being incubated with Allophycocyanin (APC)-conjugated goat anti-human IgG Fc (1:750; BioLegend) for 45 min on ice.

### RBD sequence analyses

A total of 168 merbecovirus RBD amino acid sequences were retrieved from the GenBank by BLAST against the MERS-CoV-RBD (residues 381 to 587) amino acid sequence. Sequence collection was finalized in March 2025. To perform the clustering analysis (fig. S24B), 168 merbecovirus RBD sequences were aligned using Multiple Alignment using Fast Fourier Transform (MAFFT) (*75*) in Jalview (*76*). A phylogenetic tree (fig. S24A) was generated using IQ-TREE2 (*77*, *78*) (1000 Bootstraps) (http://iqtree.cibiv.univie.ac.at/). The resulting tree was refined and visualized using iTOL v6 (*79*) (https://itol.embl.de/). Pairwise sequence similarity matrices were generated using BioEdit (v7.1.3.0) (*80*), with similarity scores used as proxies for genetic distances. Dimensionality reduction and *K*-means clustering were subsequently performed in R to determine the optimal *K* value and to visualize the sequence grouping patterns.

A smaller phylogenetic tree (Fig. 6B) was generated using RBD sequences representing a subset of the 168 merbecovirus RBD sequences. All sequences were trimmed to match MERS-CoV-RBD (residues 381 to 587), with the SARS-CoV-2 RBD sequence used as an outgroup. Sequence alignment was performed using ClustalW in MEGA11 (*81*). The tree was generated using the maximal likelihood method with Poisson model in MEGA11 (1000 Bootstraps).

Multiple sequence alignments (figs. S1, S9, S19, S22, and S23) were generated using Clustal Omega (*82*) (www.ebi.ac.uk/jdispatcher/msa/clustalo) and further processed with ESPript (*83*) (https://espript.ibcp.fr) for visualization.
