# Supplementary material for: Structures and receptor binding activities of merbecovirus spike proteins reveal key signatures for human DPP4 adaptation
Source: Sci Adv. 2025 Jul 11;11(28):eadv7296. doi: 10.1126/sciadv.adv7296 (PMC12248280; doi:10.1126/sciadv.adv7296)
Supplement: Supplementary file 1 — Figs. S1 to S24 Tables S1 to S9 [file sciadv.adv7296_sm.pdf]

Science Advances
AAAS

Supplementary Materials for

Structures and receptor binding activities of merbecovirus spike proteins reveal key signatures for human DPP4 adaptation

Hang Yuan *et al.*

Corresponding author: Peng Zhou, zhou_peng@gzlab.ac.cn; Zheng-Li Shi, shi_zhengli@gzlab.ac.cn; Xinwen Chen, chen_xinwen@gzlab.ac.cn; Xiaoli Xiong, xiong_xiaoli@gibh.ac.cn

Sci. Adv. **11**, eadv7296 (2025)
DOI: 10.1126/sciadv.adv7296

This PDF file includes:

Figs. S1 to S24
Tables S1 to S9

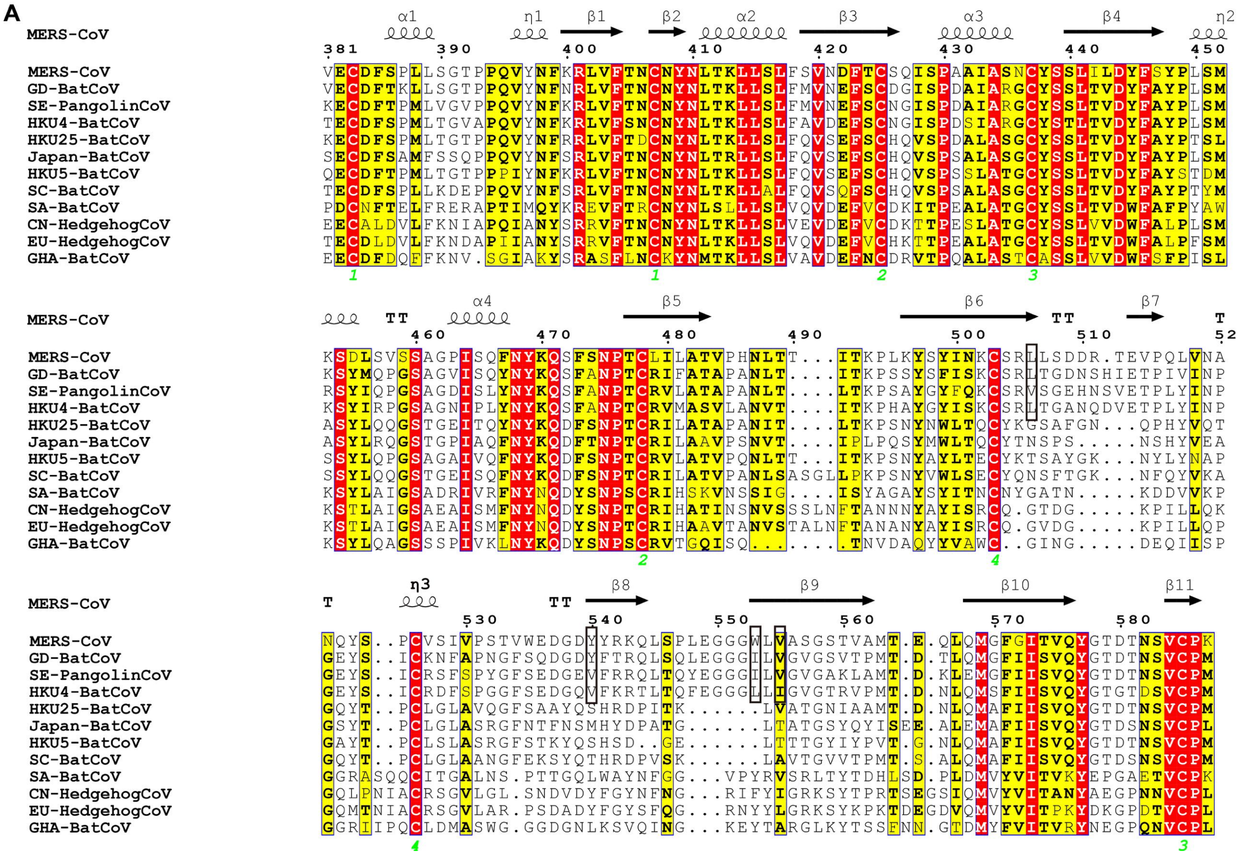

Fig. S1. Alignment of merbecovirus RBD sequences used in this study. The secondary structure elements are defined by ESPript (https://espript.ibcp.fr) using the structure of MERS-CoV-RBD (PDB:4KR0). Red and yellow shades indicate absolutely conserved and partially conserved residues. Pairs of green digits below the sequences indicate disulfide bond pairs.

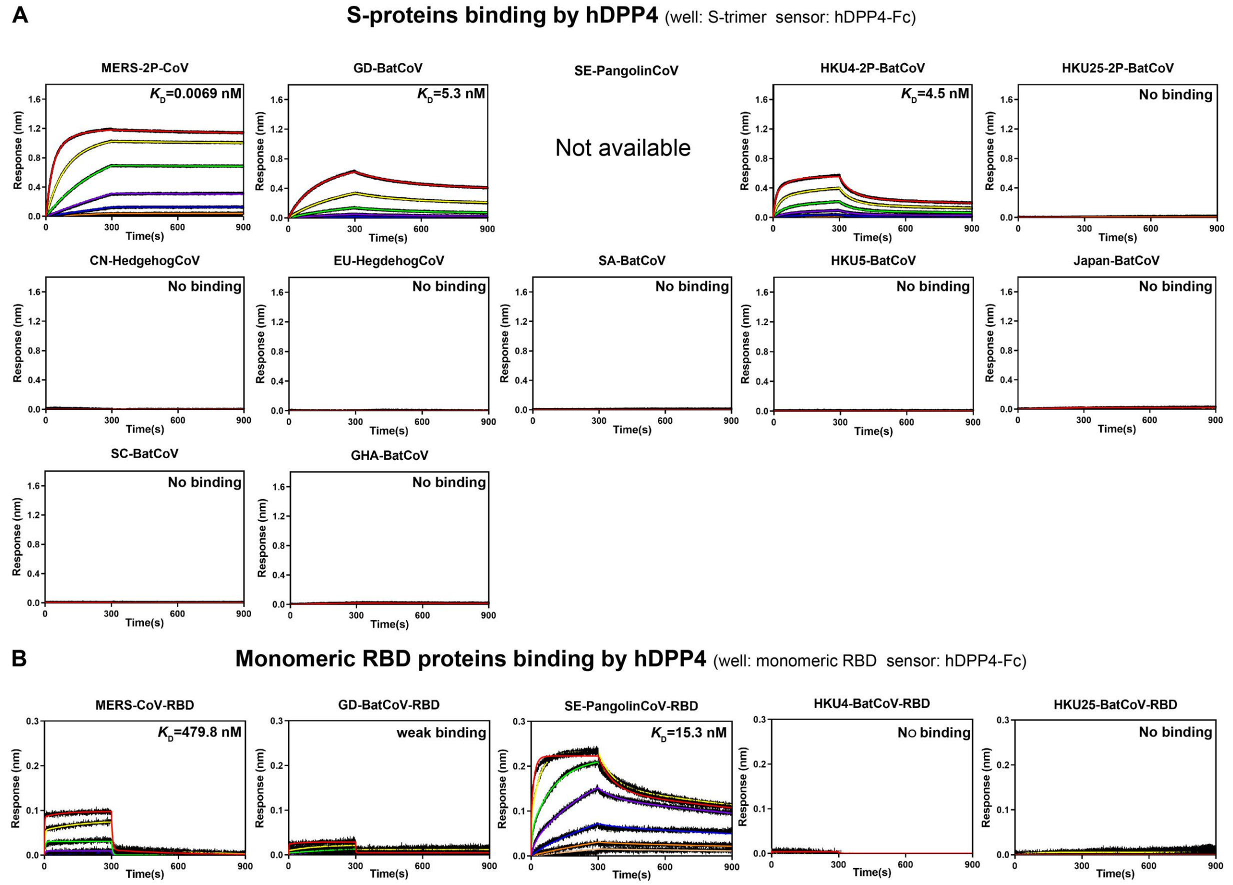

Fig. S2. Binding of hDPP4 by merbecovirus S-trimers or monomeric RBDs. (A) Binding of hDPP4 by merbecovirus S-proteins. Locations of proteins in the BLI assays are indicated by the (well) or (sensor) labels. hDPP4-Fc protein was immobilized on Protein A biosensors and exposed to the wells containing S-proteins in a 3-fold dilution series (800 nM to 1.1 nM). **(B)** Binding of hDPP4 by the monomeric RBDs of MERS-CoV, GD-BatCoV, SE-PangolinCoV, HKU4-BatCoV, and HKU25-BatCoV. hDPP4-Fc protein was immobilized on Protein A biosensors and exposed to the wells containing monomeric RBDs in a 3-fold dilution series (1500 nM to 2.1 nM). K_D values are shown alongside the binding curves. Binding kinetic parameters are summarized in table S2.

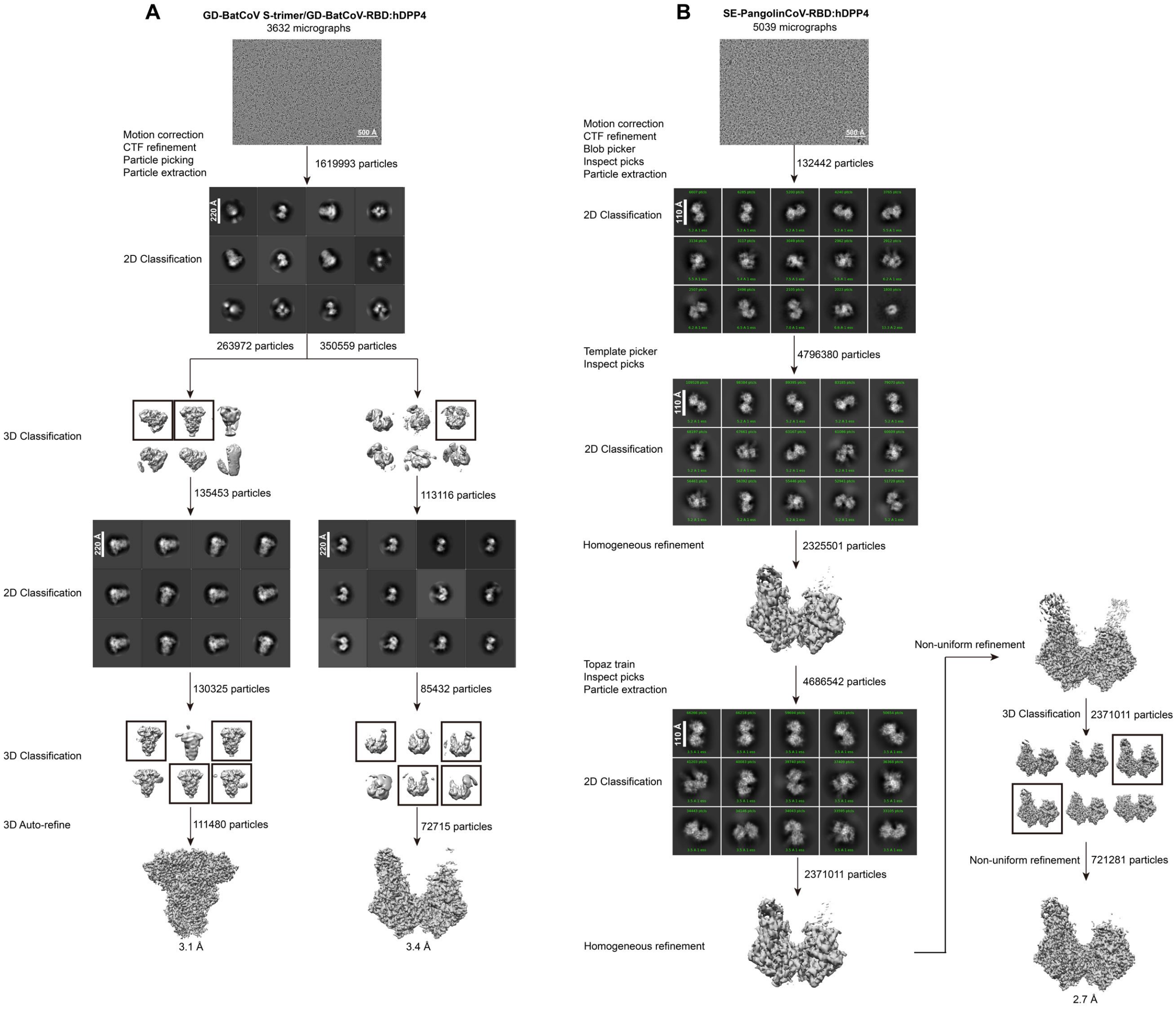

Fig. S3. Cryo-EM data processing flow-chart for the GD-BatCoV S-trimer structure and the GD-BatCoV-RBD:hDPP4, SE-PangolinCoV-RBD:hDPP4 complex structures. (A) Cryo-EM processing pipeline for the GD-BatCoV S-trimer and GD-BatCoV-RBD:hDPP4 structures. After particle picking, 2D and 3D classification procedures were performed to remove bad particles before a round of 3D auto-refine was performed to obtain the final maps. **(B)** Cryo-EM processing pipeline for the SE-PangolinCoV-RBD:hDPP4 structure. After two rounds of 2D classification and one round of homogeneous refinement, good particles were subjected to topaz train and particle re-picking. 2D and 3D classification procedures were performed again to remove bad particles. The final map was obtained by non-uniform refinement.

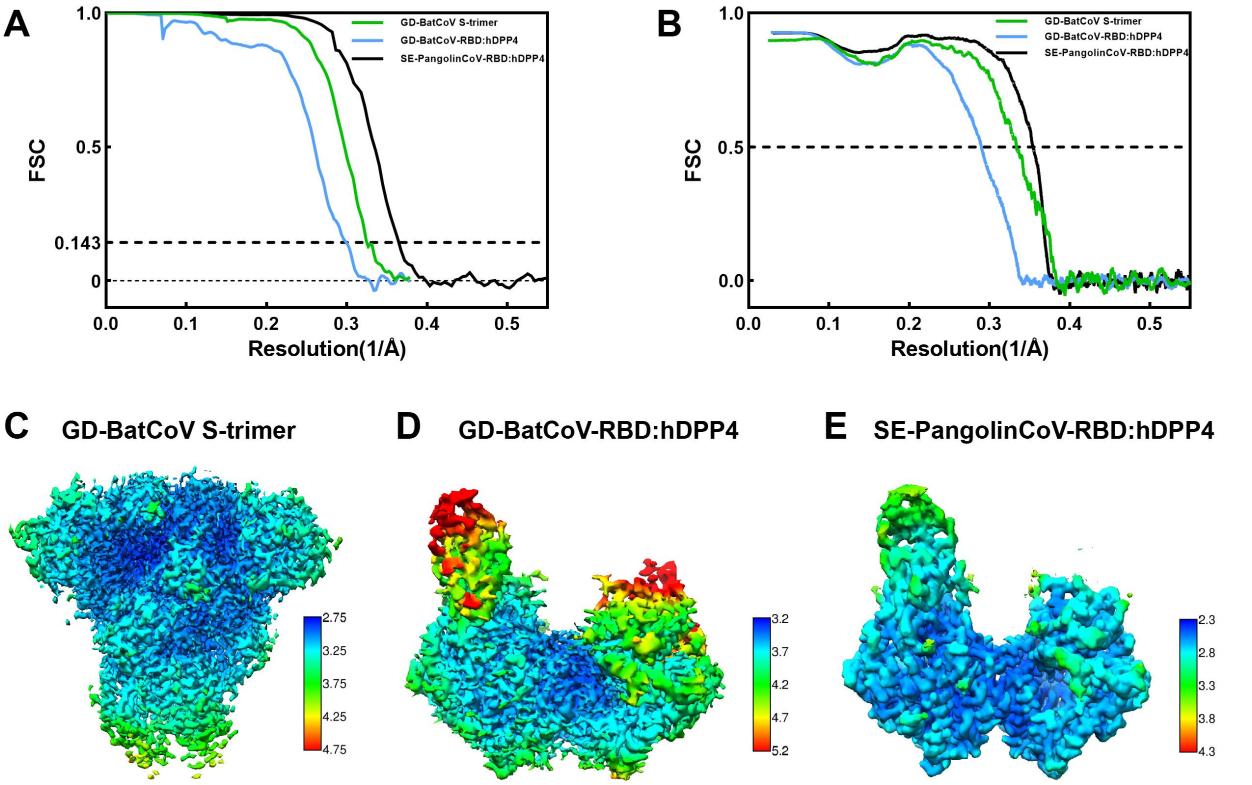

Fig. S4. Resolution assessments of the GD-BatCoV S-trimer structure and the GD-BatCoV-RBD:hDPP4 and SE-PangolinCoV-RBD:hDPP4 complex structures. (A) Global resolution assessment by Fourier shell correlation at the 0.143 criterion. **(B)** Correlations of model-to-map by Fourier shell correlation at the 0.5 criterion. **(C-E)** Local resolution maps for the GD-BatCoV S-trimer **(C)**, GD-BatCoV-RBD:hDPP4 **(D)** and SE-PangolinCoV-RBD:hDPP4 **(E)** structures.

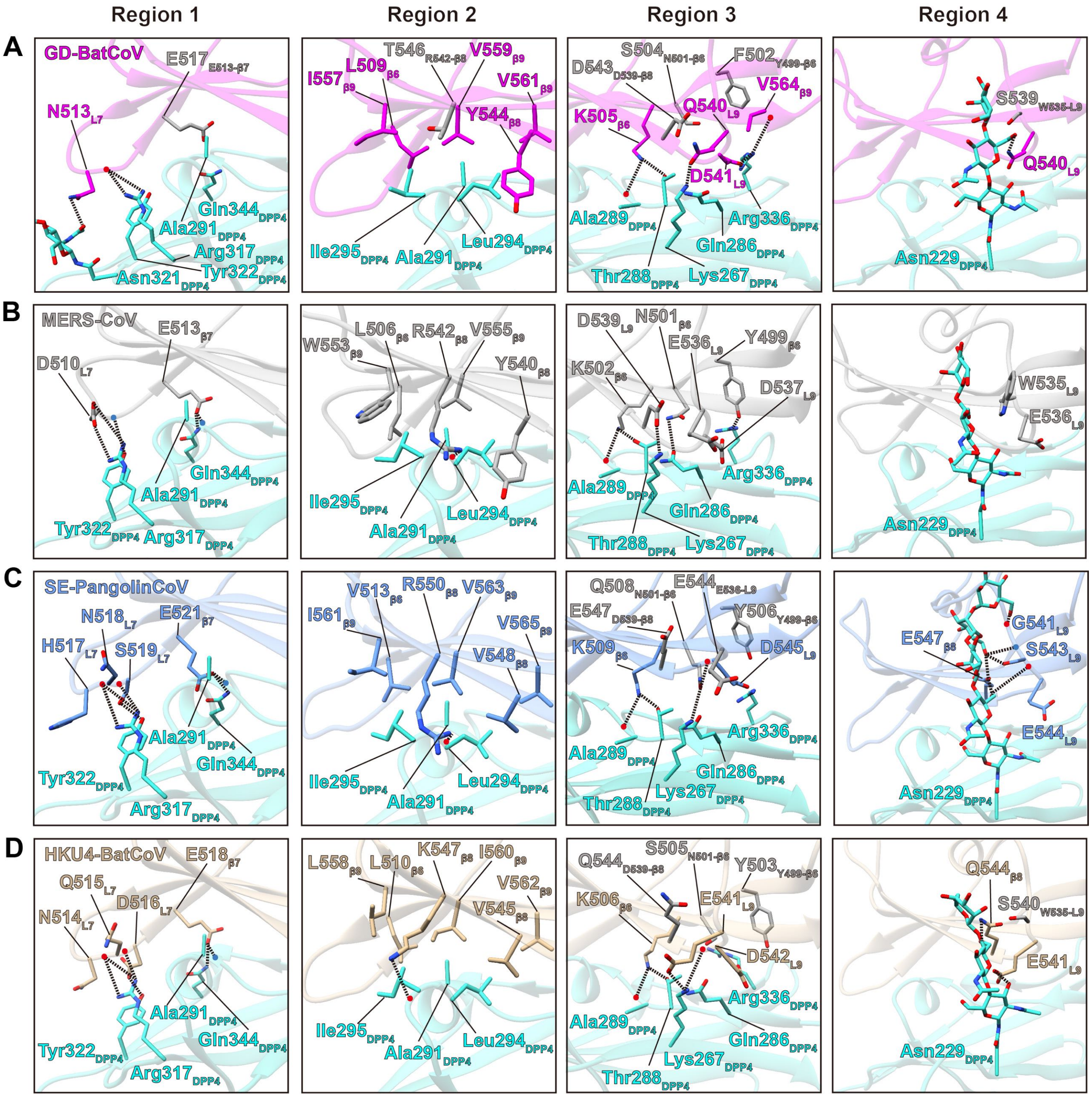

Fig. S5. Comparison of the binding interfaces in the GD-BatCoV-RBD:hDPP4, MERS-CoV-RBD:hDPP4, SE-PangolinCoV-RBD:hDPP4, and HKU4-BatCoV-RBD:hDPP4 structures. (A-D) The binding interfaces of GD-BatCoV, MERS-CoV, SE-PangolinCoV, and HKU4-BatCoV RBDs to hDPP4 were divided into four regions and analyzed in detail. The RBDs of GD-BatCoV, MERS-CoV, SE-PangolinCoV, and HKU4-BatCoV are colored magenta, grey, blue and beige, respectively. The hDPP4 is colored cyan. Amino acids forming the binding interfaces are shown as sticks. Amino acid residues in GD-BatCoV, SE-PangolinCoV, and HKU4-BatCoV RBDs that are not directly involved in receptor binding, but correspond to residues in the MERS-CoV RBD that are involved in receptor binding, are shown as grey sticks for comparison. Dashes represent hydrogen bonds, with blue and red dots indicating backbone amide and carbonyl atoms, respectively.

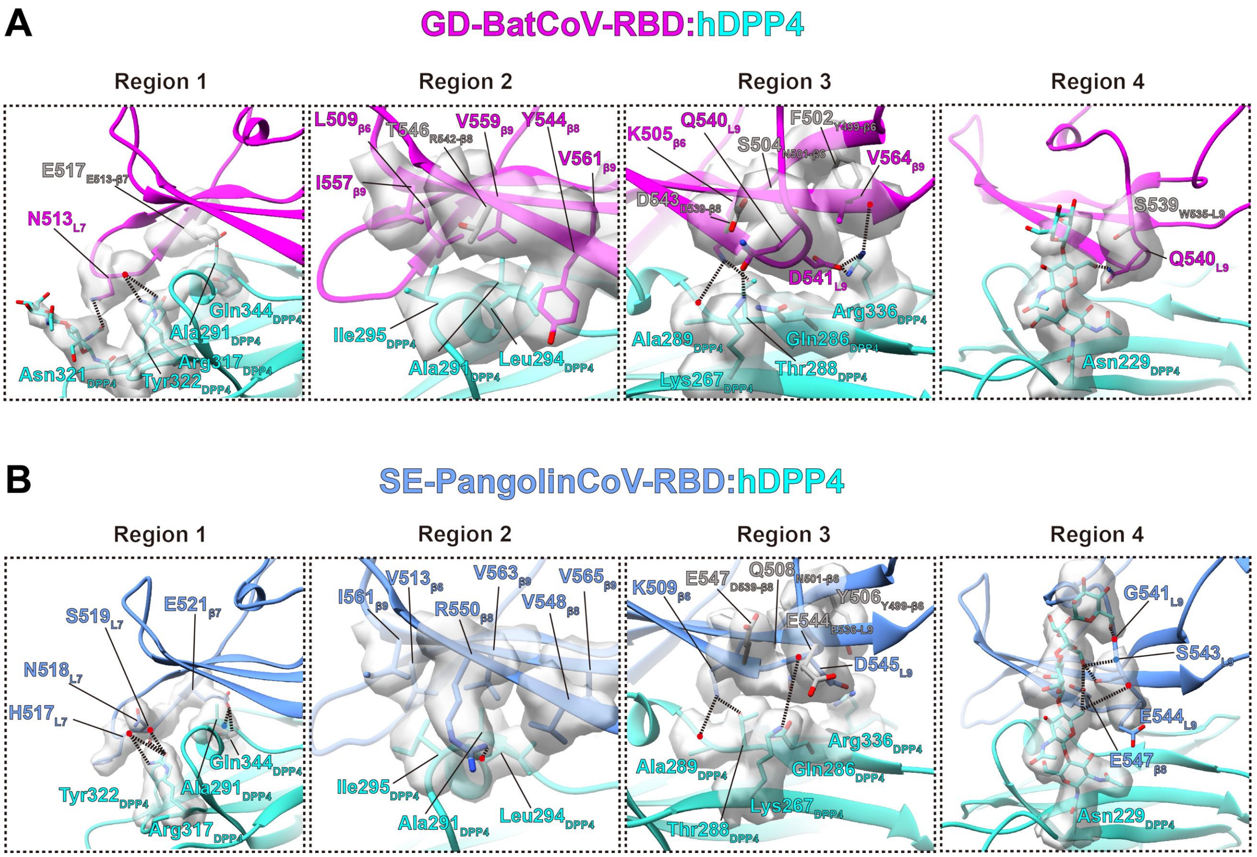

Fig. S6. Cryo- EM densities for the interacting residues at the binding interfaces of GD-BatCoV-RBD:hDPP4 and SE-PangolinCoV-RBD:hDPP4 complexes. (A-B) The GD-BatCoV-RBD, SE-PangolinCoV-RBD, and hDPP4 are highlighted in magenta, blue and cyan, respectively. Residues at the binding interfaces are related to Fig. 1D-E and fig. S5.

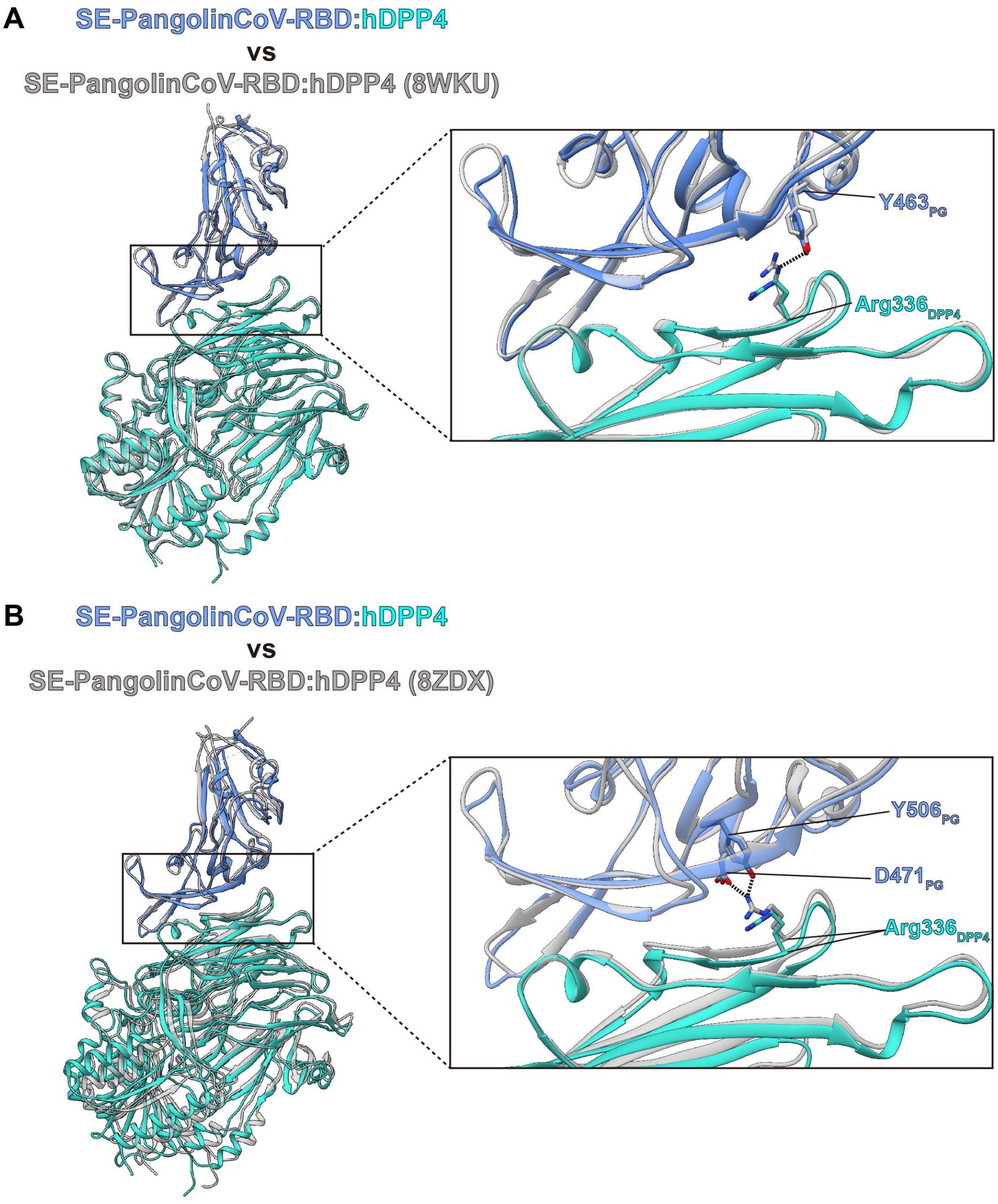

Fig. S7. Variations in the hDPP4 interaction interface between cryo-EM and X-ray structures of the SE-PangolinCoV-RBD:hDPP4 complex. (A) Interaction of $Arg336_{DPP4}$ in the SE-PangolinCoV-RBD:hDPP4 X-ray structure 8WKU. Sidechains of $Y463_{PG}$ and $Arg336_{DPP4}$ are shown in the right panel. $Y463_{PG}$ and $Arg336_{DPP4}$ are close enough in the X-ray structure (PDB:8WKU) to form hydrogen bond, whereas the distance between $Y463_{PG}$ and $Arg336_{DPP4}$ in the cryo-EM structure is substantially longer, preventing hydrogen bond formation. **(B)** Interaction of $Arg336_{DPP4}$ in the SE-PangolinCoV-RBD:hDPP4 X-ray structure 8ZDX. Residues $D471_{PG}$, $Y506_{PG}$ and $Arg336_{DPP4}$ are shown in the right panel. In the X-ray structure (PDB: 8ZDX), the distances of $D471_{PG}$ and $Y506_{PG}$ to $Arg336_{DPP4}$ are close enough to form a salt-bridge and a

hydrogen bond. However, in the cryo-EM structure, the distances are longer, preventing these interactions.

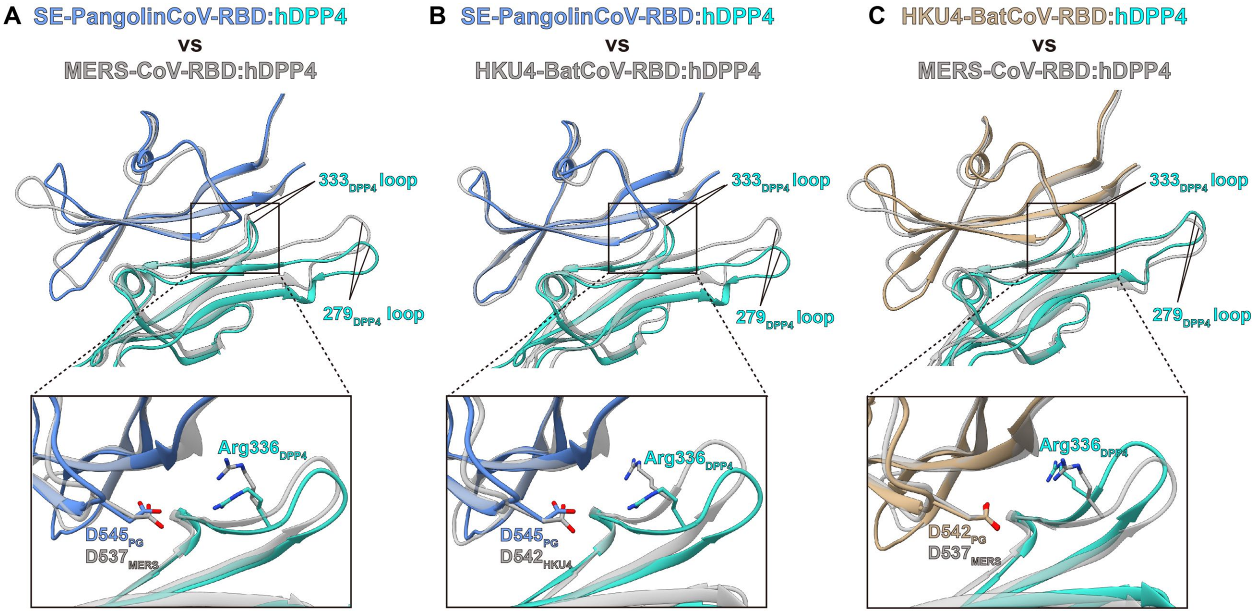

D

RMSD	DPP4 (MERS-CoV)	DPP4 (GD-BatCoV)	DPP4 (SE-PangolinCoV)	DPP4 (HKU4-BatCoV)
DPP4 (MERS-CoV)		6.498	5.102	3.718
DPP4 (GD-BatCoV)	6.498		5.776	9.126
DPP4 (SE-PangolinCoV)	5.102	5.776		5.574
DPP4 (HKU4-BatCoV)	3.718	9.126	5.574	

Fig. S8. Structural comparison reveals the offset of hDPP4 interaction interfaces among the SE-PangolinCoV-RBD:hDPP4, MERS-CoV-RBD:hDPP4, and HKU4-BatCoV-RBD:hDPP4 complexes. (A-C) Pairwise comparisons of the SE-PangolinCoV-RBD:hDPP4, MERS-CoV-RBD:hDPP4, and HKU4-BatCoV-RBD:hDPP4 complexes. The RBM region was used as the alignment reference. Top panels illustrate differences in the overall structures among SE-PangolinCoV-RBD:hDPP4, MERS-CoV-RBD:hDPP4, and HKU4-BatCoV-RBD:hDPP4 complexes with the 279_{DPP4} and 333_{DPP4} loops highlighted with labels. Bottom panels highlight the variation in positions for equivalent RBD residues $D545_{PG}$, $D537_{MERS}$, $D542_{HKU4}$ and hDPP4 residue $Arg336_{DPP4}$, among these complexes. **(D)** RMSDs of hDPP4 with the RBM as the alignment reference.

A

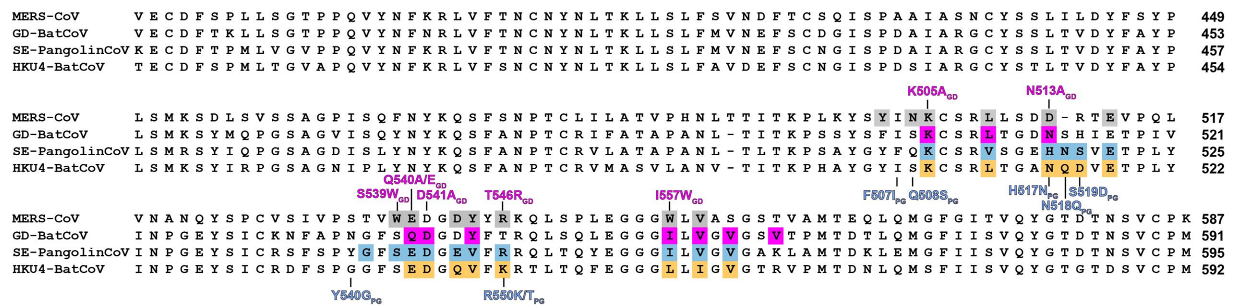

Fig. S9. RBD sequence alignment of MERS-CoV, GD-BatCoV, SE-PangolinCoV, and HKU4-BatCoV. Amino acids in the binding interfaces of MERS-CoV, GD-BatCoV, SE-PangolinCoV, and HKU4-BatCoV are colored grey, magenta, blue, and tan, respectively. The locations of tested mutations in the GD-BatCoV RBD are highlighted above the sequence, while those in the SE-PangolinCoV RBD are highlighted below the sequence. Relating to Fig. 2.

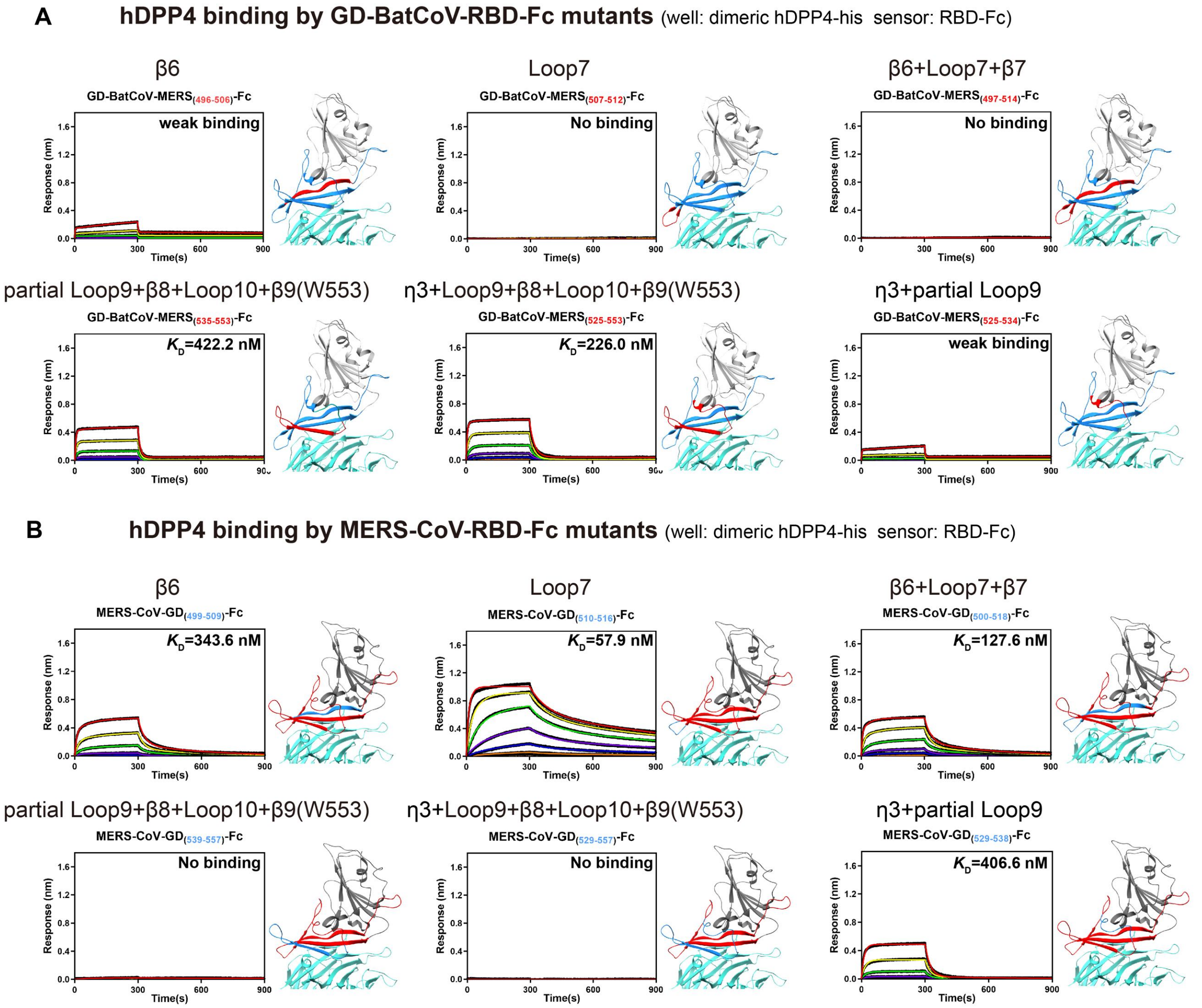

Fig. S10. Binding of hDPP4 by GD-BatCoV and MERS-CoV RBM fragment-swap mutants. **(A)** Effects of different RBM fragments from MERS-CoV on the binding of GD-BatCoV-RBD to hDPP4. **(B)** Effect of different RBM fragments from GD-BatCoV on the binding of MERS-CoV-RBD to hDPP4. The locations of RBM fragments are annotated above the binding results and the structural models of the RBM swapping are shown next to the binding results. The RBD core domain is colored grey and hDPP4 is colored cyan. RBM regions of GD-BatCoV-RBD and MERS-CoV-RBD are colored blue and red, respectively. The RBD-Fc proteins were loaded onto Protein A biosensors and dipped into the wells containing hDPP4-his protein with concentrations ranging from 800 nM to 1.1 nM in a three-fold serial dilution. K_D values are shown alongside the binding curves. Binding kinetic parameters are summarized in table S6.

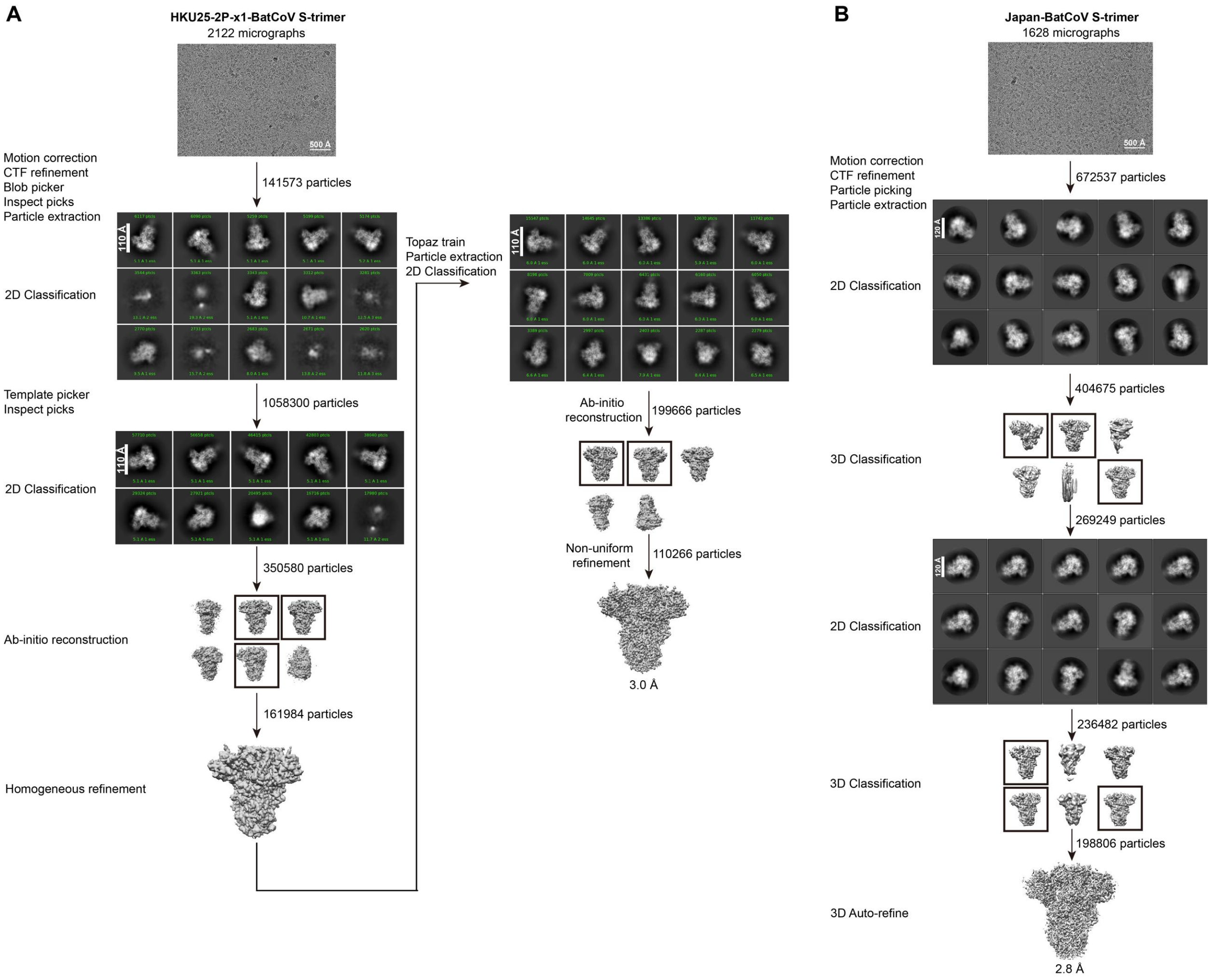

Fig. S11. Cryo-EM data processing flow-chart for the structures of HKU25-BatCoV and Japan-BatCoV S-trimers. (A) Data processing pipeline for the HKU25-BatCoV S-trimer dataset. After two rounds of 2D classification, ab-initio reconstruction and homogeneous refinement were performed. High-quality particles were used for topaz train and particle re-picking, before additional 2D classification and ab-initio reconstruction procedures were performed to eliminate poor-quality particles. Non-uniform refinement revealed a single conformation for the HKU25-BatCoV S-trimer. **(B)** Data processing pipeline for the Japan-BatCoV dataset. 2D and 3D classification steps were performed to remove contaminating particles. Only one conformation was identified for the Japan-BatCoV S-trimer.

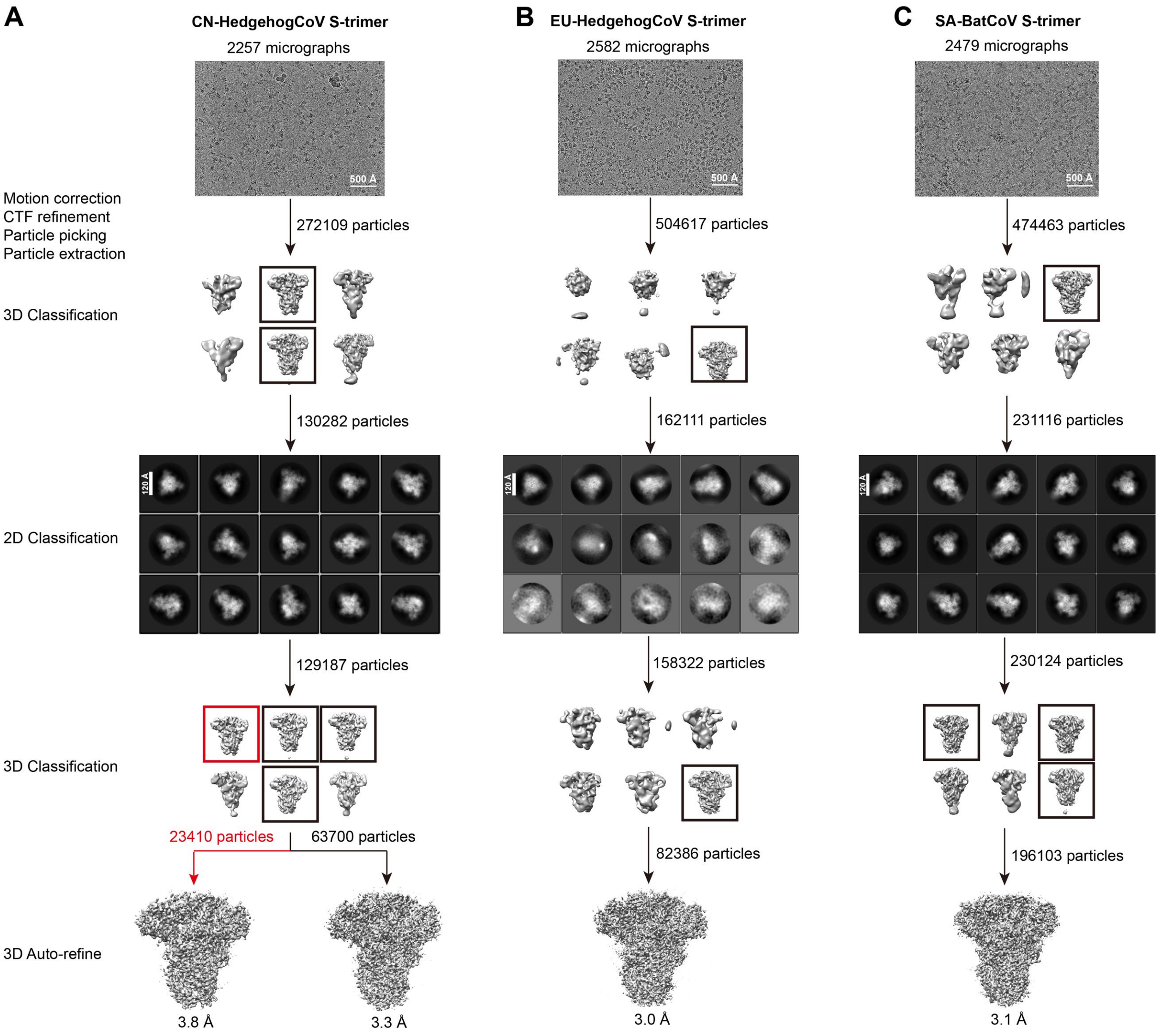

Fig. S12. Cryo-EM data processing flow-chart for the CN-HedgehogCoV, EU-HedgehogCoV, and SA-BatCoV S-trimer structures. 3D and 2D classification steps were performed to remove contaminating particles. For the CN-HedgehogCoV S-trimer, two conformations were identified. Only one conformation was identified during the 3D classification for both the EU-HedgehogCoV and SA-BatCoV S-trimer structures.

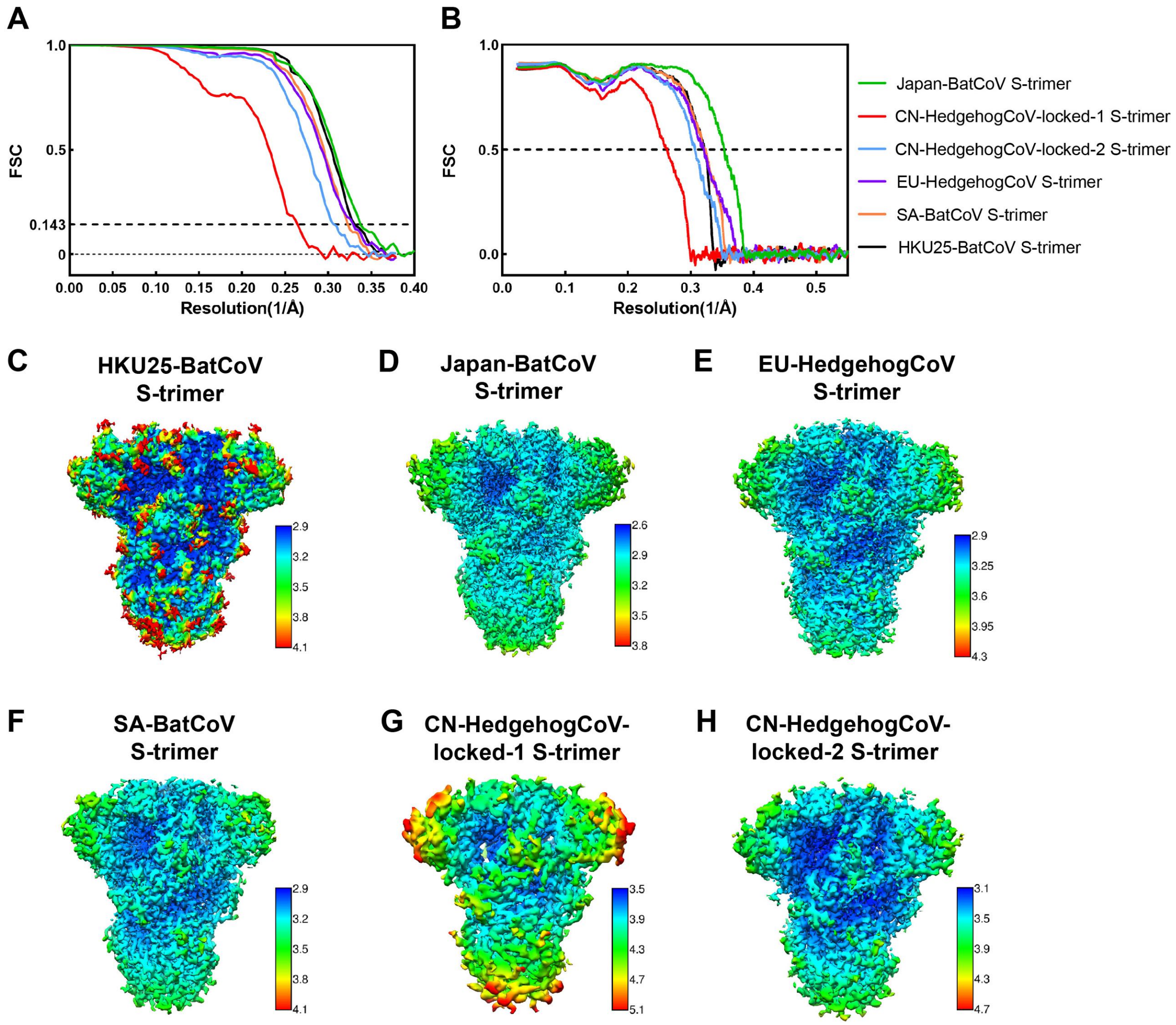

Fig. S13. Resolution assessments for the HKU25-BatCoV, Japan-BatCoV, EU-HedgehogCoV, SA-BatCoV, CN-HedgehogCoV-locked-1, and CN-HedgehogCoV-locked-2 S-trimer structures. (A) Global resolution assessment by Fourier shell correlation at the 0.143 criterion. **(B)** Correlations between model and map using Fourier shell correlation at the 0.5 criterion. **(C-H)** Local resolution maps for the HKU25-BatCoV, Japan-BatCoV, EU-HedgehogCoV, SA-BatCoV, CN-HedgehogCoV-locked-1, and CN-HedgehogCoV-locked-2 S-trimer structures.

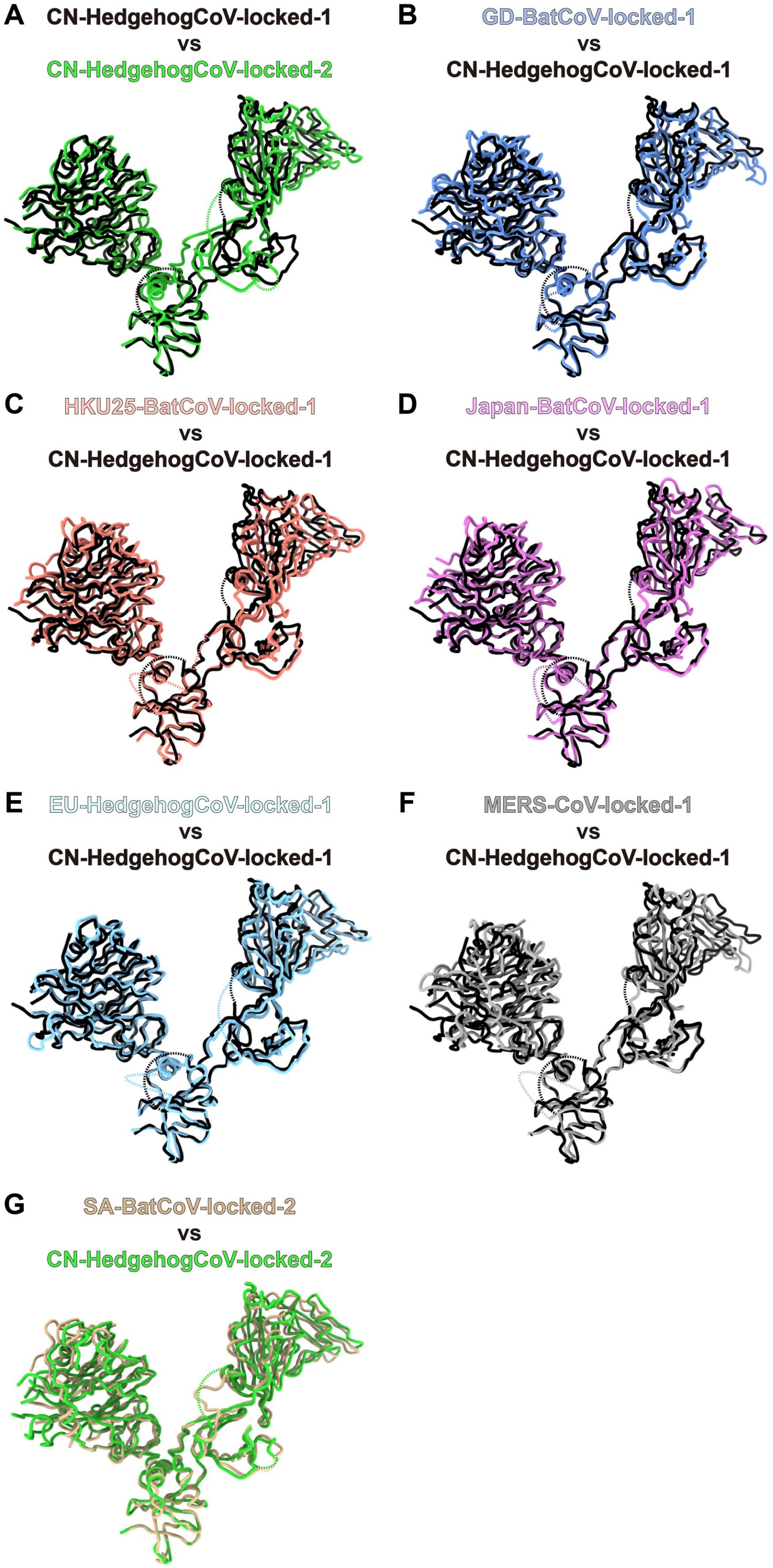
A
CN-HedgehogCoV-locked-1
vs
CN-HedgehogCoV-locked-2
B
GD-BatCoV-locked-1
vs
CN-HedgehogCoV-locked-1
C
HKU25-BatCoV-locked-1
vs
CN-HedgehogCoV-locked-1
D
Japan-BatCoV-locked-1
vs
CN-HedgehogCoV-locked-1
E
EU-HedgehogCoV-locked-1
vs
CN-HedgehogCoV-locked-1
F
MERS-CoV-locked-1
vs
CN-HedgehogCoV-locked-1
G
SA-BatCoV-locked-2
vs
CN-HedgehogCoV-locked-2

Fig. S14. Comparison of the S1 structures of the locked-1 and locked-2 S-trimer structures determined in this study. (A) Structural alignment of S1 domains of the CN-HedgehogCoV S-trimer in locked-1 (black) and locked-2 (green) conformations. **(B-F)** The S1 structures of GD-BatCoV-locked-1, HKU25-BatCoV-locked-1, Japan-BatCoV-locked-1, EU-HedgehogCoV-locked-1, and MERS-CoV-locked-1 are aligned with the S1 structure of CN-HedgehogCoV-locked-1 S-trimer. **(G)** Structural alignment of the S1 domains of the CN-HedgehogCoV-locked-2 and SA-BatCoV-locked-2 S-trimers.

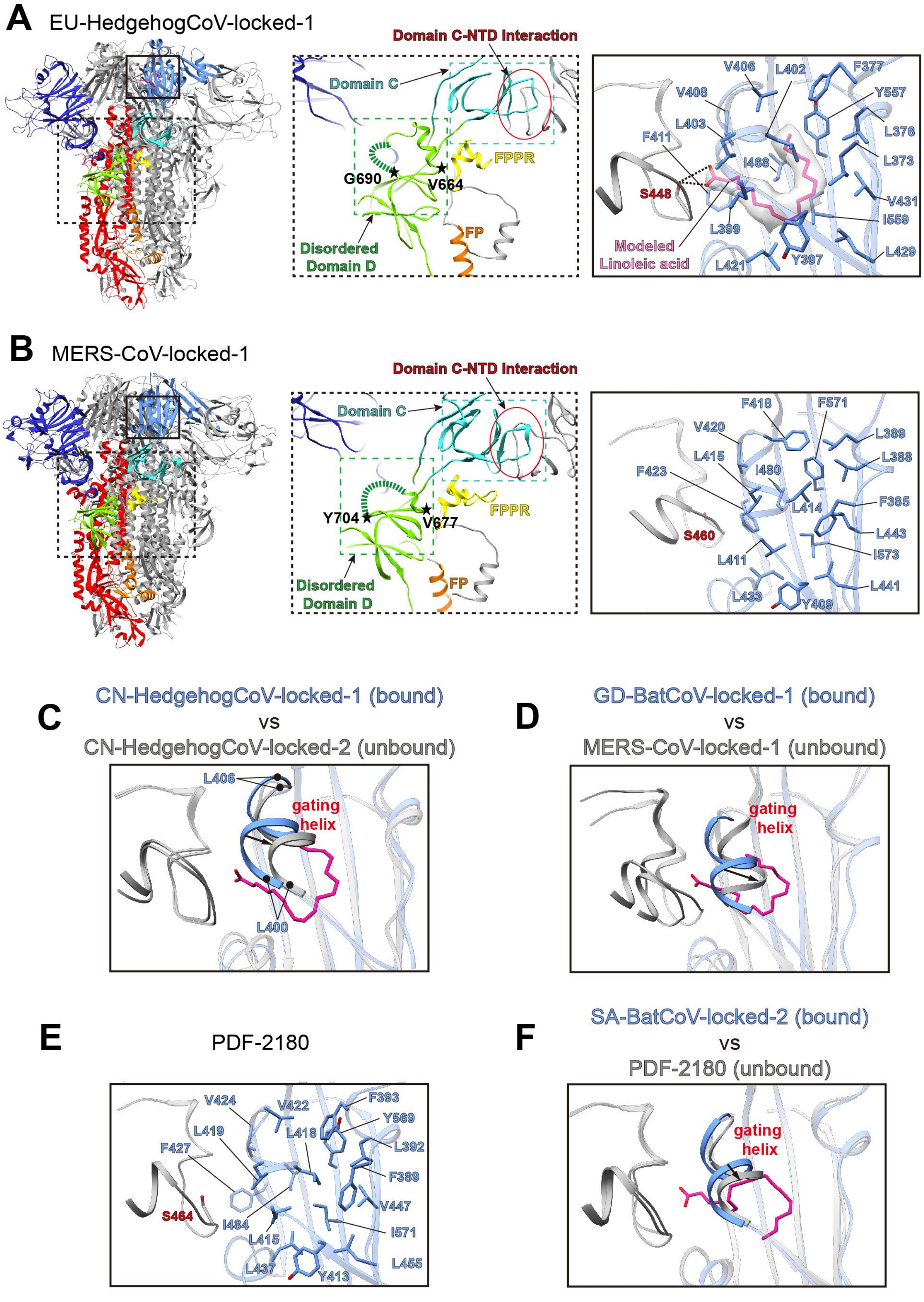

A
EU-HedgehogCoV-locked-1
Domain C-NTD Interaction
Domain C
FPPR
G690
V664
FP
Disordered Domain D
V406
L402
F377
V408
Y557
L403
L376
F411
I468
L373
S448
V431
L399
I559
Modeled Linoleic acid
L421
Y397
L429
B
MERS-CoV-locked-1
Domain C-NTD Interaction
Domain C
FPPR
Y704
V677
FP
Disordered Domain D
F418
F571
V420
L389
L415
L388
F423
I480
L414
F385
S460
L443
L411
I573
L433
Y409
L441
C
CN-HedgehogCoV-locked-1 (bound)
vs
CN-HedgehogCoV-locked-2 (unbound)
L406
gating helix
L400
D
GD-BatCoV-locked-1 (bound)
vs
MERS-CoV-locked-1 (unbound)
gating helix
E
PDF-2180
V424
V422
F393
Y569
L419
L418
L392
F427
F389
V447
S464
I484
I571
L415
L437
Y413
L455
F
SA-BatCoV-locked-2 (bound)
vs
PDF-2180 (unbound)
gating helix

Fig. S15. Cryo-EM structure of the EU-HedgehogCoV S-trimer and comparison of merbecovirus S-RBD fatty acid-binding pockets. (A) The left panel shows a molecular model of the EU-HedgehogCoV S-trimer; The middle panel shows a zoom-in view of Domains C and D with dashed boxes highlighting their positions; The red circle highlights interactions between Domain C and the adjacent NTD; The right panel shows the fatty acid-binding pocket within the EU-HedgehogCoV S-RBD. Hydrophobic amino acid sidechains lining the fatty acid-binding pockets are shown as sticks. Structural elements from a neighboring RBD that form the fatty acid-binding pocket are shown in grey, with fatty acid-interacting residues labeled in red. **(B)** The structural model and structural features of the MERS-CoV S-trimer are shown for comparison. **(C)** Overlay of the fatty acid-bound and -unbound RBD fatty acid-binding pockets in the CN-HedgehogCoV-locked-1 (blue) and CN-HedgehogCoV-locked-2 (grey) S-trimer structures. The inward shift of the fatty acid-binding pocket gating helix ($400\text{-}406_{CN}$) is indicated by a black arrow. **(D)** Overlay of fatty acid-bound and -unbound RBD fatty acid-binding pockets in the GD-BatCoV-locked-1 (blue) and MERS-CoV (grey) S-trimer structures. **(E)** Structure of the unbound RBD fatty acid-binding pocket of PDF-2180 S-trimer (PDB:7U6R). **(F)** An overlay of fatty acid-bound and -unbound RBD fatty acid-binding pockets from SA-BatCoV-locked-2 (blue) and PDF-2180 (grey) S-trimer structures. The inward shift of the fatty acid-binding pocket gating helix is also indicated by a black arrow.

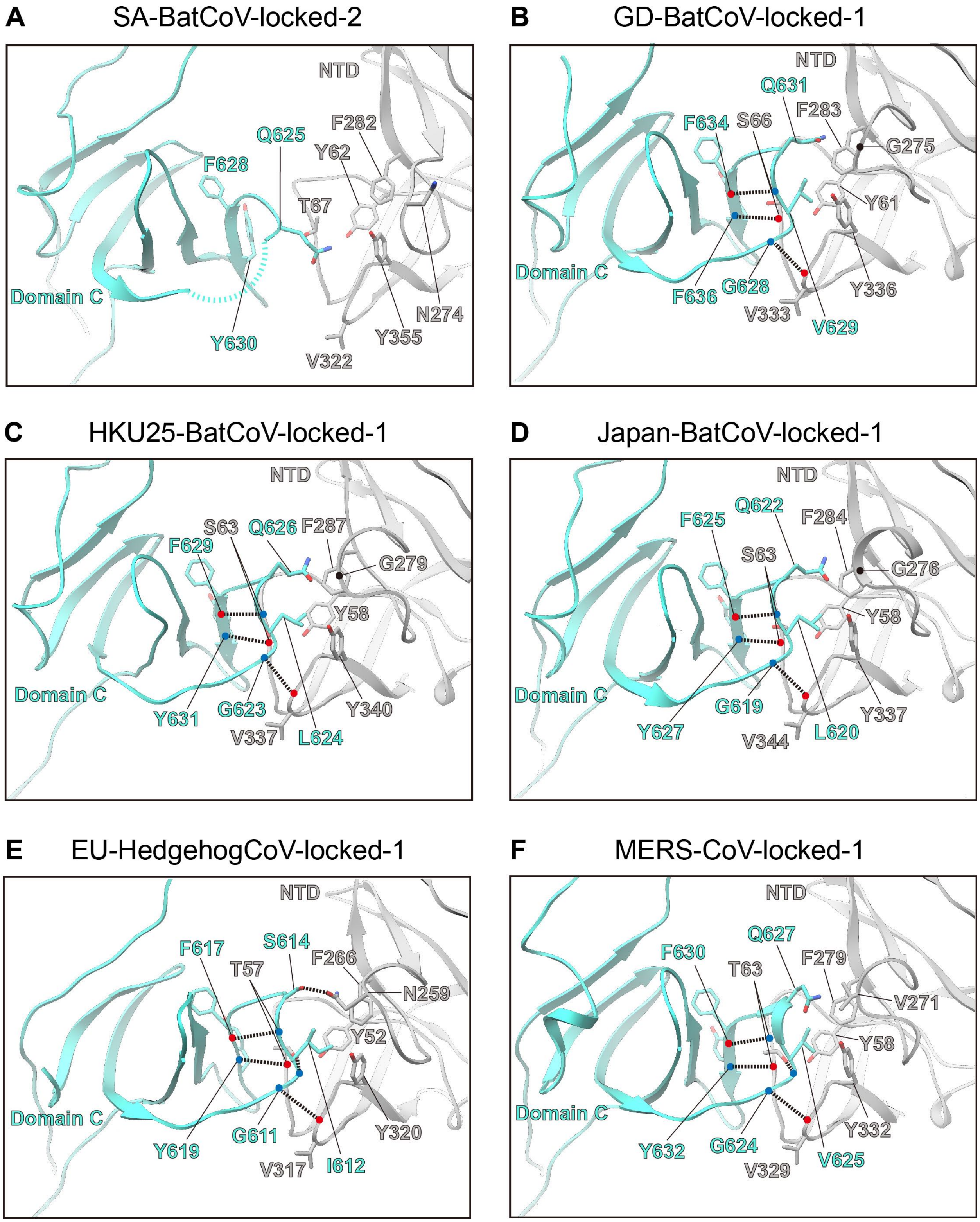

Fig. S16. Domain C-NTD interactions among merbecovirus S-trimer structures. (A-F) Polar and hydrophobic interactions between Domain C regions and their respective adjacent NTDs in the structures of SA-BatCoV-locked-2, GD-BatCoV-locked-1, HKU25-BatCoV-locked-1, Japan-

BatCoV-locked-1, EU-HedgehogCoV-locked-1, and MERS-CoV-locked-1 S-trimers. Each Domain C and its adjacent NTD are colored cyan and grey, respectively. Dashed lines represent hydrogen bonds; blue and red dots indicate backbone amide and carbonyl atoms, respectively.

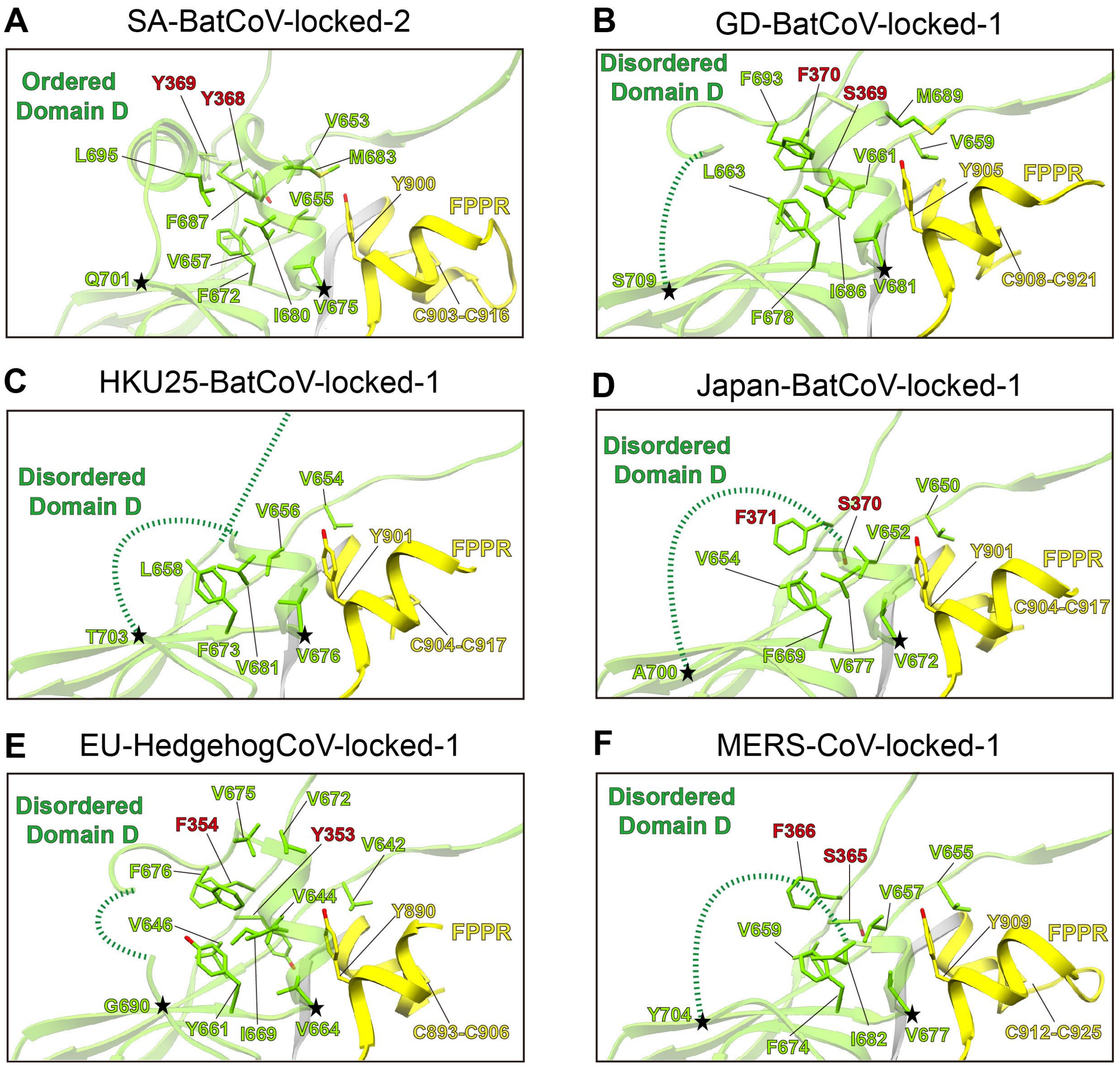

Fig. S17. Detailed structural features of Domain D regions in the merbecovirus S-trimers. (A-F) The Domain D hydrophobic cores are shown for SA-BatCoV-locked-2, GD-BatCoV-locked-1, HKU25-BatCoV-locked-1, Japan-BatCoV-locked-1, EU-HedgehogCoV-locked-1, and MERS-CoV-locked-1 S-trimers. The start and end of the Domain D-loop region are highlighted by black star symbols. $Y354_{CN}$ and $F355_{CN}$ are shown to engage in different interactions between the locked-1 and locked-2 CN-HedgehogCoV S-trimer (relating to Fig. 5). Residues in the shown S-trimers corresponding to $Y354_{CN}$ and $F355_{CN}$ of CN-HedgehogCoV S-protein are colored red to highlight their varied interactions. Disordered regions within Domain D are represented by green dashed lines.

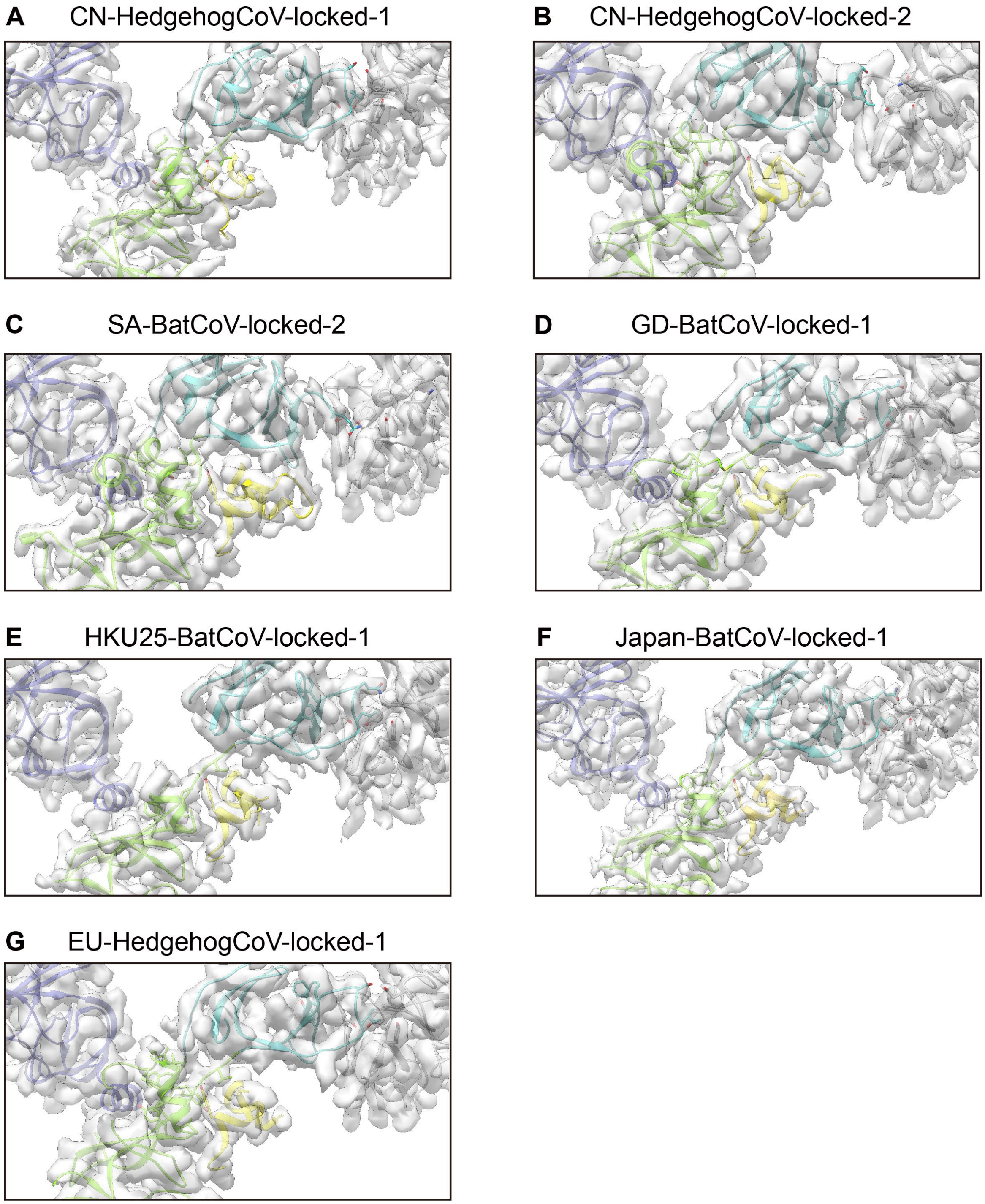

Fig. S18. Cryo-EM densities for the regions near Domain C and Domain D in the determined merbecovirus S-trimers. (A-G) The colors of different domains are based on Fig.4 (NTD in blue, Domain C in cyan and Domain D in green). Residues involved in interactions correspond to Fig.5 and figs. S16-17.

A

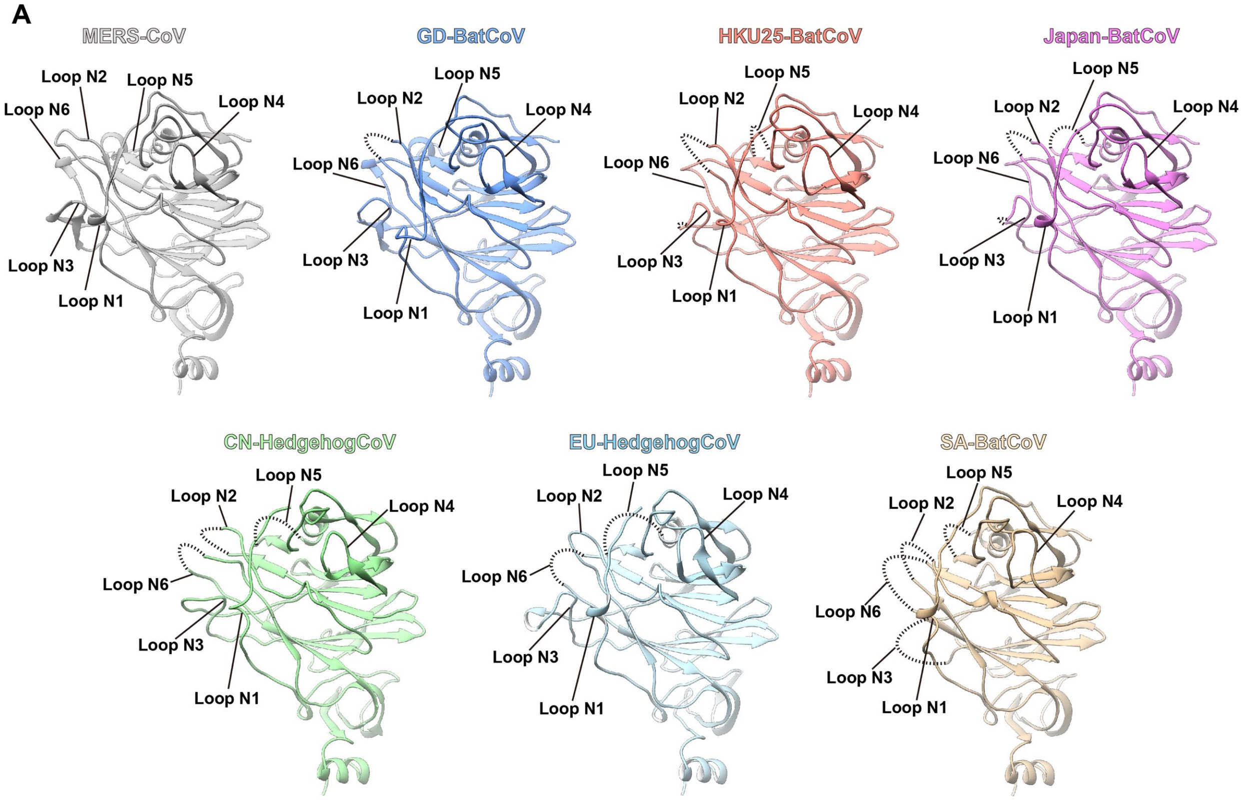

B

```
                1        10        20            30       40        50        60        70
MERS-CoV        MIHSVFLLMFLLTPTESYVD---VGPDSVKSACIEVDIQ---QTFFDKTWPRPIDVSKADGIIYPQGRTYSNITITYQGLFPYQ
GD-BatCoV       MRLSVCLLMFLLTPIKGDVD---SGPPSSATSCKEADMRNSSSEFFNKQWPMPINASKADGIIYPTGKSYSNISLTLQGLFPKH
HKU25-BatCoV    MTLSMFLLMFLLTHIKGDVD---LGPDSSAQSCQEVDIQ---PQYFQKDWTKPIDPAKADGIIYPTGRSYSNITLSYQGLFPRQ
Japan-BatCoV    MTHLMFLLMFLLTPIRGDVD---LGPDSTRDTCDEVVVQ---VSAFQKDWPQPINPLFADGIIYPTGRSYSNISLSYQGLFPRQ
CN-HedgehogCoV  MIRSVFLLMCLLTLIKS-----------GDANCIVVDMQ---PSYFQRDWPRPINMSAADGIIYPVGRTYANITIMLSGLFPHQ
EU-HedghogCoV   MIRSACLLMCLLMFIKATP---------SEGTCISVDMQ---PSYFIKNWSMPINMSKADGVLYPVGRTYSNITITLEGLFPHQ
SA-BatCoV       MTYSVFPLMCLLTFIGANAKIVTLPGNDATGYCPSVDMQP--SYFIQHNWPEPIDMNKADGVIYPNGRTYSNITLQTTNLFPKN
```

NTD N-terminal extension

Loop N1: 36-40

```
                 80        90         100       110       120       130       140       150
MERS-CoV        GDHGDMYVYSAGHATGTT-------PQKLFVANYSQDVKQFANGFVVRIGAAANSTGTVIISPST--SATIRKIYPAFMLGSSV
GD-BatCoV       GDLGEQYIYSVGHSDSNY-----DYLGKLFVSDYATKVVPFNNGFVVRIGAAANATGSVIISTV---QKTIKKIYPAFMLGSSV
HKU25-BatCoV    GDQGQLYLYSVSHADPNTGKMGPDGIGKLFISNYTNETNTFDNGFVVRIGDAANSSYPIIISPQG-PARTIKKIYPAFMLGAYI
Japan-BatCoV    GDLGRQYIYSVTHAGDS-TLYPPQGIGKHFISNYSAETLPFGNGFVVRIGFSANKTGSLVISPTT--SKVIKKIYPAFMLGTAV
CN-HedgehogCoV  ADKGKQYIYSMYHMKFN------QGVNDPFISNYSYYTEPFDNGFVVRIGANSNITGSSVVGSA---NATVKKIYPALMLGSVV
EU-HedghogCoV   ADRGDMYVYSQSH------------KGRPFISNYSLVTNDFGNGIVIRIGSAANKTGSPIISNIASNFQIMKKLYPALLFGSAV
SA-BatCoV       GDLGTQYVYSASNHKST--------ANDAFISNYSYYGNPFGDGIVIRIGQNANKTGSVIVGQA---QTTMKKIYPALMLGSSF
```

Loop N2: 89-100

Loop N3: 131-139

```
                     160       170       180       190       200       210       220       230
MERS-CoV        GNFSD-GKMGRFFNHTLVLLPDGCGTLLRAFYCILEPRSGNHCPAGNSYTSFATYHTPATDCSDGNYNRNASLNSFKEYFNLRN
GD-BatCoV       GNFSN-GVSGRYFNHTLLLLPDGCGTRLWALYCVIEPRNGSYCPGNSNYNTFAVFDTPHTDCTSAGYNTNATLNSFKEYFDLQN
HKU25-BatCoV    GNFTN-NRTVRYLNHTLVILPEGCGTSLSVFYCVLIPRTQHRCPEDNAYSSYVIWETPHQDCEGSTVNRNISLNAFKHYFNLTQ
Japan-BatCoV    TSFAH-GHAGRYLNHTLVILPESCGTSLTVFYCILEPRNGTRCPNHQDYTSYVIWETPHLDCG-SSVNRNATLNSFKVSFNLRQ
CN-HedgehogCoV  GKFPS-NKTGRYFNHTLVILPEKCGTQITALYCVLQPKNQTNCTGHTTYTSYVLVENKTCSTDAD----GNLKQSLQTWFDLKD
EU-HedghogCoV   GKFPANNKTGAYFNHSLVILPEKCGTVITALYCVLEPKNDTNCTGSSGYVSYVLFENFSCDATTD----GNLKNSLQQWFNIKD
SA-BatCoV       GNFSANNKSGAYFNHTLLILPSKCGTVFQVAYCLLQPRTESKCPGNSNYVSYFLADSPSDCSSTSDEIRRNGLRDIRKFFNLVN
```

Loop N4: 156-160

Loop N5: 209-222

```
                  240       250        260       270       280       290       300       310
MERS-CoV        CTFMYTYNITEDEILEWFGITQTAQGVHLFSSRYVDLYGGNMFQFATLPVYDTIKYYSIIPHSIRS-IQSDRKAWAAFYVYKLQ
GD-BatCoV       CSFIYSFNITEDENAEWFGITQNTQGVHLYSSRKGDLYGSNMFLFATLPVYDGIKYYTVIPRSIRS-KYSERQAWAAFYIYSLH
HKU25-BatCoV    CKFFYSYNITEDEHAEWFGITQDARGVHLYSSRKGDLYGGNMFLFASLPIYDTLKYYTVIPRSIRS-KYNSRQGWAAFYIYKLH
Japan-BatCoV    CKFMRTYNISDDENQEWFGITQNAQGVHLYSSRKGDLYGGNMFLFDTLPVYETLKYYTVIPRSLRS-RLNERQAWAAFYVYSLH
CN-HedgehogCoV  CLFEKKYNVSADEREEWFGITQDQQGVHLYTSRKNGFKSNNMFLFATLPIYERLRYYTVMPRSIKTTANNDHMAWSAFYIYQLH
EU-HedghogCoV   CLFEKNYNVTDDEREEWFGIIQDQQGVHLYTSRKNGYS-NNMFLFATLPIYDQILYYTVMPRSINASNYASYHAFSAFYIYKLH
SA-BatCoV       CTYFEEFNVTADERAEWFGIVQDAQGVHLYTSRKNGFNSNNLFLFATVPIYDKLNYYTVIPRSVIT-PSNQRDAWAAFYIYPLH
```

Loop N6: 301-311

Fig. S19. Structural features and sequences of NTDs in the determined merbecovirus S-trimers. (A) Structural features of NTDs in merbecovirus S-trimers. The locations of Loop-N1 to Loop-N6 are indicated. **(B)** An alignment of studied merbecovirus NTD amino acid sequences. The positions of Loop-N1 to Loop-N6 are marked in the sequences. The putative sialic acid binding pocket residues are highlighted within black boxes with key sialic acid interacting residues highlighted within red boxes.

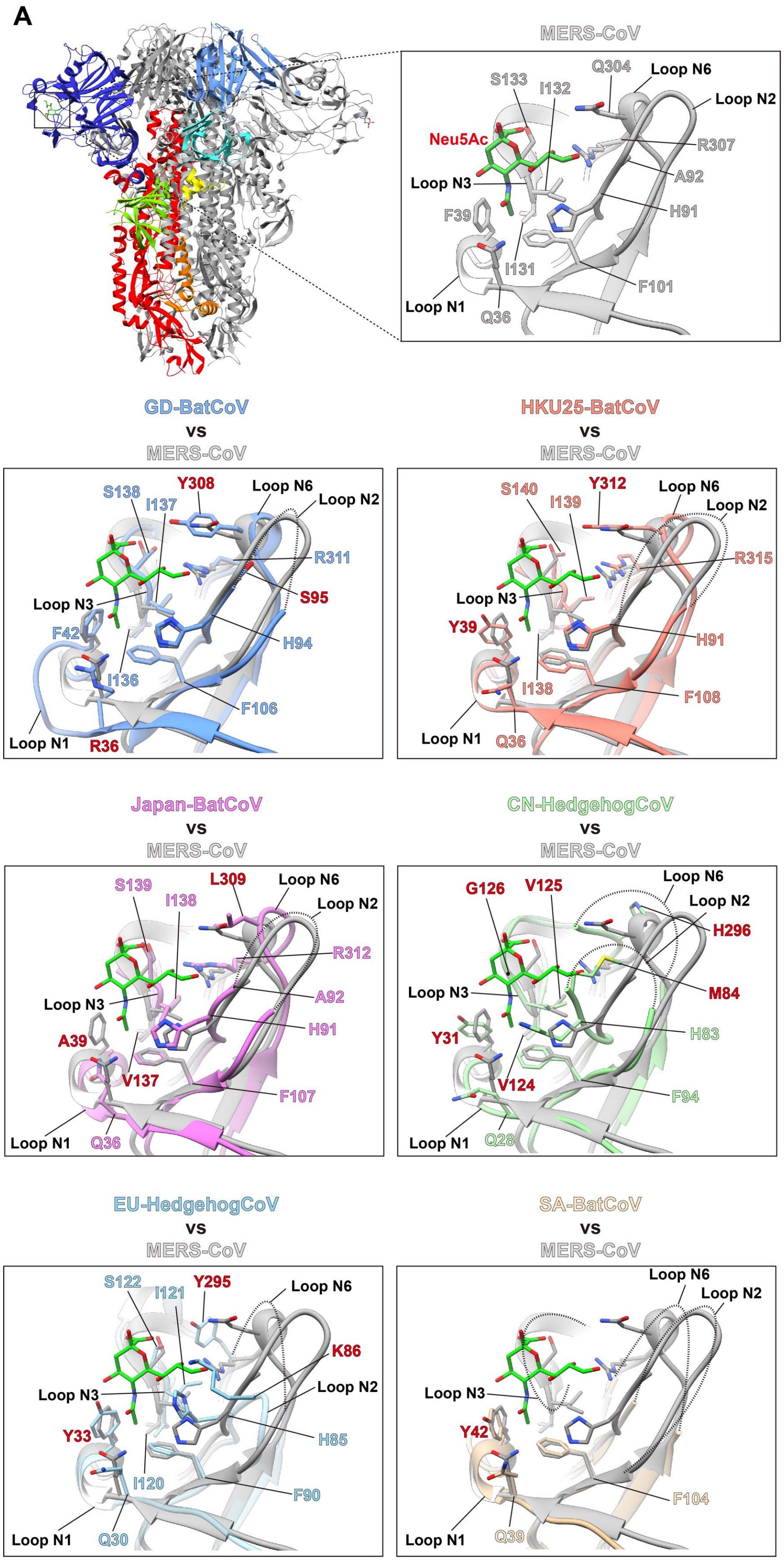

A
MERS-CoV
S133
I132
Q304
Loop N6
Loop N2
Neu5Ac
R307
A92
Loop N3
H91
F39
I131
F101
Loop N1
Q36
GD-BatCoV
vs
MERS-CoV
S138
I137
Y308
Loop N6
Loop N2
R311
S95
Loop N3
F42
H94
I136
F106
Loop N1
R36
HKU25-BatCoV
vs
MERS-CoV
S140
I139
Y312
Loop N6
Loop N2
R315
Loop N3
Y39
H91
I138
F108
Loop N1
Q36
Japan-BatCoV
vs
MERS-CoV
S139
I138
L309
Loop N6
Loop N2
R312
A92
Loop N3
H91
A39
V137
F107
Loop N1
Q36
CN-HedgehogCoV
vs
MERS-CoV
G126
V125
Loop N6
Loop N2
H296
Loop N3
M84
H83
Y31
V124
F94
Loop N1
Q28
EU-HedgehogCoV
vs
MERS-CoV
S122
I121
Y295
Loop N6
K86
Loop N2
Loop N3
H85
Y33
I120
F90
Loop N1
Q30
SA-BatCoV
vs
MERS-CoV
Loop N6
Loop N2
Loop N3
Y42
F104
Loop N1
Q39

Fig. S20. Sialic acid binding pocket in the NTDs of merbecovirus S-trimers. Putative sialic acid binding pockets in the NTDs of GD-BatCoV, HKU25-BatCoV, Japan-BatCoV, CN-HedgehogCoV, EU-HedgehogCoV, and SA-BatCoV S-trimers are shown and aligned with the MERS-CoV NTD sialic acid binding pocket (grey). Amino acids forming the sialic acid binding pocket are shown as sticks (colored) and compared to corresponding MERS-CoV sialic acid binding pocket residues (grey sticks). The Neu5Ac bound in the MERS-CoV sialic acid binding pocket is depicted in green. Non-conservative sialic acid binding pocket residues by comparison with MERS-CoV are labeled in red.

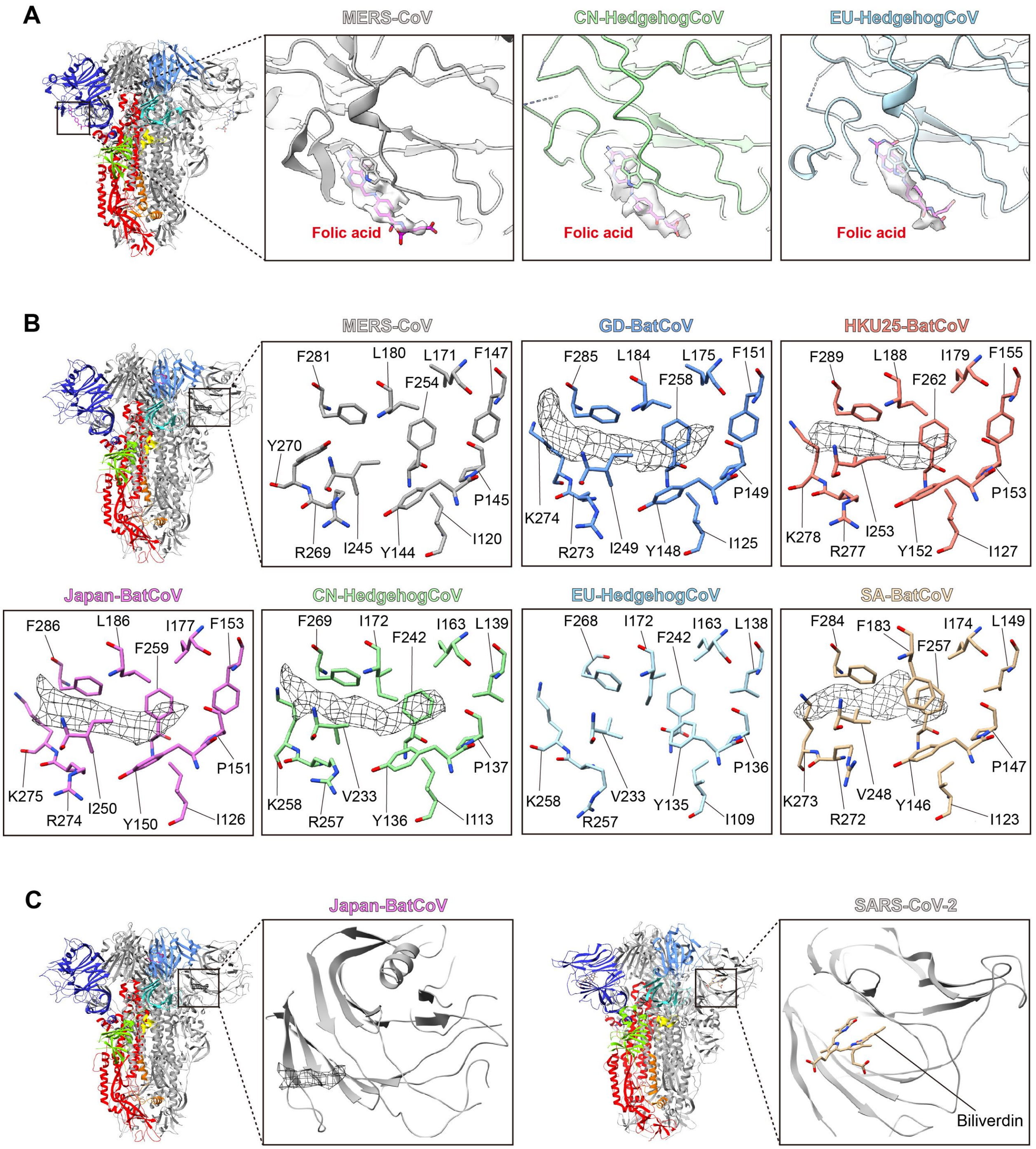

Fig. S21. Other small molecule binding pockets in the NTDs of merbecovirus S-trimers. (A) Binding of small molecule densities compatible with the structure of folic acid in the NTDs of CN-HedgehogCoV and EU-HedgehogCoV S-trimers. The structure of MERS-CoV NTD folic acid binding pocket (PDB:6Q04) is shown for comparison. **(B)** Unknown densities in the NTDs of studied merbecovirus S-trimers. Residues lining the binding pockets for the unknown molecule within different merbecovirus S-trimers are represented as sticks. The binding pocket residues are mostly hydrophobic. No small molecule density was observed within the hydrophobic NTD pockets of the MERS-CoV and EU-HedgehogCoV S-trimers. The lack of the small molecule

density in the EU-HedgehogCoV S-trimer could be attributed to the orientation of the residue $Y135_{EU}$ sidechain, which occupies the pocket and corresponds to $Y148_{GD}$ in GD-BatCoV S-protein. The binding site residues are mostly conserved in MERS-CoV except for $Y270_{MERS}$ (corresponding to $K274_{GD}$ in GD-BatCoV S-protein), which occupies the pocket. **(C)** The hydrophobic binding pocket for the unknown molecule is reminiscent of the biliverdin binding pocket within the NTD of the SARS-CoV-2 S-protein in terms of location.

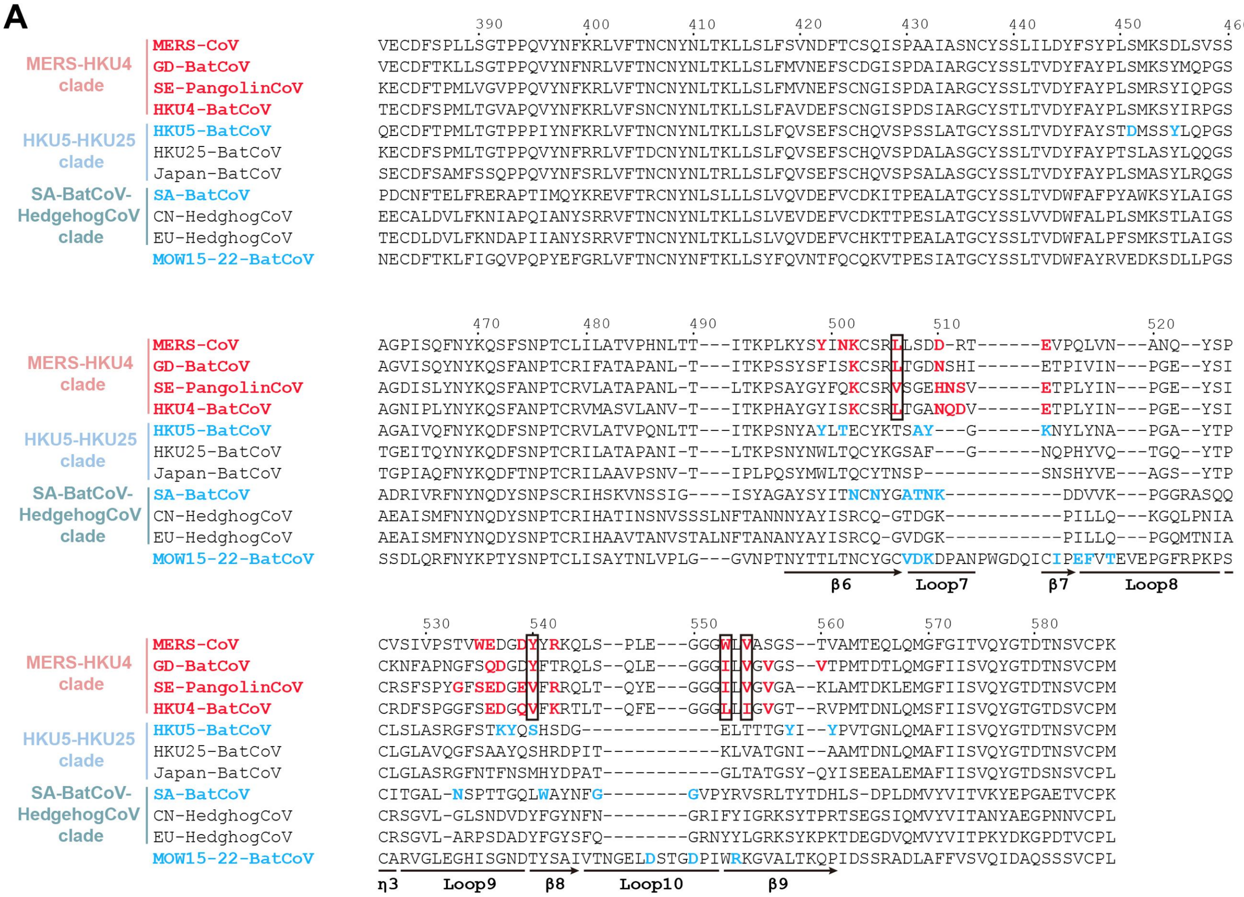

Fig. S22. An alignment of representative merbecovirus RBD sequences from different clades. Names of hDPP4-binding virus sequences are shown in red, with their hDPP4 binding residues highlighted in red. Names of ACE2-binding virus sequences are shown in blue, with their ACE2 binding residues highlighted in blue. Relating to Fig. 6B.

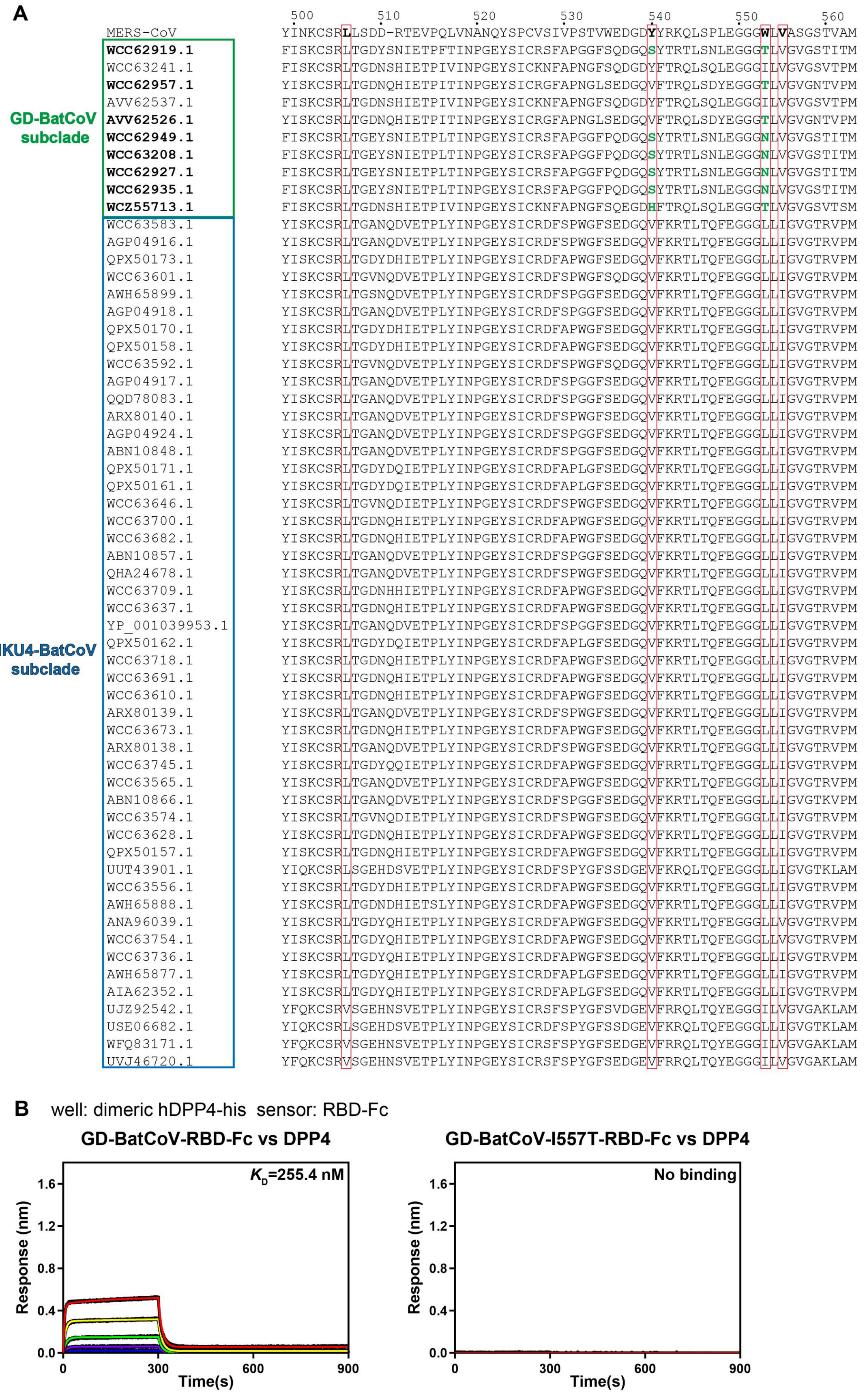
A
500
510
520
530
540
550
560
MERS-CoV YINKCSRLLSDD-RTEVPQLVNANQYSPCVSIVPSTVWEDGDYYRKQLSPLEGGGWLVASGSTVAM
GD-BatCoV subclade
WCC62919.1 FISKCSRLTGDYSNIETPFTINPGEYSICRSFAPGGFSQDGQSYTRTLSNLEGGGTLVGVGSTITM
WCC63241.1 FISKCSRLTGDNSHIETPIVINPGEYSICKNFAPNGFSQDGDYFTRQLSQLEGGGILVGVGSVTPM
WCC62957.1 YISKCSRLTGDNQHIETPITINPGEYSICRGFAPNGLSEDGQVFTRQLSDYEGGGTLVGVGNTVPM
AVV62537.1 FISKCSRLTGDNSHIETPIVINPGEYSICKNFAPNGFSQDGDYFTRQLSQLEGGGILVGVGSVTPM
AVV62526.1 YISKCSRLTGDNQHIETPITINPGEYSICRGFAPNGLSEDGQVFTRQLSDYEGGGTLVGVGNTVPM
WCC62949.1 FISKCSRLTGEYSNIETPLTINPGEYSICRSFAPGGFPQDGQSYTRTLSNLEGGGNLVGVGSTITM
WCC63208.1 FISKCSRLTGEYSNIETPLTINPGEYSICRSFAPGGFPQDGQSYTRTLSNLEGGGNLVGVGSTITM
WCC62927.1 FISKCSRLTGEYSNIETPLTINPGEYSICRSFAPGGFPQDGQSYTRTLSNLEGGGNLVGVGSTITM
WCC62935.1 FISKCSRLTGEYSNIETPLTINPGEYSICRSFAPGGFPQDGQSYTRTLSNLEGGGNLVGVGSTITM
WCZ55713.1 FISKCSRLTGDNSHIETPIVINPGEYSICKNFAPNGFSQEGDHFTRQLSQLEGGGTLVGVGSVTSM
HKU4-BatCoV subclade
WCC63583.1 YISKCSRLTGANQDVETPLYINPGEYSICRDFSPWGFSEDGQVFKRTLTQFEGGGLLIGVGTRVPM
AGP04916.1 YISKCSRLTGANQDVETPLYINPGEYSICRDFSPWGFSEDGQVFKRTLTQFEGGGLLIGVGTRVPM
QPX50173.1 YISKCSRLTGDYDHIETPLYINPGEYSICRDFAPWGFSEDGQVFKRTLTQFEGGGLLIGVGTRVPM
WCC63601.1 YISKCSRLTGVNQDVETPLYINPGEYSICRDFSPWGFSQDGQVFKRTLTQFEGGGLLIGVGTRVPM
AWH65899.1 YISKCSRLTGSNQDVETPLYINPGEYSICRDFSPGGFSEDGQVFKRTLTQFEGGGLLIGVGTRVPM
AGP04918.1 YISKCSRLTGANQDVETPLYINPGEYSICRDFSPGGFSEDGQVFKRTLTQFEGGGLLIGVGTRVPM
QPX50170.1 YISKCSRLTGDYDHIETPLYINPGEYSICRDFAPWGFSEDGQVFKRTLTQFEGGGLLIGVGTRVPM
QPX50158.1 YISKCSRLTGDYDHIETPLYINPGEYSICRDFAPWGFSEDGQVFKRTLTQFEGGGLLIGVGTRVPM
WCC63592.1 YISKCSRLTGVNQDVETPLYINPGEYSICRDFSPWGFSQDGQVFKRTLTQFEGGGLLIGVGTRVPM
AGP04917.1 YISKCSRLTGANQDVETPLYINPGEYSICRDFSPGGFSEDGQVFKRTLTQFEGGGLLIGVGTRVPM
QQD78083.1 YISKCSRLTGANQDVETPLYINPGEYSICRDFSPGGFSEDGQVFKRTLTQFEGGGLLIGVGTRVPM
ARX80140.1 YISKCSRLTGANQDVETPLYINPGEYSICRDFSPWGFSEDGQVFKRTLTQFEGGGLLIGVGTRVPM
AGP04924.1 YISKCSRLTGANQDVETPLYINPGEYSICRDFSPGGFSEDGQVFKRTLTQFEGGGLLIGVGTRVPM
ABN10848.1 YISKCSRLTGANQDVETPLYINPGEYSICRDFSPGGFSEDGQVFKRTLTQFEGGGLLIGVGTRVPM
QPX50171.1 YISKCSRLTGDYDQIETPLYINPGEYSICRDFAPLGFSEDGQVFKRTLTQFEGGGLLIGVGTRVPM
QPX50161.1 YISKCSRLTGDYDQIETPLYINPGEYSICRDFAPLGFSEDGQVFKRTLTQFEGGGLLIGVGTRVPM
WCC63646.1 YISKCSRLTGVNQDIETPLYINPGEYSICRDFSPWGFSEDGQVFKRTLTQFEGGGLLIGVGTRVPM
WCC63700.1 YISKCSRLTGDNQHIETPLYINPGEYSICRDFAPWGFSEDGQVFKRTLTQFEGGGLLIGVGTRVPM
WCC63682.1 YISKCSRLTGDNQHIETPLYINPGEYSICRDFAPWGFSEDGQVFKRTLTQFEGGGLLIGVGTRVPM
ABN10857.1 YISKCSRLTGANQDVETPLYINPGEYSICRDFSPGGFSEDGQVFKRTLTQFEGGGLLIGVGTRVPM
QHA24678.1 YISKCSRLTGANQDVETPLYINPGEYSICRDFSPWGFSEDGQVFKRTLTQFEGGGLLIGVGTRVPM
WCC63709.1 YISKCSRLTGDNHHIETPLYINPGEYSICRDFAPWGFSEDGQVFKRTLTQFEGGGLLIGVGTRVPM
WCC63637.1 YISKCSRLTGDNQHIETPLYINPGEYSICRDFAPWGFSEDGQVFKRTLTQFEGGGLLIGVGTRVPM
YP_001039953.1 YISKCSRLTGANQDVETPLYINPGEYSICRDFSPGGFSEDGQVFKRTLTQFEGGGLLIGVGTRVPM
QPX50162.1 YISKCSRLTGDYDQIETPLYINPGEYSICRDFAPLGFSEDGQVFKRTLTQFEGGGLLIGVGTRVPM
WCC63718.1 YISKCSRLTGDNQHIETPLYINPGEYSICRDFAPWGFSEDGQVFKRTLTQFEGGGLLIGVGTRVPM
WCC63691.1 YISKCSRLTGDNQHIETPLYINPGEYSICRDFAPWGFSEDGQVFKRTLTQFEGGGLLIGVGTRVPM
WCC63610.1 YISKCSRLTGDNQHIETPLYINPGEYSICRDFAPWGFSEDGQVFKRTLTQFEGGGLLIGVGTRVPM
ARX80139.1 YISKCSRLTGANQDVETPLYINPGEYSICRDFSPWGFSEDGQVFKRTLTQFEGGGLLIGVGTRVPM
WCC63673.1 YISKCSRLTGDNQHIETPLYINPGEYSICRDFAPWGFSEDGQVFKRTLTQFEGGGLLIGVGTRVPM
ARX80138.1 YISKCSRLTGANQDVETPLYINPGEYSICRDFSPWGFSEDGQVFKRTLTQFEGGGLLIGVGTRVPM
WCC63745.1 YISKCSRLTGDYQQIETPLYINPGEYSICRDFAPWGFSEDGQVFRRTLTQFEGGGLLIGVGTRVPM
WCC63565.1 YISKCSRLTGANQDVETPLYINPGEYSICRDFAPWGFSEDGQVFKRTLTQFEGGGLLIGVGTRVPM
ABN10866.1 YISKCSRLTGANQDVETPLYINPGEYSICRDFSPGGFSEDGQVFKRTLTQFEGGGLLIGVGTKVPM
WCC63574.1 YISKCSRLTGVNQDIETPLYINPGEYSICRDFSPWGFSEDGQVFKRTLTQFEGGGLLIGVGTRVPM
WCC63628.1 YISKCSRLTGDNQHIETPLYINPGEYSICRDFAPWGFSEDGQVFKRTLTQFEGGGLLIGVGTRVPM
QPX50157.1 YISKCSRLTGDNQHIETPLYINPGEYSICRDFAPWGFSEDGQVFKRTLTQFEGGGLLIGVGTRVPM
UUT43901.1 YIQKCSRLSGEHDSVETPLYINPGEYSICRDFSPYGFSSDGEVFKRQLTQFEGGGLLIGVGTKLAM
WCC63556.1 YISKCSRLTGDYDHIETPLYINPGEYSICRDFAPWGFSEDGQVFKRTLTQFEGGGLLIGVGTRVPM
AWH65888.1 YISKCSRLTGDNDHIETSLYINPGEYSICRDFAPWGFSEDGQVFKRTLTQFEGGGLLIGVGTRVPM
ANA96039.1 YISKCSRLTGDYQHIETPLYINPGEYSICRDFAPWGFSEDGQVFKRTLTQFEGGGLLVGVGTRVPM
WCC63754.1 YISKCSRLTGDYQHIETPLYINPGEYSICRDFAPWGFSEDGQVFKRTLTQFEGGGLLVGVGTRVPM
WCC63736.1 YISKCSRLTGDYQHIETPLYINPGEYSICRDFAPWGFSEDGQVFKRTLTQFEGGGLLIGVGTRVPM
AWH65877.1 YISKCSRLTGDYQHIETPLYINPGEYSICRDFAPLGFSEDGQVFKRTLTQFEGGGLLIGVGTRVPM
AIA62352.1 YISKCSRLTGDYQHIETPLYINPGEYSICRDFAPLGFSEDGQVFKRTLTQFEGGGLLIGVGTRVPM
UJZ92542.1 YFQKCSRVSGEHNSVETPLYINPGEYSICRSFSPYGFSVDGEVFRRQLTQYEGGGILVGVGAKLAM
USE06682.1 YIQKCSRLSGEHDSVETPLYINPGEYSICRDFSPYGFSSDGEVFKRQLTQFEGGGLLIGVGTKLAM
WFQ83171.1 YFQKCSRVSGEHNSVETPLYINPGEYSICRSFSPYGFSEDGEVFRRQLTQYEGGGILVGVGAKLAM
UVJ46720.1 YFQKCSRVSGEHNSVETPLYINPGEYSICRSFSPYGFSEDGEVFRRQLTQYEGGGILVGVGAKLAM
B
well: dimeric hDPP4-his sensor: RBD-Fc
GD-BatCoV-RBD-Fc vs DPP4
KD=255.4 nM
Response (nm)
Time(s)
0.0
0.4
0.8
1.2
1.6
0
300
600
900
GD-BatCoV-I557T-RBD-Fc vs DPP4
No binding
Response (nm)
Time(s)

Fig. S23. An amino acid sequence alignment of HKU4 clade RBMs. (A) An alignment of HKU4 clade RBM sequences. Residues forming the hDPP4 interacting hydrophobic cluster are highlighted in red boxes with residues in the MERS-CoV sequence shown in bold. The GD-BatCoV and HKU4-BatCoV subclade virus names are outlined by green and blue boxes, respectively. Substituted hydrophobic interaction cluster residues and the names of virus strains with hydrophobic interaction cluster substitutions are highlighted in bold. **(B)** Binding of hDPP4 by wildtype and $I557T_{GD}$ mutant of GD-BatCoV-RBD-Fc. The RBD-Fc proteins were loaded onto Protein A biosensors and dipped into the wells containing hDPP4-his protein with concentrations ranging from 800 nM to 1.1 nM in a three-fold serial dilution. K_D is shown alongside the binding curves. Binding kinetic parameters are summarized in table S8.

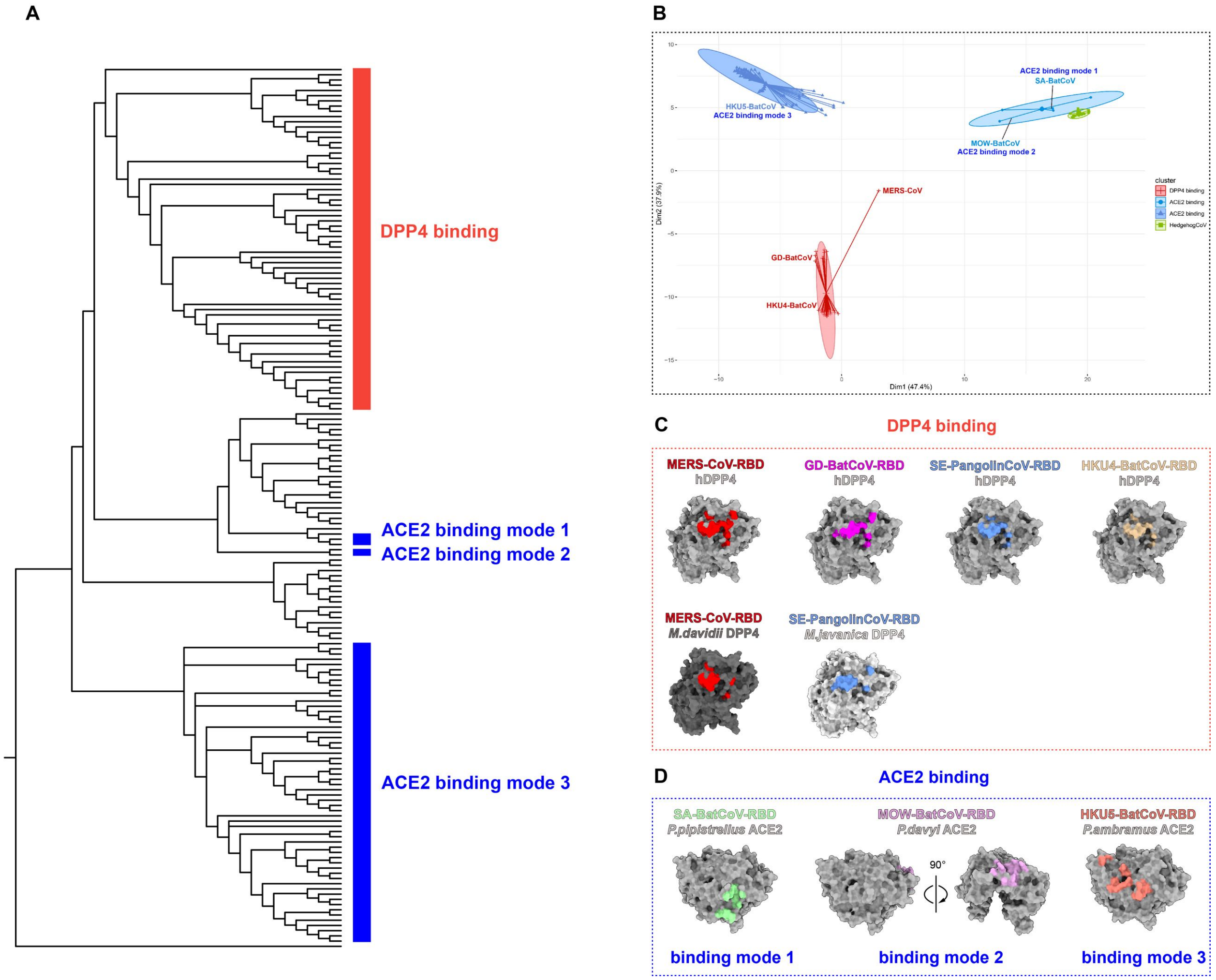

Fig. S24. Convergent DPP4-binding versus divergent ACE2-binding modes of merbecoviruses. (A) Phylogenetic tree based on an alignment of 168 merbecovirus RBD amino acid sequences. DPP4-binding merbecoviruses are colored red, while ACE2-binding merbecoviruses are colored blue. **(B)** Clustering analysis of 168 merbecovirus RBD amino acid sequences. DPP4-binding and ACE2-binding merbecoviruses are colored red and blue, respectively. **(C)** Footprints of MERS-CoV-RBD, GD-BatCoV-RBD, SE-PangolinCoV-RBD, HKU4-BatCoV-RBD on DPP4 molecules of different species (MERS-CoV-RBD: *M.davidii* DPP4 (PDB: 6L8Q), SE-PangolinCoV-RBD: *M.javanica* DPP4(PDB: 8ZE6)). **(D)** Footprints of SA-BatCoV-RBD (PDB: 7WPO), MOW-BatCoV-RBD (PDB: 9C6O), HKU5-BatCoV-RBD (PDB: 9D32) on ACE2 molecules of different species.

Table S1. Kinetic parameters of the interactions between hDPP4 and merbecovirus RBD-Fc proteins in BLI assays (relating to Fig. 1A)

	**MERS-RBD-Fc**			**GD-BatCoV-RBD-Fc**			**SE-PangolinCoV-RBD-Fc**			**HKU4-BatCoV-RBD-Fc**			**HKU25-BatCoV-RBD-Fc**		
	k_{on} ($M^{-1}S^{-1}$)	k_{off} (S^{-1})	K_D (nM)	k_{on} ($M^{-1}S^{-1}$)	k_{off} (S^{-1})	K_D (nM)	k_{on} ($M^{-1}S^{-1}$)	k_{off} (S^{-1})	K_D (nM)	k_{on} ($M^{-1}S^{-1}$)	k_{off} (S^{-1})	K_D (nM)	k_{on} ($M^{-1}S^{-1}$)	k_{off} (S^{-1})	K_D (nM)
**hDPP4**	1.899×10^{5}	2.821×10^{-3}	14.9	2.343×10^{5}	5.984×10^{-2}	255.4	3.031×10^{5}	2.863×10^{-3}	9.4	3.442×10^{5}	8.469×10^{-2}	246.0	-	-	No binding

Table S2. Kinetic parameters of the interactions between hDPP4 and merbecovirus S-proteins and monomeric RBDs in BLI assays (relating to Fig. S2)

	k_{on} ($M^{-1}S^{-1}$)	k_{off} (S^{-1})	K_D (nM)		k_{on} ($M^{-1}S^{-1}$)	k_{off} (S^{-1})	K_D (nM)		k_{on} ($M^{-1}S^{-1}$)	k_{off} (S^{-1})	K_D (nM)		k_{on} ($M^{-1}S^{-1}$)	k_{off} (S^{-1})	K_D (nM)
**MERS -2P-CoV-S**	1.458×10^4 (k_{on1})<br>4.650×10^4 (k_{on2})	1.778×10^{-4} (k_{off1})<br>$<1\times10^{-7}$ (k_{off2})	12.2 (k_{off1}/k_{on1})<br>3.8 (k_{off1}/k_{on2})<br><0.0069 (k_{off2}/k_{on1})	**GD-BatCoV-S**	6.308×10^3 (k_{on1})<br>1.249×10^4 (k_{on2})	4.506×10^{-3} (k_{off1})<br>6.579×10^{-5} (k_{off2})	714.3 (k_{off1}/k_{on1})<br>360.8 (k_{off1}/k_{on2})<br>10.4 (k_{off2}/k_{on1})<br>5.3 (k_{off2}/k_{on2})	**SE-Pangolin CoV-S**		Not available		**HKU4-2P-BatCoV-S**	2.201×10^4 (k_{on1})<br>1.093×10^5 (k_{on2})	1.723×10^{-2} (k_{off1})<br>4.913×10^{-4} (k_{off2})	728.3 (k_{off1}/k_{on1})<br>157.6 (k_{off1}/k_{on2})<br>22.3 (k_{off2}/k_{on1})<br>4.5 (k_{off2}/k_{on2})
**HKU25-2P-BatCoV-S**	-	-	No binding	**CN-Hedgehog CoV-S**	-	-	No binding	**EU-Hedgehog CoV-S**	-	-	No binding	**SA-BatCoV-S**	-	-	No binding
**HKU5-BatCoV-S**	-	-	No binding	**Japan-BatCoV-S**	-	-	No binding	**SC-BatCoV-S**	-	-	No binding	**GHA-BatCoV-S**	-	-	No binding
**MERS -CoV-RBD**	4.100×10^5	1.967×10^{-1}	479.8	**GD-BatCoV-RBD**	-	-	Weak binding	**SE-Pangolin CoV-RBD**	9.958×10^5	1.524×10^{-2}	15.3	**HKU4-BatCoV-RBD**	-	-	No binding
**HKU25-BatCoV-RBD**	-	-	No binding												

Table S3. Kinetic parameters of the interactions between hDPP4 and GD-BatCoV-RBD-Fc mutants in BLI assays (relating to Fig. 2B)

	k_{on} ($M^{-1}S^{-1}$)	k_{off} (S^{-1})	K_D (nM)		k_{on} ($M^{-1}S^{-1}$)	k_{off} (S^{-1})	K_D (nM)		k_{on} ($M^{-1}S^{-1}$)	k_{off} (S^{-1})	K_D (nM)		k_{on} ($M^{-1}S^{-1}$)	k_{off} (S^{-1})	K_D (nM)
**GD-BatCoV-RBD-Fc**	2.343×10^{5}	5.984×10^{-2}	255.4	**GD-BatCoV-K505A-RBD-Fc**	3.736×10^{5}	1.770×10^{-1}	473.8	**GD-BatCoV-N513A-RBD-Fc**	3.766×10^{5}	1.044×10^{-1}	277.2	**GD-BatCoV-Q540A-RBD-Fc**	3.122×10^{5}	1.039×10^{-1}	332.8
**GD-BatCoV-D541A-RBD-Fc**	-	-	No binding	**GD-BatCoV-T546R-RBD-Fc**	-	-	No binding	**GD-BatCoV-I557W-RBD-Fc**	2.340×10^{5}	3.318×10^{-2}	141.8	**GD-BatCoV-S539W-RBD-Fc**	2.145×10^{5}	6.063×10^{-2}	282.7
**GD-BatCoV-Q540E-RBD-Fc**	2.189×10^{5}	7.230×10^{-2}	330.3	**GD-BatCoV-S539W-T546R-I557W-RBD-Fc**	-	-	No binding								

Table S4. Kinetic parameters of the interactions between hDPP4 and SE-PangolinCoV-RBD-Fc mutants in BLI assays (relating to Fig. 2D)

	k_{on} ($M^{-1}S^{-1}$)	k_{off} (S^{-1})	K_D (nM)		k_{on} ($M^{-1}S^{-1}$)	k_{off} (S^{-1})	K_D (nM)		k_{on} ($M^{-1}S^{-1}$)	k_{off} (S^{-1})	K_D (nM)		k_{on} ($M^{-1}S^{-1}$)	k_{off} (S^{-1})	K_D (nM)
**SE-PangolinCoV-RBD-Fc**	3.031×10^5	2.863×10^{-3}	9.4	**SE-PangolinCoV-F507I-Q508S-RBD-Fc**	2.019×10^5	8.377×10^{-3}	41.5	**SE-PangolinCoV-H517N-N518Q-S519D-RBD-Fc**	2.210×10^5	1.190×10^{-2}	53.8	**SE-PangolinCoV-Y540G-RBD-Fc**	2.057×10^5	3.880×10^{-3}	18.9
**SE-PangolinCoV-R550T-RBD-Fc**	1.727×10^5	1.036×10^{-3}	6.0	**SE-PangolinCoV-F507I-Q508S-Y540G-RBD-Fc**	1.271×10^5	1.476×10^{-2}	116.1	**SE-PangolinCoV-H517N-N518Q-S519D-Y540G-RBD-Fc**	1.211×10^5	2.501×10^{-2}	206.5	**SE-PangolinCoV-F507I-Q508S-H517N-N518Q-S519D-RBD-Fc**	1.449×10^5	3.057×10^{-2}	211.0
**SE-PangolinCoV-F507I-Q508S-H517N-N518Q-S519D-Y540G-RBD-Fc**	1.936×10^5	1.134×10^{-1}	585.7	**SE-PangolinCoV-(HKU4-RBM)-RBD-Fc**	-	-	weak binding								

Table S5. Kinetic parameters of the interactions between hDPP4 or hDPP4-N229D and merbecovirus RBD-Fc in BLI assays (relating to Fig. 2F)

	**MERS-CoV-RBD-Fc**			**GD-BatCoV-RBD-Fc**			**SE-PangolinCoV-RBD-Fc**			**HKU4-BatCoV-RBD-Fc**		
	k_{on} ($M^{-1}S^{-1}$)	k_{off} (S^{-1})	K_D (nM)	k_{on} ($M^{-1}S^{-1}$)	k_{off} (S^{-1})	K_D (nM)	k_{on} ($M^{-1}S^{-1}$)	k_{off} (S^{-1})	K_D (nM)	k_{on} ($M^{-1}S^{-1}$)	k_{off} (S^{-1})	K_D (nM)
**hDPP4-N229D**	-	-	weak binding	3.556×10^5	1.541×10^{-1}	433.3	2.178×10^5	2.436×10^{-2}	111.8	-	-	No binding
**hDPP4**	1.899×10^5	2.821×10^{-3}	14.9	2.343×10^5	5.984×10^{-2}	255.4	3.031×10^5	2.863×10^{-3}	9.4	3.442×10^5	8.469×10^{-2}	246.0

Table S6. Kinetic parameters of the interactions between hDPP4 and RBM fragment-swapped GD-BatCoV-RBD or MERS-CoV-RBD mutants in BLI assays (relating to Fig. 3 and Fig. S9)

	k_{on} ($M^{-1}S^{-1}$)	k_{off} (S^{-1})	K_D (nM)		k_{on} ($M^{-1}S^{-1}$)	k_{off} (S^{-1})	K_D (nM)		k_{on} ($M^{-1}S^{-1}$)	k_{off} (S^{-1})	K_D (nM)		k_{on} ($M^{-1}S^{-1}$)	k_{off} (S^{-1})	K_D (nM)
**GD-BatCoV-RBD-Fc**	2.343×10^{5}	5.984×10^{-2}	255.4	**GD-BatCoV-MERS-(484-562)-Fc**	1.990×10^{5}	1.154×10^{-2}	58.0	**GD-BatCoV-MERS-(496-506)-Fc**	-	-	weak binding	**GD-BatCoV-MERS-(507-512)-Fc**	-	-	No binding
**GD-BatCoV-MERS-(497-514)-Fc**	-	-	No binding	**GD-BatCoV-MERS-(535-553)-Fc**	2.204×10^{5}	9.306×10^{-2}	422.2	**GD-BatCoV-MERS-(525-553)-Fc**	1.983×10^{5}	4.481×10^{-2}	226.0	**GD-BatCoV-MERS-(525-534)-Fc**	-	-	weak binding
**MERS-CoV-RBD-Fc**	1.899×10^{5}	2.821×10^{-3}	14.9	**MERS-GD-(488-566)-Fc**	1.843×10^{5}	3.568×10^{-2}	193.6	**MERS-GD-(499-509)-Fc**	1.041×10^{5}	3.577×10^{-2}	343.6	**MERS-GD-(510-516)-Fc**	2.106×10^{5}	1.219×10^{-2}	57.9
**MERS-GD-(500-518)-Fc**	2.480×10^{5}	3.164×10^{-2}	127.6	**MERS-GD-(539-557)-Fc**	-	-	No binding	**MERS-GD-(529-557)-Fc**	-	-	No binding	**MERS-GD-(529-538)-Fc**	7.183×10^{4}	3.309×10^{-2}	406.6

Table S7. Domain boundary definitions in amino acid residue numbers for the reported merbecovirus S-trimer structures based on a sequence alignment with the MERS-CoV S-protein (relating to Fig. 4)

	NTD	RBD	Domain C	Domain D	FPPR	Fusion peptide
MERS-CoV	17-351	381-587	368-380, 595-653	356-367, 654-772	905-929	941-983
GD-BatCoV	17-355	385-591	372-384, 599-657	360-371, 658-768	901-925	937-979
HKU25-BatCoV	17-359	389-586	376-388, 594-652	364-375, 653-764	897-921	933-975
Japan-BatCoV	17-356	386-582	373-385, 590-648	361-372, 649-764	897-921	933-975
SA-BatCoV	17-354	384-584	371-383, 593-651	359-370, 652-763	896-920	932-974
CN-HedgehogCoV	17-340	370-574	357-369, 583-641	345-356, 642-751	885-909	921-963
EU-HedgehogCoV	17-339	369-573	356-368, 582-640	344-355, 641-753	886-910	922-964

Table S8. Kinetic parameters of the interactions between hDPP4 and wildtype or I557T mutant of GD-BatCoV-RBD-Fc in BLI assays (relating to Fig. S23)

	**GD-BatCoV-RBD-Fc**			**GD-BatCoV-I557T-RBD-Fc**		
	**k_{on} ($M^{-1}S^{-1}$)**	**k_{off} (S^{-1})**	**K_D (nM)**	**k_{on} ($M^{-1}S^{-1}$)**	**k_{off} (S^{-1})**	**K_D (nM)**
**hDPP4**	2.343×10^{5}	5.984×10^{-2}	255.4	-	-	No binding

Table S9. Cryo-EM data collection, refinement and validation statistics

	GD-BatCoV (PDB 9JMP)	HKU25-BatCoV (PDB 9JMH)	Japan-BatCoV (PDB 9JMO)	GD-BatCoV-RBD:hDPP4 (PDB 9JMJ)	SE-PangolinCoV-RBD:hDPP4 (PDB 9JMM)
**Data collection and processing**					
Magnification	45000×	45000×	45000×	45000×	45000×
Voltage(kv)	200	200	200	200	200
Defocus range (μm)			-0.8~-2.5		-0.8~-1.6
Pixel size (Å)	0.88	0.88	0.88	0.88	0.88
Symmetry imposed	C3	C3	C3	C1	C1
Initial particle images (no.)	1619993	212231	672537	1619993	4686542
Final particle images (no.)	111480	110266	198806	72715	721281
Map resolution (Å)	2.9	3.0	2.8	3.4	2.7
FSC threshold	0.143	0.143	0.143	0.143	0.143
Map resolution range (Å)	2.81-4.81	2.96-8.62	2.68-7.35	3.25-7.54	2.32-33.86
**Refinement**					
Initial model used (PDB code)	6Q04	6Q04	6Q04	4KR0	4KR0
Model resolution (Å)	3.00	3.11	2.83	3.51	2.82
FSC threshold	0.5	0.5	0.5	0.5	0.5
Map sharpening B factor ($Å^2$)	-49	-137.8	-54	-58	-132.2
Model composition					
Non-hydrogen atoms	25407	27452	27694	13813	13763
Protein residues	3195	3363	3420	1656	1645
Ligands	51	79	74	25	27
B factors ($Å^2$)					
Protein	44.89	49.7	47.38	57.86	37.75
Ligand	69.51	73.03	76.19	80.32	63.47
R.m.s. deviations					
Bond lengths (Å)	0.003	0.003	0.002	0.003	0.004
Bond angles (°)	0.638	0.588	0.439	0.570	0.644
**Validation**					
MolProbity score	1.35	1.38	1.20	1.53	1.57
Clash score	2.95	3.37	4.09	4.75	3.76
Poor rotamers (%)	0.22	1.44	1.02	0.40	1.70
Ramachandran plot					
Favored (%)	96.03	97.27	98.22	95.93	96.52
Allowed (%)	3.97	2.73	1.78	4.75	3.48
Disallowed (%)	0.00	0.00	0.00	0.00	0.00

Table S9. (continued) Cryo-EM data collection, refinement and validation statistics

	CN-HedgehogCoV-locked-1 (PDB 9JMN)	CN-HedgehogCoV-locked-2 (PDB 9JMI)	EU-HedgehogCoV (PDB 9JMG)	SA-BatCoV (PDB 9JMF)
**Data collection and processing**				
Magnification	45000×	45000×	45000×	45000×
Voltage(kV)	200	200	200	200
Defocus range (μm)	-0.8~-2.5			
Pixel size (Å)	0.88	0.88	0.88	0.88
Symmetry imposed	C3	C3	C3	C3
Initial particle images (no.)	272109	272109	504617	474463
Final particle images (no.)	23410	63700	82386	196103
Map resolution (Å)	3.8	3.3	3.0	3.1
FSC threshold	0.143	0.143	0.143	0.143
Map resolution range (Å)	3.58-13.69	3.09-11.83	2.91-4.90	2.95-6.24
**Refinement**				
Initial model used (PDB code)	6Q04	6Q04	6Q04	6Q04
Model resolution (Å)	3.81	3.26	3.12	3.09
FSC threshold	0.5	0.5	0.5	0.5
Map sharpening B factor ($Å^2$)	-95	-100	-84	-74
Model composition				
Non-hydrogen atoms	26833	27732	27340	27750
Protein residues	3330	3394	3378	3441
Ligands	53	81	65	66
B factors ($Å^2$)				
Protein	59.51	45.30	53.00	38.69
Ligand	71.25	74.37	78.33	69.97
R.m.s. deviations				
Bond lengths (Å)	0.003	0.004	0.004	0.004
Bond angles (°)	0.689	0.694	0.775	0.710
**Validation**				
MolProbity score	1.51	1.47	1.43	1.48
Clash score	4.39	3.70	4.38	4.38
Poor rotamers (%)	0.21	0.00	0.55	0.17
Ramachandran plot				
Favored (%)	95.74	95.56	96.64	96.15
Allowed (%)	4.26	4.44	3.36	3.85
Disallowed (%)	0.00	0.00	0.00	0.00
